# Supplementary material for: Divergence in neuronal signaling pathways despite conserved neuronal identity among Caenorhabditis species
Source: Curr Biol. Author manuscript; Available in PMC 2025 Aug 11. (PMC7617994; doi:10.1016/j.cub.2025.05.036)
Supplement: Document S1. [file EMS207345-supplement-Document_S1_.pdf]

Current Biology, Volume *35*

Supplemental Information

Divergence in neuronal signaling pathways despite conserved neuronal identity among *Caenorhabditis* species

Itai Antoine Toker, Lidia Ripoll-Sánchez, Luke T. Geiger, Antoine Sussfeld, Karan S. Saini, Isabel Beets, Petra E. Vértes, William R. Schafer, Eyal Ben-David, and Oliver Hobert

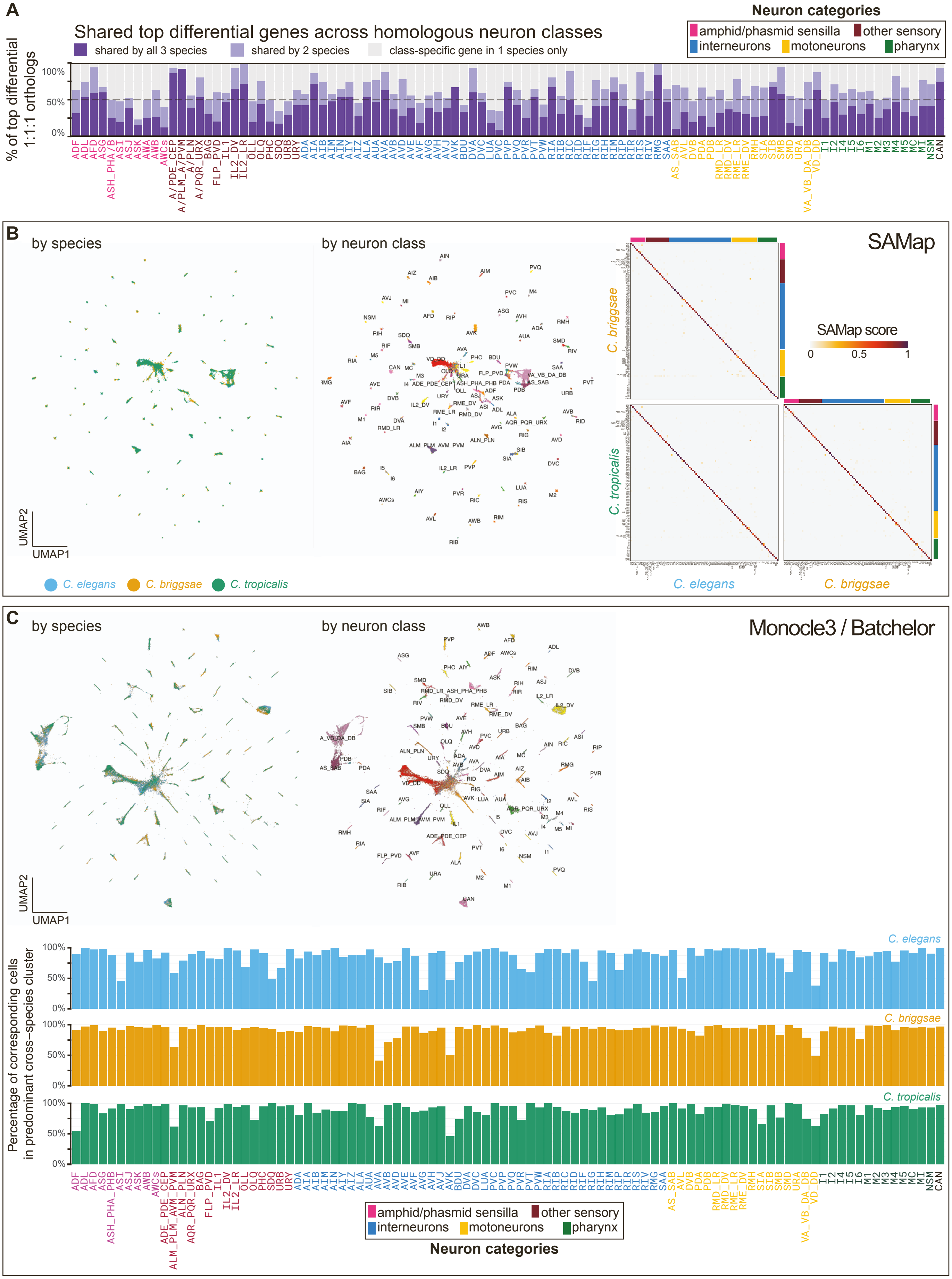
A
Shared top differential genes across homologous neuron classes
shared by all 3 species
shared by 2 species
class-specific gene in 1 species only
% of top differential 1:1:1 orthologs
100%
50%
0%
Neuron categories
amphid/phasmid sensilla
other sensory
interneurons
motoneurons
pharynx
B
by species
by neuron class
SAMap
SAMap score
0
0.5
1
C. briggsae
C. tropicalis
C. elegans
UMAP2
UMAP1
C
Monocle3 / Batchelor
Percentage of corresponding cells in predominant cross-species cluster
Neuron categories

Figure S1. Shared differential genes and cross-species integration support neuronal homology in annotated cross-species datasets. Related to Figure 1.
(**A**) Barplots depicting all 1:1:1 orthologs (y-axis) in the top-10 of differential genes of a neuron class in any of the three species. Colors within bars depict the gene subsets found in the top-20 differential genes in all three species (dark purple), in two species (light purple) or in one species (grey).
(**B**) Integration of scRNA-seq datasets of the three species using SAMap. Left – UMAP projections by species and by neuron class. Right - Heatmap of cell type mapping scores.
(**C**) Integration using Monocle3/Batchelor. Up – UMAP projections by species and by neuron class. Bottom – barplots depicting, in each species (color) and each neuron class (x-axis), the percentage of cells assigned to the “matching” cluster after integration (i.e., the cluster that contains the highest number of cells from the corresponding cell type across all datasets).

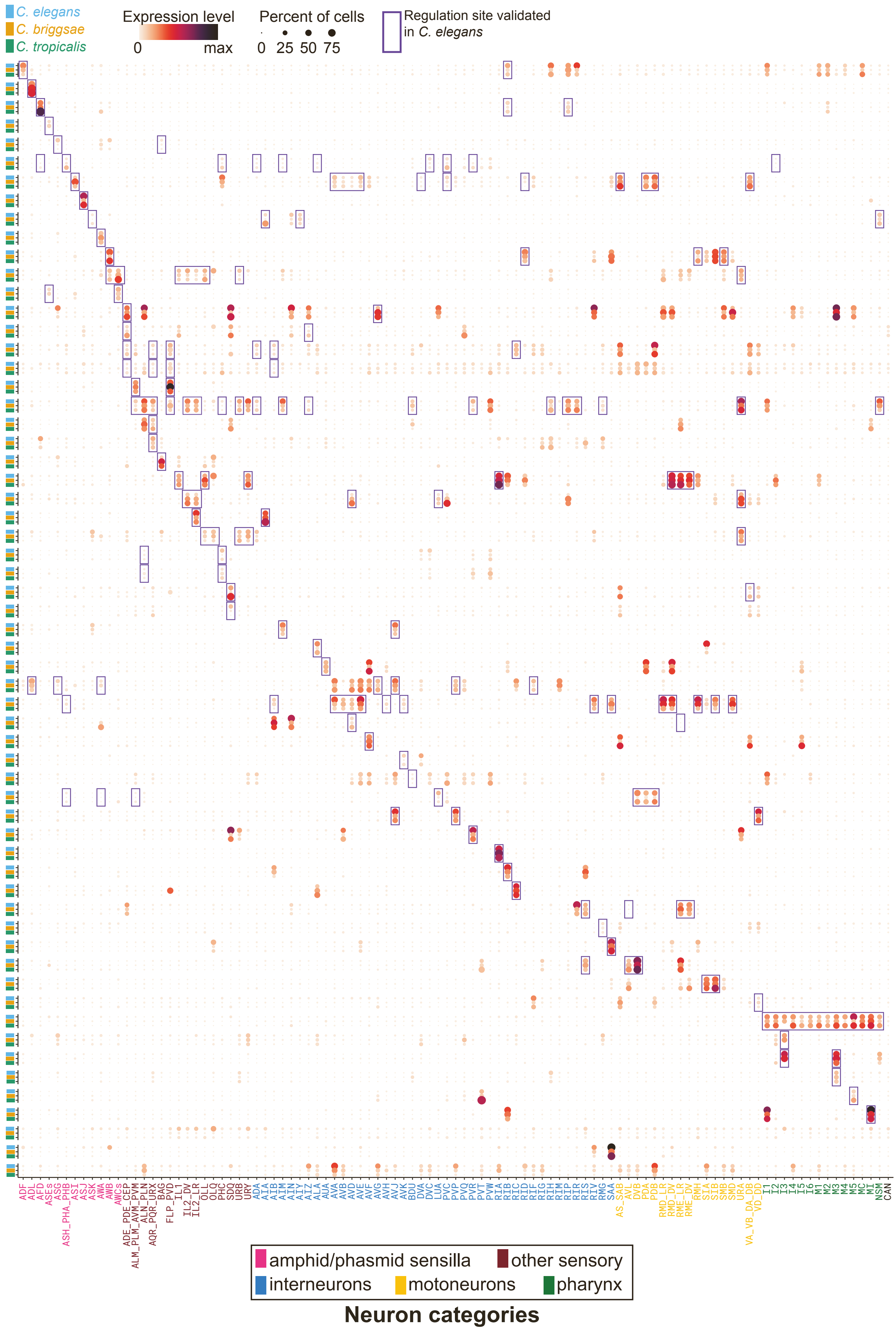

C. elegans
C. briggsae
C. tropicalis
Expression level
0
max
Percent of cells
0 25 50 75
Regulation site validated in C. elegans
lag-1
hlh-4
ttx-1
che-1
ceh-37
ceh-14
unc-3
sptf-1
ttx-3
odr-7
lim-4
sox-2
ceh-36
ast-1
ceh-43
unc-62
ceh-20
mec-3
unc-86
ahr-1
egl-13
ets-5
ceh-32
cfi-1
unc-39
vab-3
nob-1
php-3
lin-39
mab-5
mls-2
ceh-17
ceh-6
lin-11
unc-42
tab-1
unc-4
hlh-15
pag-3
egl-5
unc-30
ceh-31
ceh-8
aptf-1
hlh-13
nhr-67
ceh-13
sox-3
lim-6
ceh-24
elt-1
ceh-34
pros-1
ceh-2
mnm-2
vab-15
ceh-45
egl-18
ceh-9
sem-4
ADF
ADL
AFD
ASEs
ASG
ASH_PHA_PHB
ASI
ASJ
ASK
AWA
AWB
AWCs
ADE_PDE_CEP
ALM_PLM_AVM_PVM
ALN_PLN
AQR_PQR_URX
BAG
FLP_PVD
IL1
IL2_DV
IL2_LR
OLL
OLQ
PHC
SDQ
URB
URY
ADA
AIA
AIB
AIM
AIN
AIY
AIZ
ALA
AUA
AVA
AVB
AVD
AVE
AVF
AVG
AVH
AVJ
AVK
BDU
DVA
DVC
LUA
PVC
PVP
PVQ
PVR
PVT
PVW
RIA
RIB
RIC
RID
RIF
RIG
RIH
RIM
RIP
RIR
RIS
RIV
RMG
SAA
AS_SAB
AVL
DVB
PDA
PDB
RMD_LR
RMD_DV
RME_LR
RME_DV
RMH
SIA
SIB
SMB
SMD
URA
VA_VB_DA_DB
VD_DD
I1
I2
I3
I4
I5
I6
M1
M2
M3
M4
M5
MC
MI
NSM
CAN
amphid/phasmid sensilla
other sensory
interneurons
motoneurons
pharynx
Neuron categories

Figure S2. Expression conservation of transcription factors composing the regulatory codes of neuronal identity in *C. elegans*. Related to Figure 2.

Cross-species expression dotplot of all 1:1:1 transcription factor orthologs functionally validated as identity specifiers in *C. elegans*. Nematode species (y-axis) and neuron class (x-axis) are color-coded according to legend. Dot size represents the fraction of cells expressing the gene in a given neuron class, color represents scaled average expression levels. Experimentally-validated sites of identity regulation by a given transcription factor in *C. elegans* are marked with a rectangle. A subset of genes and cells also appear in Figure 1F. Data for ASE and I3 neurons is available for *C. elegans* and *C. briggsae* datasets only. Data for *ceh-9* (that regulate PVN neurons) and for *egl-18* & *sem-4* (that regulate HSN neurons) are shown, but their corresponding neurons are not part of the dataset. PVN are born during L2, and HSN neurons acquire many of their class-specific identity features in the L4 larval stage.

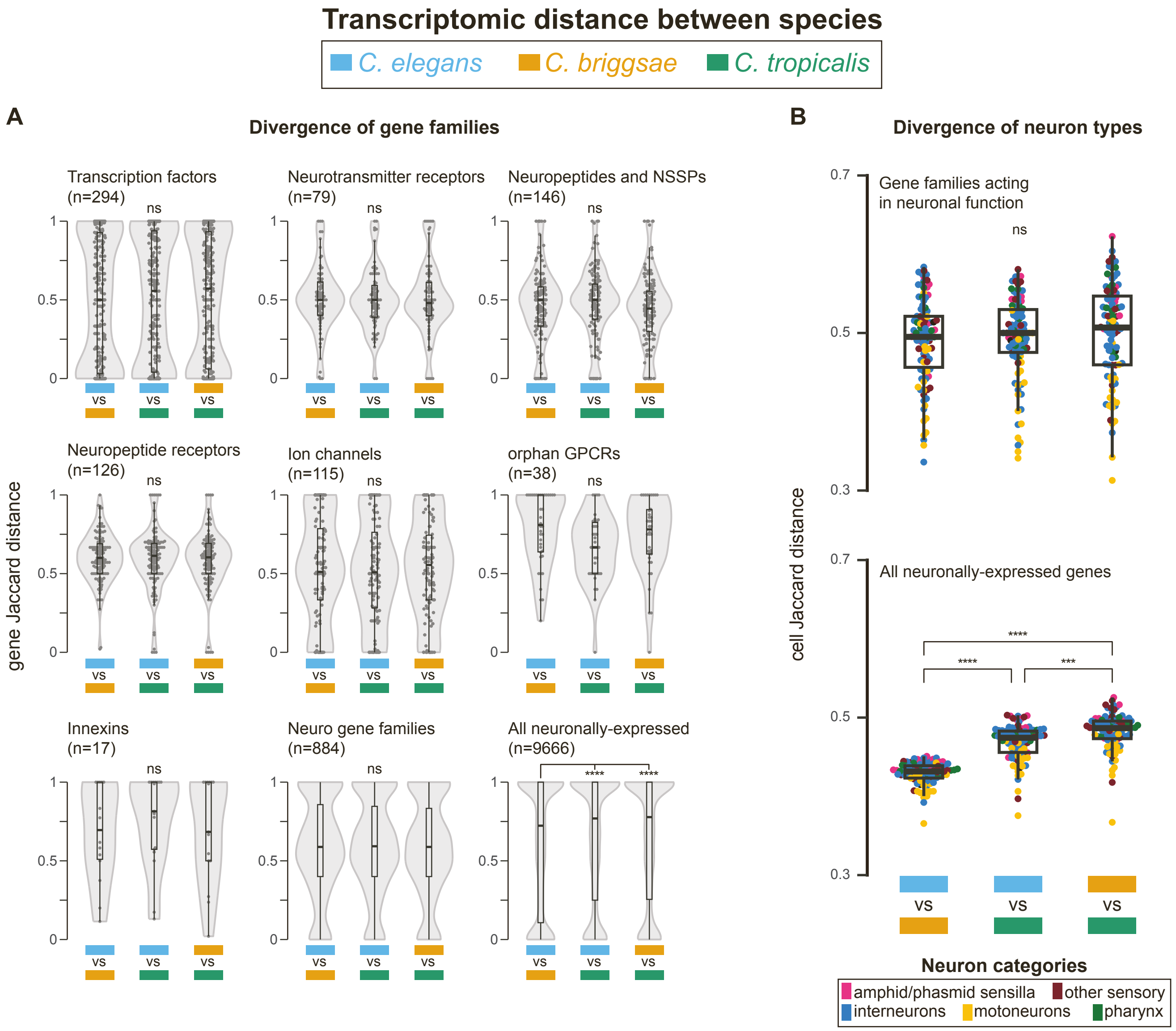

Figure S3. Transcriptomic comparisons between pairs of species. Related to Figure 2.

(A) Jaccard distance (y-axis) of genes (dots) grouped in families, reflecting divergence between pairs of species in their neuron-type-specific gene expression. Inquired gene families appear above corresponding panel. Pairs of species are color-coded below panels. n = number of genes in family, only 1:1 orthologs with expression in at least one neuron type were included. “Transcription factors” included only the families depicted in Figure 2E.

(B) Jaccard distances (y-axis) of neuron classes (dots) grouped into functional categories (x-axis and colors). The calculation of Jaccard distances included (upper panel) to gene families with established function in the nervous system or (lower panel) 9666 genes expressed in at least one species anywhere in the nervous system. Boxplots are Tukey-style. Kruskal-Wallis test, Dunn’s post hoc, Benjamini-Hochberg correction for multiple comparisons. ****$P < 0.0001$, ***$P < 0.001$, **$P < 0.01$, *$P < 0.025$.

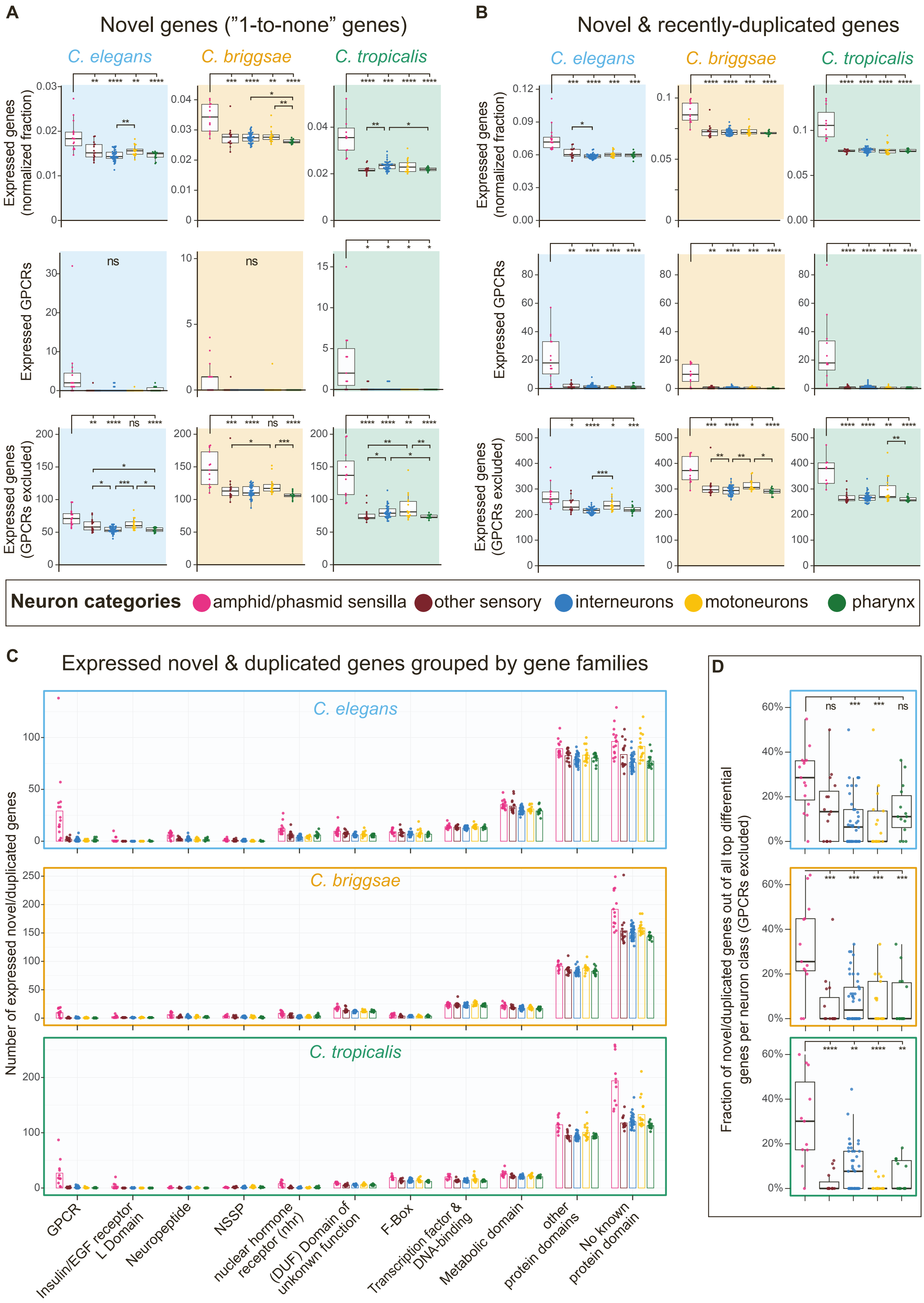
A
Novel genes ("1-to-none" genes)
B
Novel & recently-duplicated genes
C. elegans
C. briggsae
C. tropicalis
Expressed genes (normalized fraction)
Expressed GPCRs
Expressed genes (GPCRs excluded)
ns
Neuron categories
amphid/phasmid sensilla
other sensory
interneurons
motoneurons
pharynx
C
Expressed novel & duplicated genes grouped by gene families
Number of expressed novel/duplicated genes
GPCR
Insulin/EGF receptor L Domain
Neuropeptide
NSSP
nuclear hormone receptor (nhr)
(DUF) Domain of unkonwn function
F-Box
Transcription factor & DNA-binding
Metabolic domain
other protein domains
No known protein domain
D
Fraction of novel/duplicated genes out of all top differential genes per neuron class (GPCRs excluded)

Figure S4. Enrichment of novel and duplicated genes in sensory neurons of the amphid and phasmid sensilla. Related to Figure 3.

(**A)** Expression of novel genes (“1-to-none” orthologs, y-axis) in different neuron classes and species (thresholded expression). Top panels: fraction of expressed novel genes normalized to the total number of expressed genes per cell type. Middle panel: number of novel GPCR genes expressed per cell type. Bottom panels: number of expressed genes after exclusion of all GPCRs from analysis. Neuron classes were grouped into functional categories (x-axis and color coded), each dot represents a single neuron class. Boxplots are Tukey-style.

(**B**) Expression of novel and recently-duplicated genes (“1-to-none”, “1-to-many”, “many-to-many” orthologs) in different neuron classes and species. Panels similar to (A).

(**C**) Number of expressed novel and recently-duplicated genes (y-axis) belonging to different gene families (x-axis) based on sequence homology or the presence of key protein domains. Neuron classes were grouped into functional categories, each dot represents a single neuron class, bars depict mean values for all neurons in category. Genes with no domains detected in PFAM are in the category “no known protein domain”.

(**D**) Proportions of novel and duplicated genes out of all differentially-expressed genes per neuron class, grouped by neuron functional category. All GPCRs were excluded from the analysis.

Statistical tests: Kruskal-Wallis test, Dunn’s post hoc, Benjamini-Hochberg correction for multiple comparisons. ****$P < 0.0001$, ***$P < 0.001$, **$P < 0.01$, *$P < 0.025$.

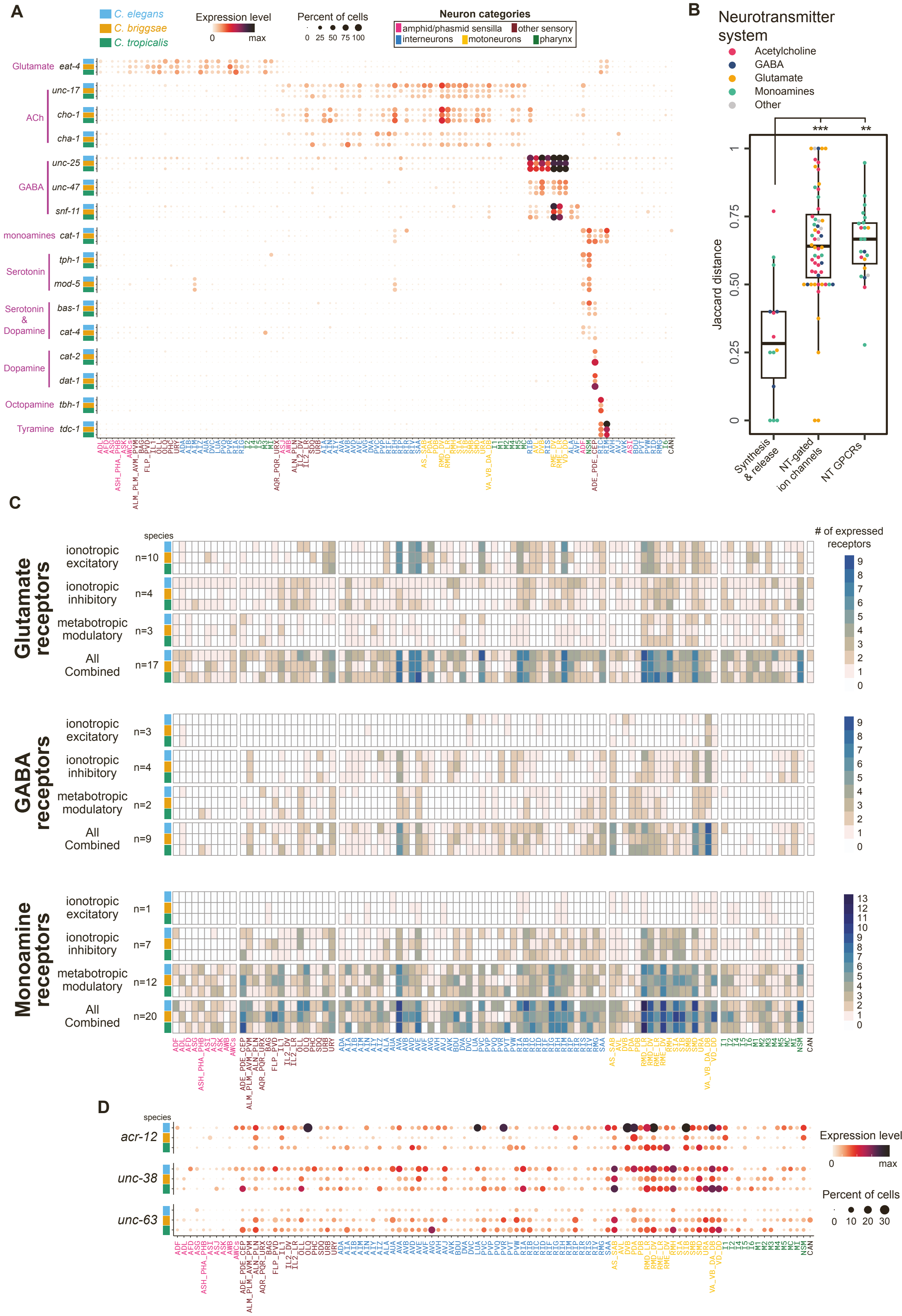

A
C. elegans
C. briggsae
C. tropicalis
Expression level
0
max
Percent of cells
0 25 50 75 100
Neuron categories
amphid/phasmid sensilla
other sensory
interneurons
motoneurons
pharynx
Glutamate
eat-4
ACh
unc-17
cho-1
cha-1
GABA
unc-25
unc-47
snf-11
monoamines
cat-1
Serotonin
tph-1
mod-5
Serotonin & Dopamine
bas-1
cat-4
Dopamine
cat-2
dat-1
Octopamine
tbh-1
Tyramine
tdc-1
B
Neurotransmitter system
Acetylcholine
GABA
Glutamate
Monoamines
Other
Jaccard distance
0
0.25
0.50
0.75
1
***
**
Synthesis & release
NT-gated ion channels
NT GPCRs
C
species
Glutamate receptors
GABA receptors
Monoamine receptors
ionotropic excitatory
ionotropic inhibitory
metabotropic modulatory
All Combined
n=10
n=4
n=3
n=17
n=3
n=4
n=2
n=9
n=1
n=7
n=12
n=20
# of expressed receptors
D
species
acr-12
unc-38
unc-63
Expression level
0
max
Percent of cells
0 10 20 30

Figure S5. Neuron-type-specific expression of gene modules determining the identity of emitted and received neurotransmitters across species. Related to Figure 4.

(**A)** Cross-species expression dotplot of neurotransmitter synthesis genes, neurotransmitter vesicular transporter genes and neurotransmitter uptake transporter genes determining neurotransmitter release identity of neuronal cell classes. Data for *eat-4*, *unc*-17, *unc-25* & *cat-1* appear in Figure 4B and are shown again here for ease of visualization. Associated neurotransmitters are labeled in purple (y-axis). Ach: Acetylcholine; GABA: Gamma-aminobutyric acid. Nematode species (y-axis) and neuron class (x-axis) are color-coded according to legend. Dot size represents the fraction of cells expressing the gene in a given neuron class, color represents scaled average expression levels.

(**B**) Jaccard distances of 1:1:1 ortholog genes determining neurotransmitter release identity and receptivity to neurotransmitters. Each dot represents a gene, boxplots are Tukey-style. Data is a subset of the data already appearing in Figure 4A, but each gene is color-coded here according to the neurotransmitter system to which it belongs. Kruskal-Wallis test, Dunn's post hoc, Benjamini-Hochberg correction for multiple comparisons. ***$P < 0.001$, **$P < 0.01$.

(**C)** Heatmaps representing the number (color-coded) of neurotransmitter receptors expressed in each neuron class (x-axis) across species (y-axis). Subtype of receptors (ionotropic excitatory, ionotropic inhibitory, metabotropic modulatory) are indicated to the left of heatmaps. N values indicate the total number of receptors (1:1:1 orthologs) included in the analysis for a given subtype. Top – Glutamate receptors; Middle – GABA receptors; Bottom – Monoamine receptors.Receptors for all known monoamines acting in *C. elegans* (serotonin, dopamine, tyramine, octopamine) were considered combined together as a single category.

(**D**) Cross-species expression dotplot of the acetylcholine receptors *acr-12, unc-38* & *unc-63*. These receptors are broadly expressed throughout the nervous system.

A

NPP-GPCR	*C. elegans*	*C. briggsae*	*C. tropicalis*
NLP-13 DMSR-2	pervasive	integrative	pervasive
FLP-1 DMSR-6	local	local	broadcaster
FLP-4 DMSR-7	broadcaster	pervasive	pervasive
FLP-7 DMSR-7	pervasive	broadcaster	pervasive
FLP-12 DMSR-8	local	broadcaster	broadcaster
FLP-4 EGL-6	local	integrative	integrative
FLP-6 EGL-6	local	integrative	integrative
FLP-8 FRPR-15	local	local	broadcaster
FLP-3 FRPR-16	broadcaster	local	local
FLP-1 FRPR-7	local	broadcaster	broadcaster
FLP-4 FRPR-8	local	integrative	integrative
FLP-21 NPR-1	local	broadcaster	broadcaster
FLP-21 NPR-2	broadcaster	local	local
FLP-15 NPR-3	pervasive	integrative	pervasive
NLP-10 NPR-35	local	integrative	integrative
NLP-13 NPR-41	integrative	pervasive	no network
NLP-38 SPRR-2	integrative	pervasive	pervasive
NLP-58 TKR-1	local	broadcaster	integrative
NLP-58 TKR-2	local	local	integrative

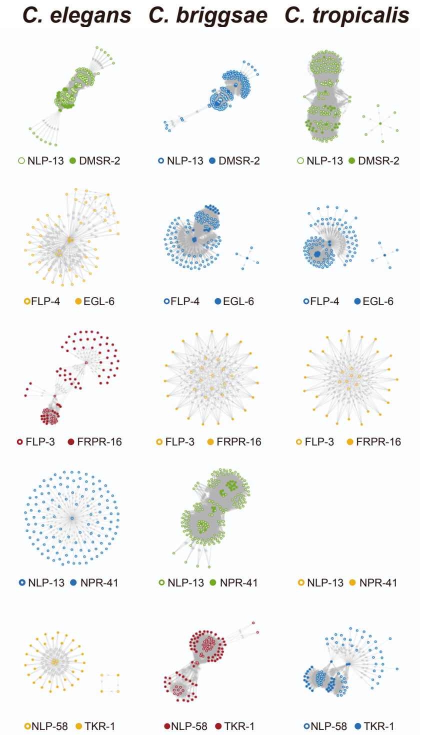

B

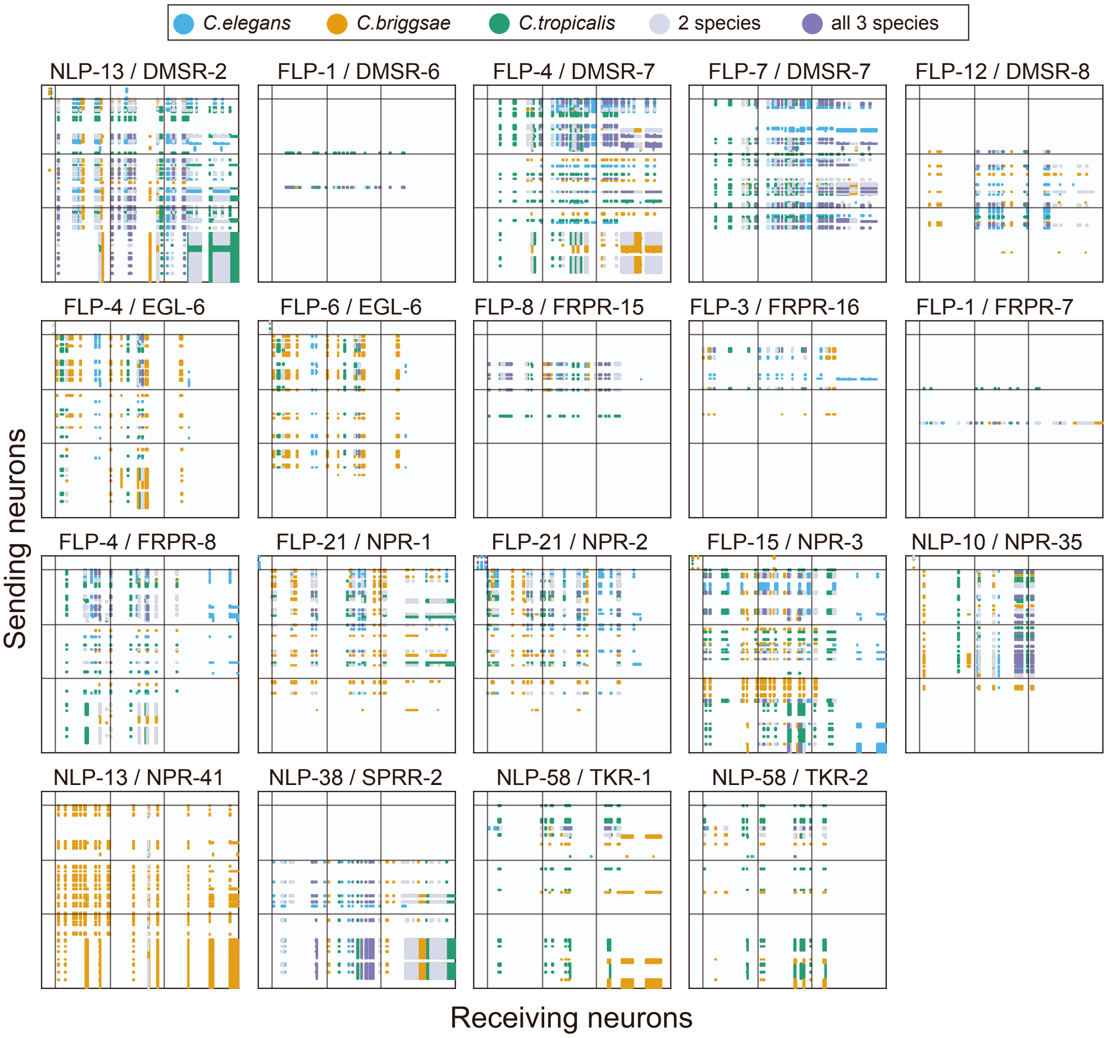

Figure S6. Neuropeptide-receptor pairs displaying divergent network topologies across species. Related to Figure 5.
(**A**) 19 NPP-GPCR pairs displayed divergent topologies across species. Local networks (yellow) express the NPP and GPCR in ≤50 neurons. Pervasive networks (green) express both in ≥50 neurons. Broadcasting networks (red) express the NPP in ≤50 and the GPCR in ≥50. Integrative networks (blue) express the NPP ≥50 neurons and the GPCR in ≤50. Right: Graph visualizations of 4 NPP-GPCR networks across species.
(**B**) Adjacency matrix representation of the 19 individual NPP-GPCR pairs described above (short-range networks). Rows, columns and separations are similar to Figure 5E.

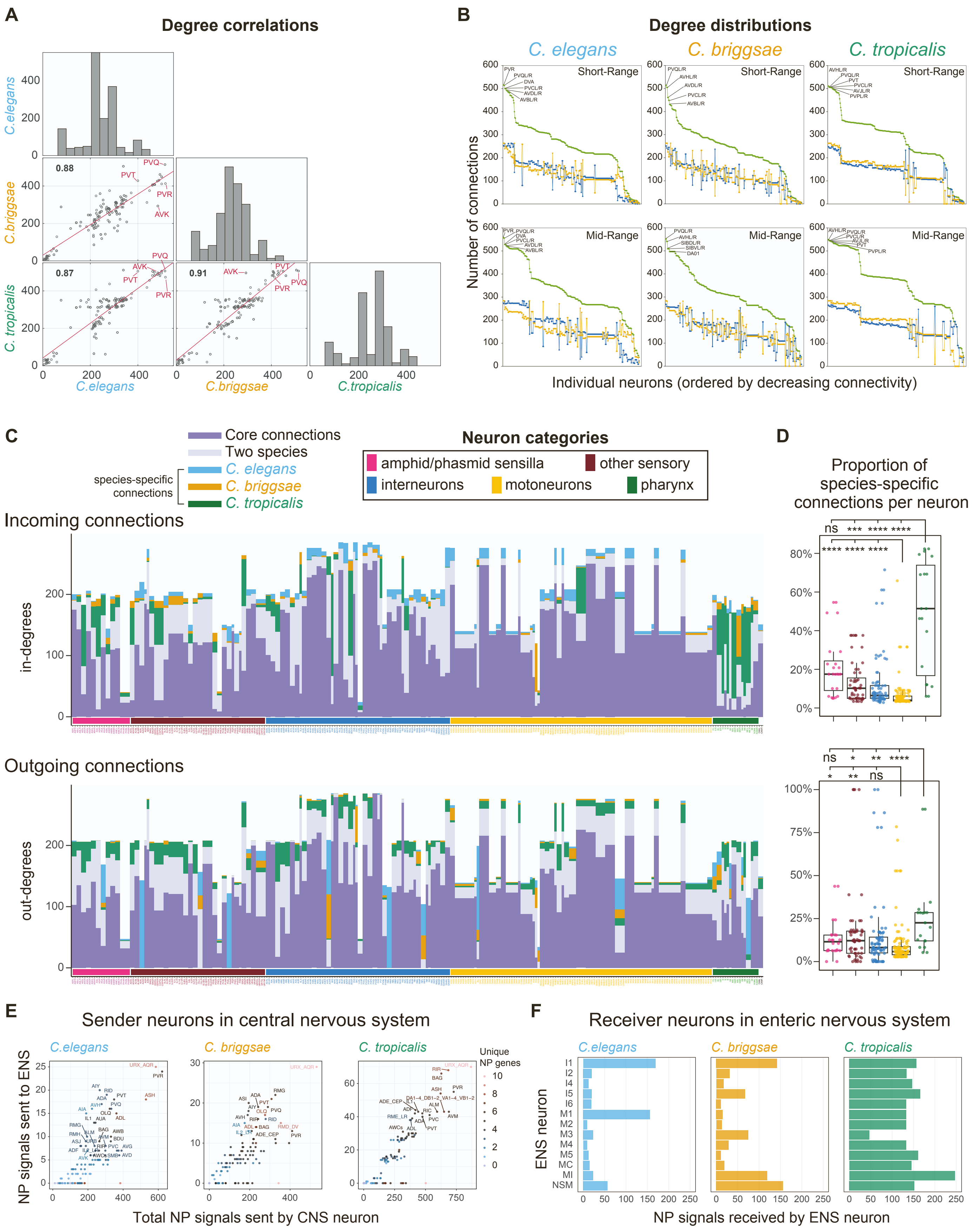

A
Degree correlations
C.elegans
C.briggsae
C. tropicalis
0.88
0.87
0.91
PVQ
PVT
PVR
AVK
C.elegans
C.briggsae
C.tropicalis
B
Degree distributions
C. elegans
C. briggsae
C. tropicalis
Short-Range
Mid-Range
Number of connections
Individual neurons (ordered by decreasing connectivity)
C
Core connections
Two species
species-specific connections
C. elegans
C. briggsae
C. tropicalis
Neuron categories
amphid/phasmid sensilla
other sensory
interneurons
motoneurons
pharynx
Incoming connections
in-degrees
Outgoing connections
out-degrees
D
Proportion of species-specific connections per neuron
ns
E
Sender neurons in central nervous system
C.elegans
C. briggsae
C. tropicalis
Unique NP genes
NP signals sent to ENS
Total NP signals sent by CNS neuron
F
Receiver neurons in enteric nervous system
C.elegans
C. briggsae
C. tropicalis
ENS neuron
I1
I2
I4
I5
I6
M1
M2
M3
M4
M5
MC
MI
NSM
NP signals received by ENS neuron

Figure S7. Degree analysis and communication between central and enteric nervous systems through neuropeptidergic networks. Related to Figure 5. Peptidergic degree is defined as the number of incoming and outgoing connections per neuron (the sum of in-degrees and out-degrees).

(**A**) Pearson correlations of degrees between homologous neuron classes in pairs of species (short-range networks). The conserved peptidergic hubs are highlighted in red (AVK, PVR, PVQ, PVT).

(**B**) Distributions of degrees in the neuropeptidergic networks of three nematode species. Top panels: short-range networks. Bottom panels: mid-range networks. Degree (incoming plus outgoing connections) is shown in green, in-degree (incoming connections) in blue and out-degree (outgoing connections) in yellow. The 10 highest-degree hubs in each network are indicated.

(**C**) Total number of degrees (y-axis) in homologous neurons across species (x-axis). Top panel: in-degrees. Bottom panel: out-degrees. Bars are color-filled according to the subsets of core degrees and species-specific degrees of the neuron across species.

(**D**) Proportions of species-specific degrees (y-axis) per neuron classified by functional categories (colors and x-axis). Kruskal-Wallis test, Dunn's post hoc, Benjamini-Hochberg correction for multiple comparisons. ****$P < 0.0001$, ***$P < 0.001$, **$P < 0.01$, *$P < 0.025$.

(**E**) Weighted numbers of neuropeptide signals sent from neurons in the central nervous system to the enteric nervous system (y-axis) vs total numbers sent overall (x-axis). Coloring of dots represent the number of unique neuropeptide genes which bind GCPRs that are expressed in the enteric nervous system.

(**F**) Number of received neuropeptide signals per neuron type in the enteric nervous system.

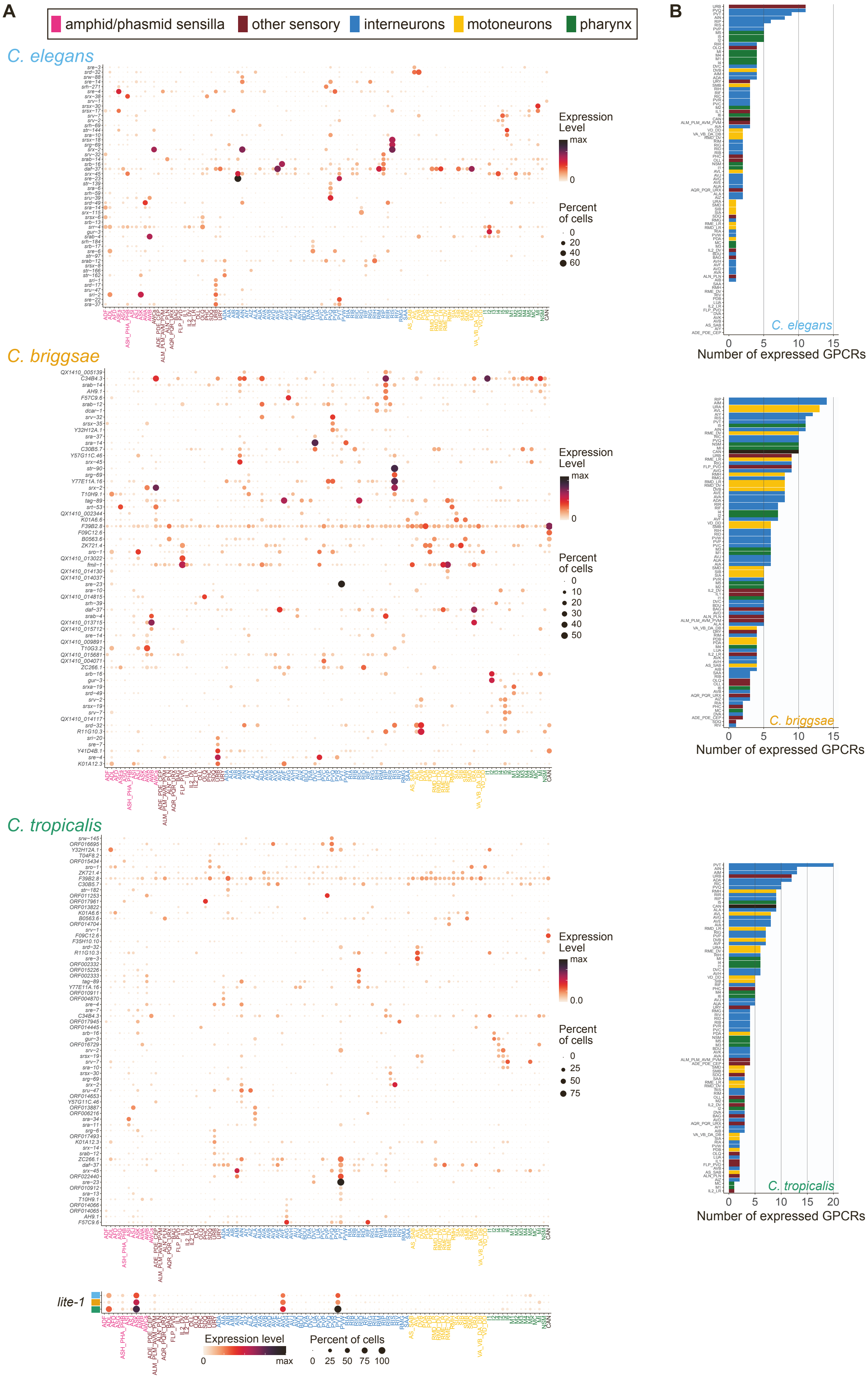

A
amphid/phasmid sensilla
other sensory
interneurons
motoneurons
pharynx
C. elegans
Expression Level
max
0
Percent of cells
0
20
40
60
C. briggsae
Expression Level
max
0
Percent of cells
0
10
20
30
40
50
C. tropicalis
Expression Level
max
0.0
Percent of cells
0
25
50
75
lite-1
Expression level
0
max
Percent of cells
0
25
50
75
100
B
C. elegans
Number of expressed GPCRs
C. briggsae
Number of expressed GPCRs
C. tropicalis
Number of expressed GPCRs

Figure S8. GPCRs expressed in non-sensory neurons. Related to Figure 6.
(**A**) Expression dotplots of GPCR genes that are expressed outside of the amphid/phasmid sensilla and have no sequence homology with neuropeptide- and neurotransmitter-binding GPCRs. 51 GPCRs pass the criteria in *C. elegans*, 63 in *C. briggsae* and 69 in *C. tropicalis*. In the *C. briggsae* and *C. tropicalis* panels, 1:1 orthologs of *C. elegans* genes appear with the *C. elegans* gene name (y-axis). Bottom: The light-responsive *lite-1* GPCR of the gustatory-receptor family is expressed at much higher levels than all other listed GPCRs, and is displayed separately. Dot size represents the fraction of cells expressing the gene in a given neuron class, color represents scaled average expression levels.
(**B**) Barplots indicating how many of these GPCRs are expressed in each neuron class. Bars are colored according functional categories, sensory neurons of the amphid and phasmid sensilla were excluded.

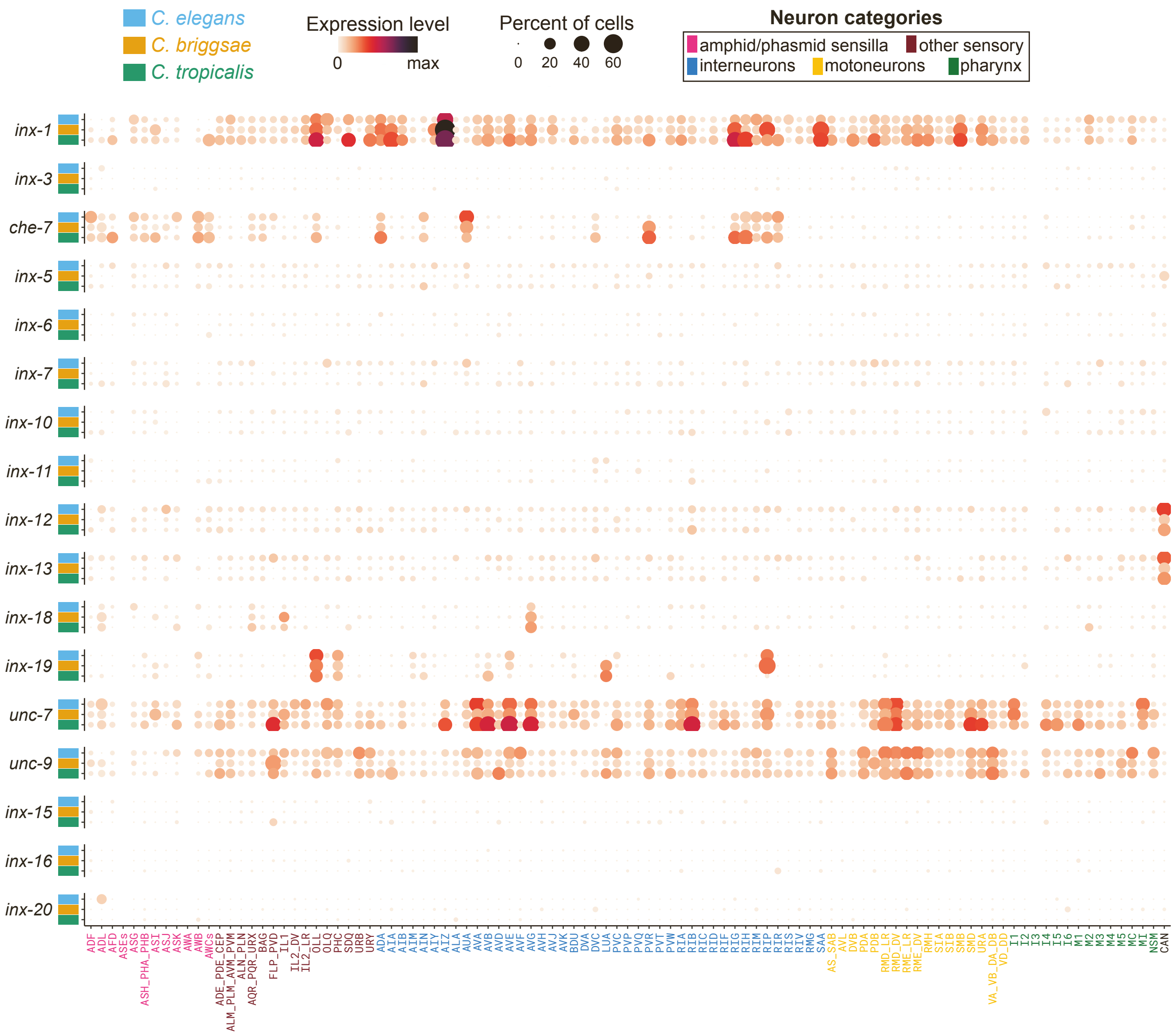

Figure S9. Expression profile of innexins in the nervous system. Related to Figure 6.

Cross-species expression dotplot of innexin 1:1:1 orthologs in the nervous system. Genes with no expression in the nervous system were excluded. Nematode species (y-axis) and neuron class (x-axis) are color-coded according to legend. Dot size represents the fraction of cells expressing the gene in a given neuron class, color represents scaled average expression levels.

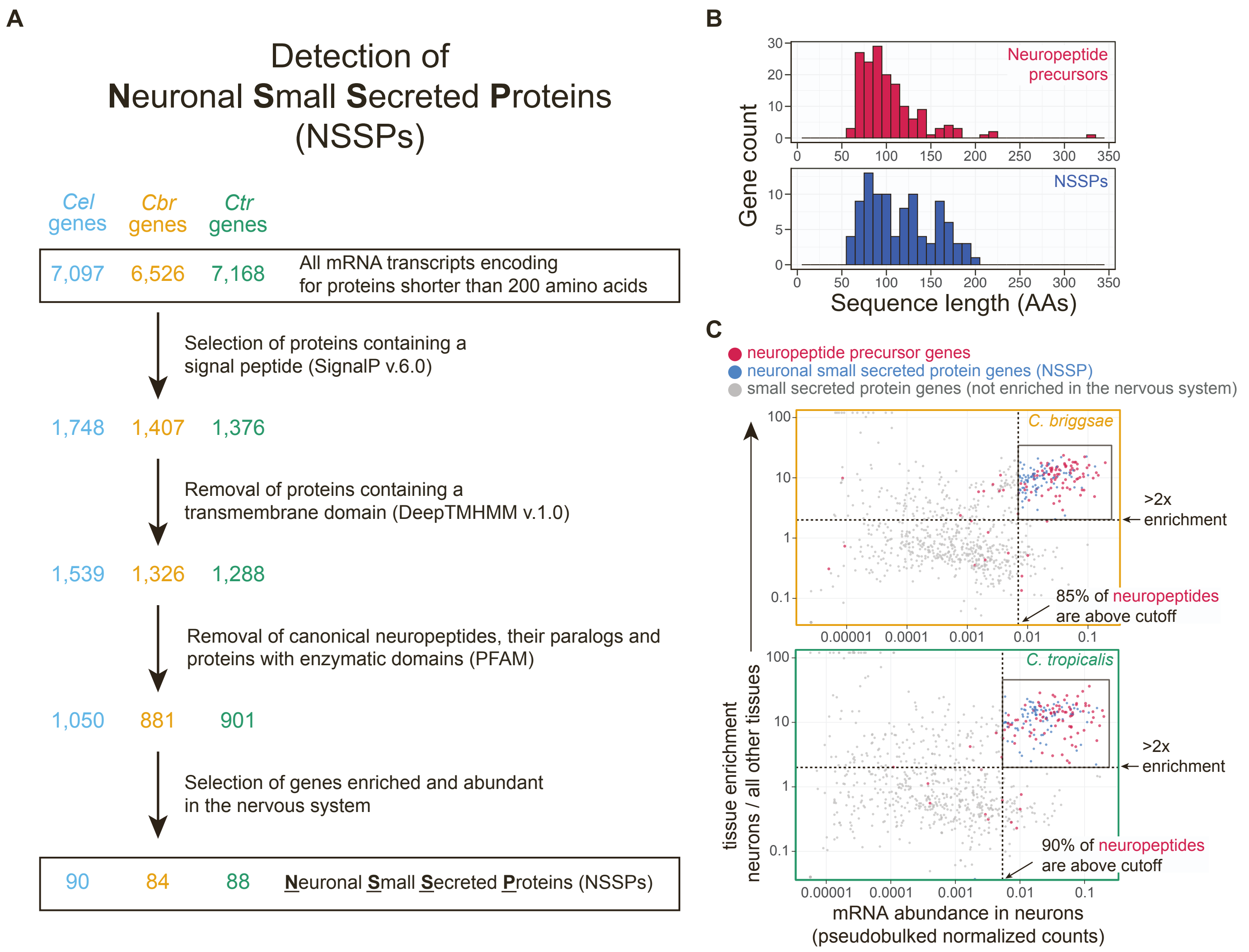

Figure S10. Detection of Neuronal Small Secreted Proteins (NSSPs). Related to Figure 7.
(**A**) Diagram depicting the filtering process defining the pools of NSSPs. Numbers of genes remaining after each step are shown.
(**B**) Distributions of amino acid sequence lengths of all known *C. elegans* neuropeptide precursor genes (red) and newly-defined *C. elegans* NSSPs (blue). Longest mRNA isoform for each gene was used. Only 4/161 (2.5%) of all neuropeptide genes encode for proteins longer than 200 amino acids.
(**C**) Neuronal enrichment (y-axis) and neuronal expression levels (x-axis) of genes encoding small secreted proteins in *C. briggsae* (top panel) and *C. tropicalis* (bottom panel). Each dot is a gene, neuropeptides (red) and NSSPs (blue) are colored. Dashed lines: cutoff criteria used to delineate NSSPs.

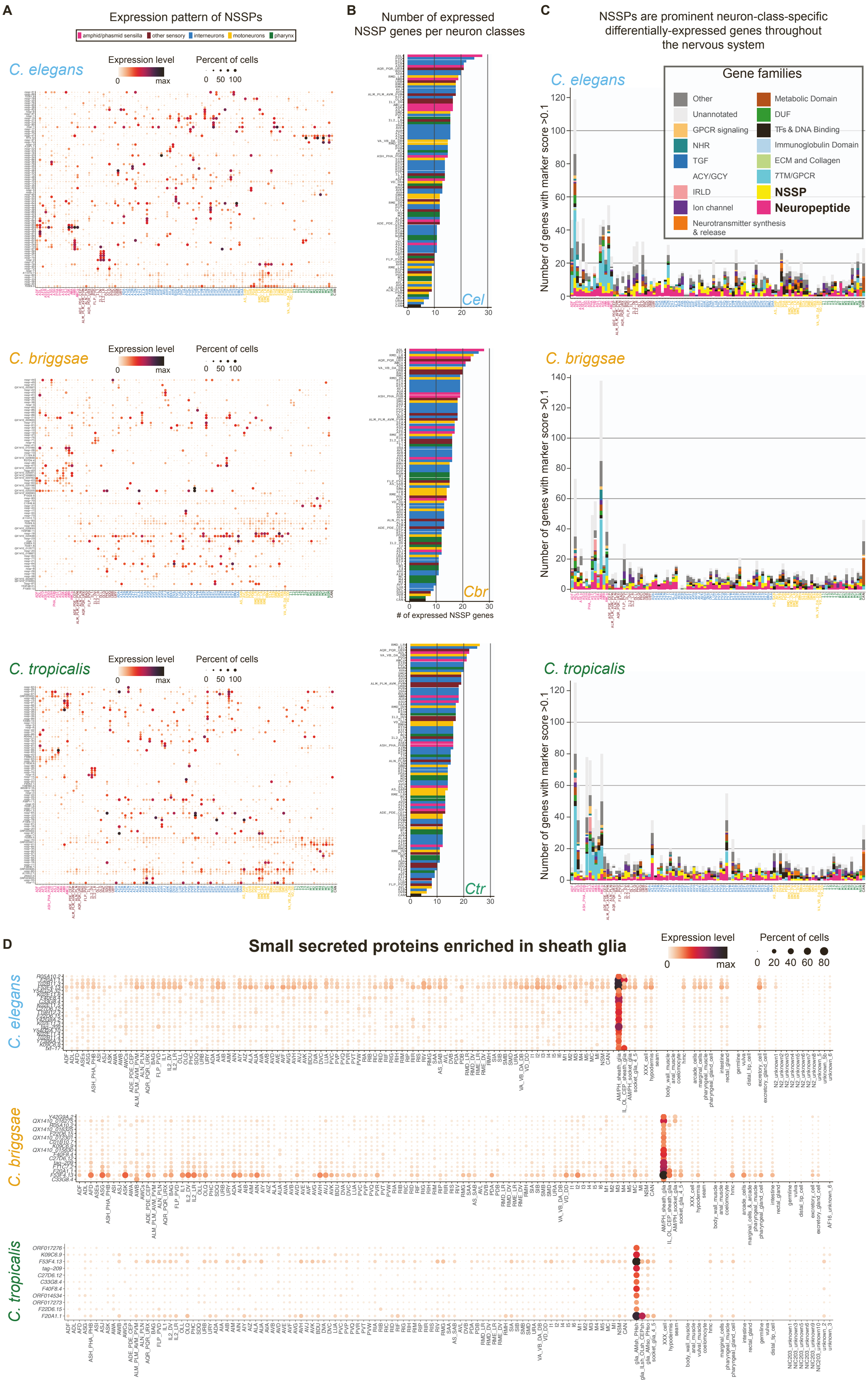
A
Expression pattern of NSSPs
amphid/phasmid sensilla
other sensory
interneurons
motoneurons
pharynx
C. elegans
Expression level
0
max
Percent of cells
0 50 100
B
Number of expressed NSSP genes per neuron classes
Cel
0
10
20
30
C
NSSPs are prominent neuron-class-specific differentially-expressed genes throughout the nervous system
Gene families
Other
Metabolic Domain
Unannotated
DUF
GPCR signaling
TFs & DNA Binding
NHR
Immunoglobulin Domain
TGF
ECM and Collagen
ACY/GCY
7TM/GPCR
IRLD
NSSP
Ion channel
Neuropeptide
Neurotransmitter synthesis & release
Number of genes with marker score >0.1
C. briggsae
Cbr
# of expressed NSSP genes
C. tropicalis
Ctr
D
Small secreted proteins enriched in sheath glia
Percent of cells
0 20 40 60 80

Figure S11. NSSPs are a key distinctive molecular feature in most neuron classes. Related to Figure 7.
(**A**) Expression dotplots of NSSP genes in *C. elegans* (n=90 genes), *C. briggsae* (n=84) and *C. tropicalis* (n=88). In the *C. briggsae* and *C. tropicalis* panels, 1:1 orthologs of *C. elegans* genes appear with the *C. elegans* gene name (y-axis). Dot size represents the fraction of cells expressing the gene in a given neuron class, color represents scaled average expression levels.
(**B**) Barplots showing how many NSSPs are expressed in each neuron class. Bars are colored according functional categories of neurons.
(**C**) Barplots representing the numbers (y-axis) of differentially-expressed genes (*top_markers* score >0.1) expressed in each neuron class (x-axis). Genes with high marker scores tend to be abundantly and specifically expressed in one or few neuron classes. The fraction of genes belonging to specific gene families are colored according to legend. Neuropeptides (**magenta**) and NSSPs (**yellow**) appear among the most differentially-expressed genes in a majority of neuron classes throughout the nervous system. "NHR": nuclear hormone receptor; "TGF": Transforming Growth Factor family; "ACY/GCY" adenylate and guanylyl cyclases; "IRLD": insulin/EGF receptor L Domain family; "DUF": Domain of Unknown Function, "ECM": extracellular matrix, "Unannotated": genes with no domains detected by PFAM.
(**D**) Expression dotplots of small secreted proteins with no characterized protein domain and enriched in sheath glia. In the *C. briggsae* and *C. tropicalis* panels, 1:1 orthologs of *C. elegans* genes appear with the *C. elegans* gene name (y-axis). Dot size represents the percentage of cells expressing the gene in a given cell type, color represents scaled average expression levels.
