# Supplementary material for: Divergence in neuronal signaling pathways despite conserved neuronal identity among Caenorhabditis species
Source: Curr Biol. Author manuscript; Available in PMC 2025 Aug 11. (PMC7617994; doi:10.1016/j.cub.2025.05.036)
Supplement: Document S2. [file EMS207345-supplement-Document_S2_.pdf]

Current Biology

Article

Divergence in neuronal signaling pathways despite conserved neuronal identity among *Caenorhabditis* species

Graphical abstract

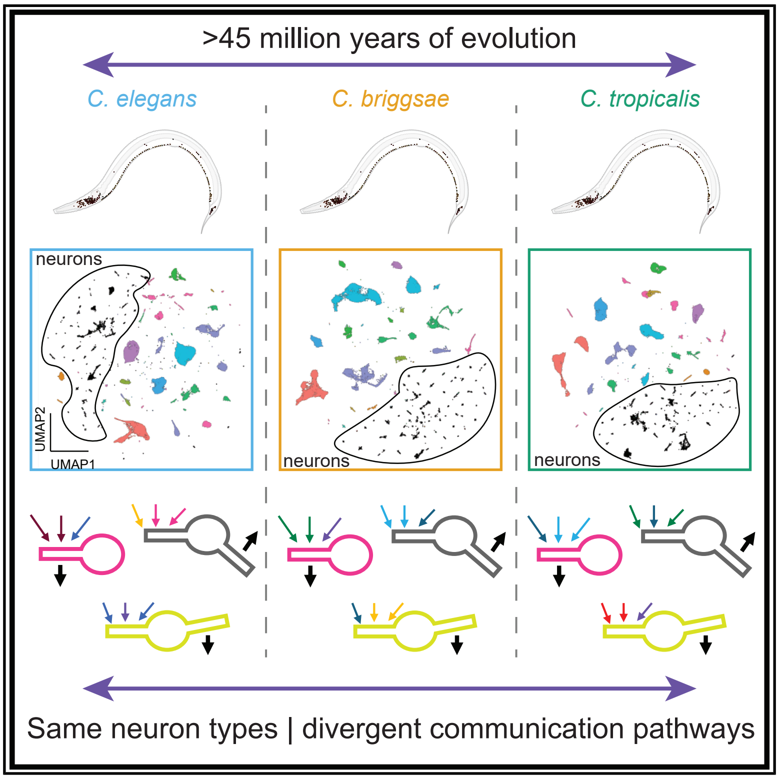

Authors

Itai Antoine Toker,
Lidia Ripoll-Sánchez,
Luke T. Geiger, ..., William R. Schafer,
Eyal Ben-David, Oliver Hobert

Correspondence

it2309@columbia.edu (I.A.T.),
eyal.bendavid@mail.huji.ac.il (E.B.-D.),
or38@columbia.edu (O.H.)

In brief

Comparing the entire brain of related species can reveal patterns of evolutionary change. Toker et al. show that individual neuronal cell types are highly conserved but that expression of neuronal signaling molecules are highly divergent, indicating that neuronal communication pathways are among the most proximal agents of evolutionary change.

Highlights

- scRNA-seq atlases of three known hermaphroditic *Caenorhabditis* species
- Molecular patterns of evolution in homologous neurons throughout the entire brain
- Neuron-specific divergence in neurotransmitter receptors and neuropeptide signaling
- Novel gene family of neuronal small secreted proteins

Toker et al., 2025, Current Biology *35*, 2927–2945
June 23, 2025 © 2025 The Authors. Published by Elsevier Inc.
https://doi.org/10.1016/j.cub.2025.05.036

CellPress

Current Biology

CellPress
OPEN ACCESS

Article

Divergence in neuronal signaling pathways despite conserved neuronal identity among *Caenorhabditis* species

Itai Antoine Toker,[1,*] Lidia Ripoll-Sánchez,[2,3] Luke T. Geiger,[1] Antoine Sussfeld,[1] Karan S. Saini,[1] Isabel Beets,[4] Petra E. Vértes,[3] William R. Schafer,[2,4] Eyal Ben-David,[5,6,*] and Oliver Hobert[1,7,*]
[1]Department of Biological Sciences, Howard Hughes Medical Institute, Columbia University, New York, NY 10027, USA
[2]Neurobiology Division, MRC Laboratory of Molecular Biology, Cambridge CB2 0QH, UK
[3]Department of Psychiatry, University of Cambridge, Cambridge CB2 0SZ, UK
[4]Department of Biology, KU of Leuven, 3000 Leuven, Belgium
[5]Department of Biochemistry and Molecular Biology, Institute for Medical Research Israel-Canada, The Hebrew University of Jerusalem, Jerusalem, Israel
[6]Present address: Illumina Artificial Intelligence Laboratory, Illumina Inc, Foster City, CA 94404, USA
[7]Lead contact
*Correspondence: it2309@columbia.edu (I.A.T.), eyal.bendavid@mail.huji.ac.il (E.B.-D.), or38@columbia.edu (O.H.)
https://doi.org/10.1016/j.cub.2025.05.036

SUMMARY

One avenue to better understand brain evolution is to map molecular patterns of evolutionary changes in neuronal cell types across entire nervous systems of distantly related species. Generating whole-animal single-cell transcriptomes of three nematode species from the *Caenorhabditis* genus, we observed a remarkable stability of neuronal-cell-type identities over more than 45 million years of evolution. Conserved patterns of combinatorial expression of homeodomain transcription factors are among the best classifiers of homologous neuron classes. Unexpectedly, we discover an extensive divergence in neuronal signaling pathways. Although identities of neurotransmitter-producing neurons (glutamate, acetylcholine, γ-aminobutyric acid [GABA], and several monoamines) remain stable, expression of ionotropic and metabotropic receptors for all these neurotransmitter systems shows substantial divergence, resulting in more than half of all neuron classes changing their capacity to be receptive to specific neurotransmitters. Neuropeptidergic signaling is also remarkably divergent, both at the level of neuropeptide expression and receptor expression, yet the overall dense network topology of the wireless neuropeptidergic connectome remains stable. Novel neuronal signaling pathways are suggested by our discovery of small secreted proteins that show no obvious hallmarks of conventional neuropeptides but show similar patterns of highly neuron-type-specific and highly evolvable expression profiles. In conclusion, by investigating the evolution of entire nervous systems at the resolution of single-neuron classes, we uncover patterns that may reflect basic principles governing evolutionary novelty in neuronal circuits.

INTRODUCTION

Evolutionary changes that shaped animal nervous systems played a pivotal role in their success in adapting to diverse environments. Characterizing these changes has been the goal of numerous comparative studies that enlightened our understanding of brain evolution. Classifications of neuronal cell types, a fundamental aim in neuroscience, traditionally relied mostly on anatomical features and, later, on electrophysiology and key molecular features such as neurotransmitter usage. More recently, these classifications have been further expanded and refined in various organisms through in-depth molecular characterization permitted by single-cell technologies.[1–6] When applied comparatively in different species, these techniques can reveal evolutionarily novel neuronal types that arose in a particular species or taxon, the species-specific loss of a neuron type, or the rare presence of a homologous cell type previously thought to be lacking in a species.[7–15] Molecular profiling also traces the evolutionary trajectories of neuronal cell classes or brain regions across divergent species, revealing whether neuronal features with functional similarities resulted from homology or from convergent evolution.[16–20]

In-depth analyses of homologous neuron types across species enables the characterization of diverging and conserved molecular features, with the potential to address critical open questions about brain evolution.[21] For example, do the patterns of deployment of different modes of neuronal communication (different neurotransmitter and neuropeptidergic systems, synaptic vs. extrasynaptic) diverge in their evolutionary trajectories? Is evolutionary change reflected uniformly in the entire brain or, conversely, do specific regions, circuits, or neuronal features constitute evolutionary hotspots? Shining

This is an open access article under the CC BY license (http://creativecommons.org/licenses/by/4.0/).

light on these central questions in respect of vertebrate brains presents several challenges. The sheer size of brains often necessitates the characterization of specific regions in isolation, preventing a global investigation across the entire nervous system with minimal sampling bias. Moreover, cataloging the complete collection of neuronal classes (both molecularly and anatomically) and the precise sets of regulatory factors specifying their terminal identity, a key requirement when defining homologous neurons,[22,23] is still an ongoing endeavor in complex organisms.

Although the complexity of vertebrate brains complicates the analysis of evolutionary trajectories on a whole-nervous-system level, the compact nervous system of nematodes[24] permits the identification of nervous-system-wide patterns and principles of evolutionary change through comparative analysis. Overall anatomical organization and embryonic lineaging studies suggest that the nervous systems of distinct nematodes appear similar.[24–26] However, this notion has never been rigorously tested with proper cellular and molecular resolution. We interrogate this issue here using three known hermaphroditic *Caenorhabditis* species, *C. elegans*, *C. briggsae*, and *C. tropicalis*, which are thought to have shared their last common ancestor more than 45 million years ago (Figure 1A)[28] (for comparison, humans and chimpanzees diverged less than 10 million years ago). Nematodes from the *Caenorhabditis* genus exhibit interspecies biases toward different climates and ecological niches and display a plethora of differences in behavioral patterns.[29–37] Specifically, in the wild, *C. tropicalis* specimens were found only in tropical regions, whereas *C. briggsae* and *C. elegans* are cosmopolitan, and *C. elegans* differs in its display of a strong bias toward cooler temperature and higher altitude.[36] Divergences also translate into the lab, with *C. elegans* and *C. briggsae* differing in their thermotaxis behaviors under controlled conditions.[29]

We hypothesized that using the knowledge accumulated from *C. elegans* neuronal development, anatomy, and function, we would be able to assess the extent to which cell types can be homologized across the *Caenorhabditis* genus and to characterize the evolution of gene expression signatures of an entire nervous system at the resolution of single neuronal classes. On the basis of our single-cell transcriptomic data, validated by genome-engineered fluorescent reporter alleles in all 3 nematode species, we provide an in-depth analysis of different gene families involved in neuronal fate specification and neuronal signaling. We discovered striking patterns of evolutionary stability as well as evolutionary change in homologous neuronal cell types. We assessed whether the changes are enriched in subsets of neurons or homogenously distributed in the nervous system. Finally, we present a primary characterization of a family of small, secreted proteins of unknown function that are abundantly expressed in the nervous system in a neuron-class-specific manner. Taken together, our analysis reveals striking patterns of evolutionary changes in neuronal signaling across 45 million years of evolution, distributed throughout the entire nervous system of each nematode species. Processed expression data from all species are available for interactive exploration at https://caenogen.shinyapps.io/caenogen/.

RESULTS

scRNA atlases of three *Caenorhabditis* species delineate homologous neuronal cell types with conserved expression of neuronal identity specifiers

We generated single-cell RNA sequencing (scRNA-seq) libraries from the three androdiecious *Caenorhabditis* species, *C. elegans*, *C. briggsae*, and *C. tropicalis*, at the second larval stage (L2) of development (Figure 1A). At this stage, all neuronal cells have been generated and wired up into a fully functional nervous system.[38,39] After quality filtering, we obtained 94,868 *C. elegans* cells, 91,596 *C. briggsae* cells, and 80,438 *C. tropicalis* cells for a total of 266,902 sequenced and tissue-annotated single cells (Figure 1B; Data S1A). Cell clusters were assigned to organ tissues based on examination of cluster-enriched differentially expressed genes with 1:1:1 primary sequence orthologs across the three species and comparisons with prior tissue-specific transcriptome studies in *C. elegans* (see STAR Methods and Data S1B–S1G).[40–42]

We subclustered neuronal cells and refined the annotation of neurons into molecularly distinct neuronal classes in the three species (Figure 1C). The final neuronal datasets include 59,338 neurons (ranging from 19,277 to 20,758 per species) encompassing 114/118 (*C. elegans* and *C. briggsae*) and 112/118 (*C. tropicalis*) neuronal classes. "Missing" neuronal cell classes do not stem from losses of the neuron types in either nematode species because all these classes were reliably detected using CRISPR-Cas9-engineered reporter alleles for neurotransmitter pathway genes (as described further below). Rather, their absence from the datasets is likely due to factors such as cell birth later in development (RMF and PVN),[43] delayed terminal differentiation (HSN and VC),[44] or limitations in sequencing depth (I3 and ASE neurons in *C. tropicalis*). Detailed information about sequenced cells from all neuronal groups and species appears in Data S1A.

We were able to robustly assign homology to all sequenced neuron classes based on the following arguments: first, large subsets of the most differentially expressed 1:1:1 ortholog genes were shared across species in most cell types (Figure S1; Data S1B–S1D). Second, hierarchical clustering of cell types by the expression of variable genes showed high concordance with our marker-based cell-type calls (Figure 1D). Third, manifold integration of all three scRNA sequencing (scRNA-seq) datasets based on all orthologs, using two alternative integration algorithms, corroborated our manual annotations (Figure S1). Fourth, because gene regulatory networks constrain cellular differentiation programs over evolutionary time,[23] an accepted minimal criterion for homology of cell types in cross-species transcriptomic data is that they will share a conserved set of transcription factors (TFs).[10,17,19] We expanded this concept further by specifically focusing on TFs that are proven key regulators of neuronal identity in *C. elegans* (including so-called "terminal selectors"). Such critical regulatory factors were determined for 111 out of the 118 neuronal classes through extensive genetic mutant analysis (Data S1H).[45,46] To date, the *C. elegans* neuronal "regulatory code" includes 62 transcription-factor-encoding genes acting as identity specifiers in at least one neuron class, 60 of which have 1:1:1 orthologs in our gene models. Inspection of

CellPress
OPEN ACCESS

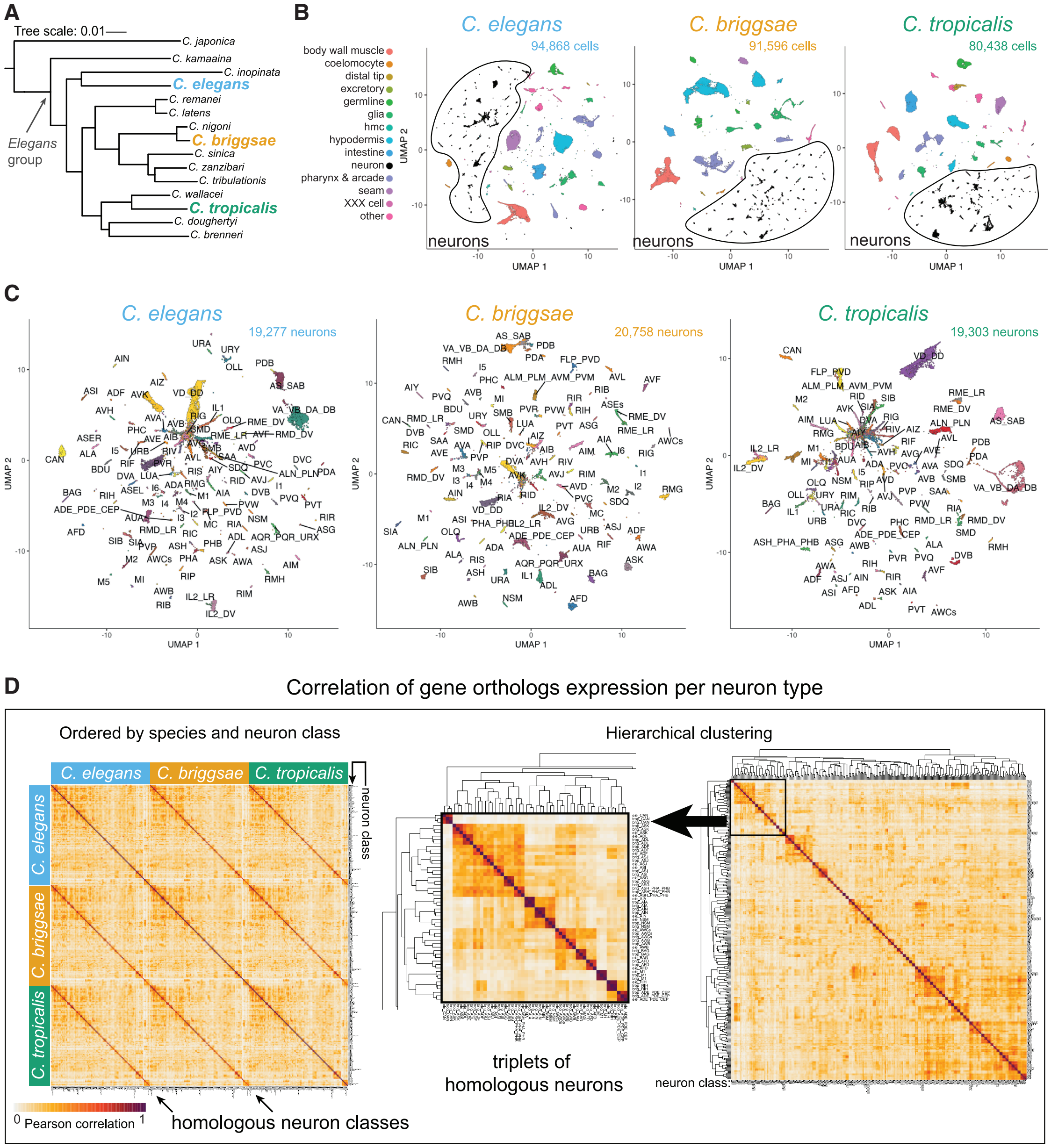

Figure 1. scRNA atlases of the entire nervous system of three *Caenorhabditis* nematode species

(A) Phylogenetic tree of species in the *Elegans* group, adapted from Stevens et al.[27] Scale: substitutions per site.

(B) UMAP representations of sequenced cells colored by tissue in 3 *Caenorhabditis* species.

(C) UMAPs of all neuronal cells. Neuron identities were determined based on 1:1:1 ortholog genes differentially expressed in *C. elegans*.

(D) Correlation heatmaps of pseudobulked normalized expression levels of 1,380 differentially expressed 1:1:1 orthologs (marker_score > 0.1 in at least one species and one neuron class). Left: rows and columns are grouped by species and then by neuron class. Right: same data, but rows and columns are arranged based on hierarchical clustering. The triplets of homologous neuron classes clustered together across species.

See also Figure S1 and Data S1.

C. briggsae and *C. tropicalis* expression data for this set of TFs showed that their cell-specific expression is overwhelmingly conserved in the homologous cell types for which they act as terminal selectors in *C. elegans* (Figures 2A and S2).

We experimentally validated the conserved expression pattern of neuronal-identity-specific TFs by using CRISPR-Cas9 to insert *gfp* in the genomic loci of two such terminal selectors' genes, the LIM homeodomain gene *lim-6* and the SIX3/6-like homeodomain gene *ceh-32*. We had previously described the expression patterns of these genes in *C. elegans*,[47] and by now tagging their orthologs in *C. briggsae* and in *C. tropicalis*, we confirmed conserved neuron-class-specific expression and cell body anatomical position using fluorescent microscopy (Figures 2B and 2C). These results strengthen

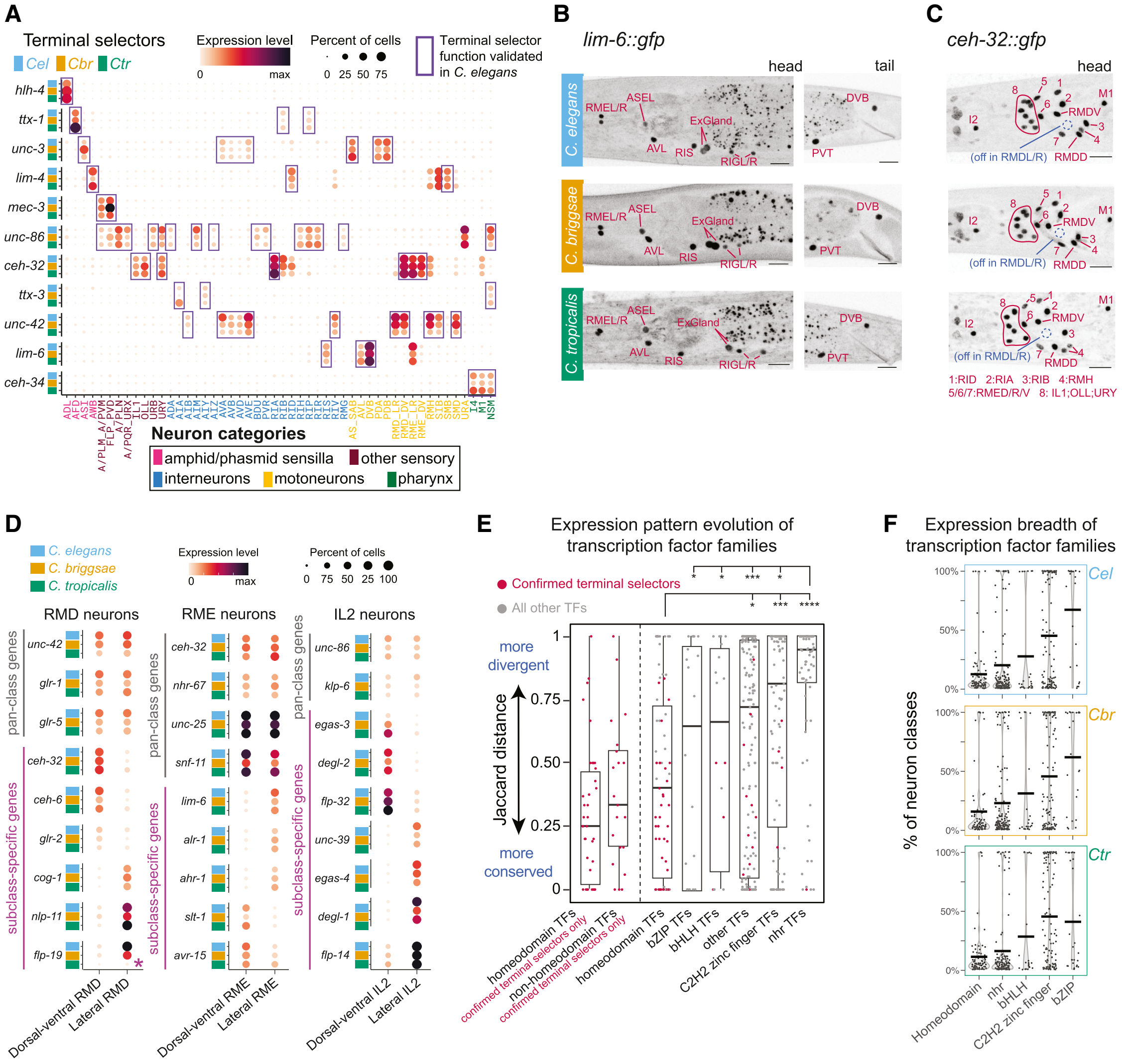

Figure 2. Stable class-specific expression of homeodomain transcription factors and neuronal identity specifiers

(A) Cross-species expression dot plot for a subset of transcription factor orthologs functionally validated as identity specifiers ("terminal selectors") in *C. elegans*. Full list of genes and neurons are shown in Figure S2. Nematode species (*y* axis) and neuron class (*x* axis) are color coded according to legend. Dot size represents the fraction of cells expressing the gene in a given neuron class, color represents scaled average expression levels.

(B and C) Representative fluorescent microscopy images (z stack max projections) of *lim-6::gfp* (B) and *ceh-32::gfp* (C) protein fusions in nematodes genomically tagged in the endogenous loci of the corresponding transcription factor. Names of expressing neuron classes are indicated in red. Scale bars: 10 μm.

(D) Cross-species expression dot plots of genes expressed in RMD-, RME-, and IL2-class neurons. Represented subclasses are labeled in the *x* axis. "Pan-class" genes (expressed in all subclasses) are marked in gray, and subclass-specific genes are marked in purple. Asterisk: expression of *flp-19*, a neuropeptide precursor gene expressed in RMD-lateral neurons, was absent specifically in *C. tropicalis*.

(E) Jaccard distances of transcription factors from different families. Each dot represents a gene. Red dots represent genes that are experimentally validated specifiers of terminal identity of at least one *C. elegans* neuron class. Boxplots are Tukey style. Left columns display separately the terminal selector genes alone (these genes thus appear twice in the plot) for ease of visualization and are not included in statistical tests. Kruskal-Wallis test, Dunn's post hoc, Benjamini-Hochberg correction for multiple comparisons. ****$p < 0.0001$, ***$p < 0.001$, *$p < 0.025$, unmarked comparisons were not significant.

(F) Expression breadth of transcription factor families. Violin plots of percentages of neuron types out of all neuron types (*y* axis) in which genes belonging to different transcription factor families (*x* axis) are expressed. Each dot represents one gene. Horizontal bars show average breadth of all genes in a given family.

See also Figures S2 and S3 and Data S1.

the neuron class homology assignments by grounding them in regulatory programs of neuronal differentiation.

Neuronal cells that share extensive phenotypic similarity and belong to the same neuron type can sometimes be further subclassified into neuronal subtypes that differ in only a small number of reproducible characteristics.[48,49] For example, the RMD-class neck motoneurons include three bilateral neuron pairs (dorsal, lateral, and ventral) that share numerous anatomical and molecular features in common, yet the dorsoventral pairs differ from the lateral pair in the expression of several terminal features, such as the glutamate AMPA receptor *glr-2* and the neuropeptides *nlp-11* and *flp-19*.[50] Our analysis of the

CellPress
OPEN ACCESS

scRNA-seq datasets captured molecularly distinct subclusters of RMD-, IL2-, and RME-class neurons across all *Caenorhabditis* species (Figure 2D). In *C. elegans* RMD, subclass-specific effector gene expression is regulated by *ceh-32*, which is expressed in dorsoventral, but not lateral, RMD neurons and acts as a specifier of RMD dorsoventral subtype identity.[50] Similarly, *lim-6* is expressed in lateral but not dorsoventral RME motoneurons and in left but not right ASE sensory neurons.[51] Using our endogenous protein fusion reporters, we found that the subtype-specific expression patterns of CEH-32 and LIM-6 are also conserved in *C. briggsae* and *C. tropicalis* (Figures 2B and 2C). Together, these findings underscore the extent to which cell-type classification is conserved across many millions of years of nematode phylogeny.

Homeodomain TF codes as classifiers of neuronal cell types

After establishing the class identity of neuronal clusters, we characterized the evolutionary dynamics of gene families that define the functional properties of neuron classes. First, for every gene and every neuron class, we sought to determine whether the detected signal from sequencing reads indicates true gene expression. To achieve this, we trained a random forest classifier on our *C. elegans* dataset and 14,406 neuron-type-specific "ground truth" observations from 147 genes that were previously validated with fluorescent markers (CRISPR-tagged endogenous genes or fosmid-based reporters, Data S1I and S1J). We then applied the newly generated classifier on all three datasets and obtained thresholded data of binary (ON/OFF) expression values. An analogous thresholding approach was successful in past *C. elegans* scRNA-seq studies and was even able to unveil true sites of expression that had been previously missed in transgenic reporter constructs limited in their *cis*-regulatory information.[40–42,52] We integrated the thresholded expression data across species and neuron types to calculate Jaccard distances for every ortholog gene, reflecting how evolutionarily divergent the gene is in its neuron-class-specific expression (Data S1I and S1J).

The Jaccard distances can be used to assess how divergent the species are relative to each other in their neuron-type-specific gene expression. *C. elegans* is phylogenetically more distant than *C. briggsae* and *C. tropicalis* are to each other (Figure 1A), but pairwise comparisons using various gene families of interest did not highlight *C. elegans* as particularly more divergent (Figure S3). In fact, the few inquiries that gave rise to significant differences highlighted *C. tropicalis* as more divergent in its neuronal transcriptome. These trends argue against the notion that neuron-type-specific expression evolved in simple correlation with evolutionary distance.

Next, we analyzed the Jaccard distance of genes across all 3 species together and focused first on different families of TFs. We found that homeobox genes were the most conserved in their neuron-type-specific expression (Figure 2E). This result is consistent with the high prevalence of homeobox genes among confirmed terminal selectors of neuronal identity (Figure S2; Data S1H). When inquiring specifically about the subset of 22 non-homeodomain TFs that are validated terminal selectors, we found that the Jaccard distances for this group were as low (conserved) as the homeodomain family. At the other extreme of the conservation spectrum, the TF family of C4 zinc finger-containing nuclear hormone receptors (nhr) stand out as particularly divergent in their neuron-type-specific expression across species. Compared with other TF families, the homeodomain TFs are the most sparsely expressed (i.e., in fewer neuron classes), yet their sparsity is sufficient to combinatorially define each individual neuron class (Figure 2F). Taken together, thanks to their conservation and their relative sparsity, combinatorial homeobox gene expression profiles represent the most robust classifiers of cell-type identity, perhaps even across metazoans.[17,53,54]

Expression of species-specific genes and differential expression of conserved orthologs are two independent modes of evolutionary novelty differentially harnessed by sensory, motor, and interneurons

Are certain neuron types evolutionarily more labile than others in their usage of the pool of genes present in the genome? To answer this question, we adopted a cell-centered approach and dissected two different modalities of evolutionary change in neuron-type-specific gene expression. The first potential modality of divergence within homologous neurons is through the gain or loss of species-specific genes, and the second is through the differential expression of homologous genes.

Initially, we established sets of species-specific genes for each nematode, defined as genes with no sequence homology to genes in either of the two other species (i.e., genes with "1-to-none" orthology relationships). We note that limiting the analysis to 1-to-none genes is too restrictive to capture all species-specific genes because the latter also include recent duplications of conserved genes, which we discuss further below. We found that the number of expressed 1-to-none species-specific genes was consistently higher (21%∼92% increase) in the amphid and phasmid sensory sensilla compared with any other neuron category in all species, both in absolute number and when normalized to the total number of expressed genes per neuron class (Figures 3A and S4). The amphid and phasmid sensilla constitute the main olfactory, chemosensory, and thermosensory organs in *C. elegans*, and their neurons expressed more species-specific genes than sensory neurons sensitive to other modalities, such as CO_2, O_2 or mechanosensation. Some additional statistically significant differences could be detected in comparisons between other functional categories of neuronal classes, but those differences were not consistent across all species and were always much smaller in their effect size (Figures 3A and S4).

To account also for recently duplicated species-specific genes, we generated separate sets of species-specific genes, which included 1-to-none genes together with "many-to-1" and "many-to-many" gene mappings. These new "permissive" sets are only an approximation of the true pool of species-specific genes because they include duplicated paralogs as well as the corresponding ancestral orthologs in all cases where the two could not be distinguished due to high sequence similarity and high synteny. Remarkably, the 1-to-none gene sets and the permissive gene sets displayed the very same evolutionary patterns of neuronal gene expression (Figures 3B and S4). We therefore deduce that these results are in high likelihood the patterns displayed by the bona fide sets of novel species-specific genes.

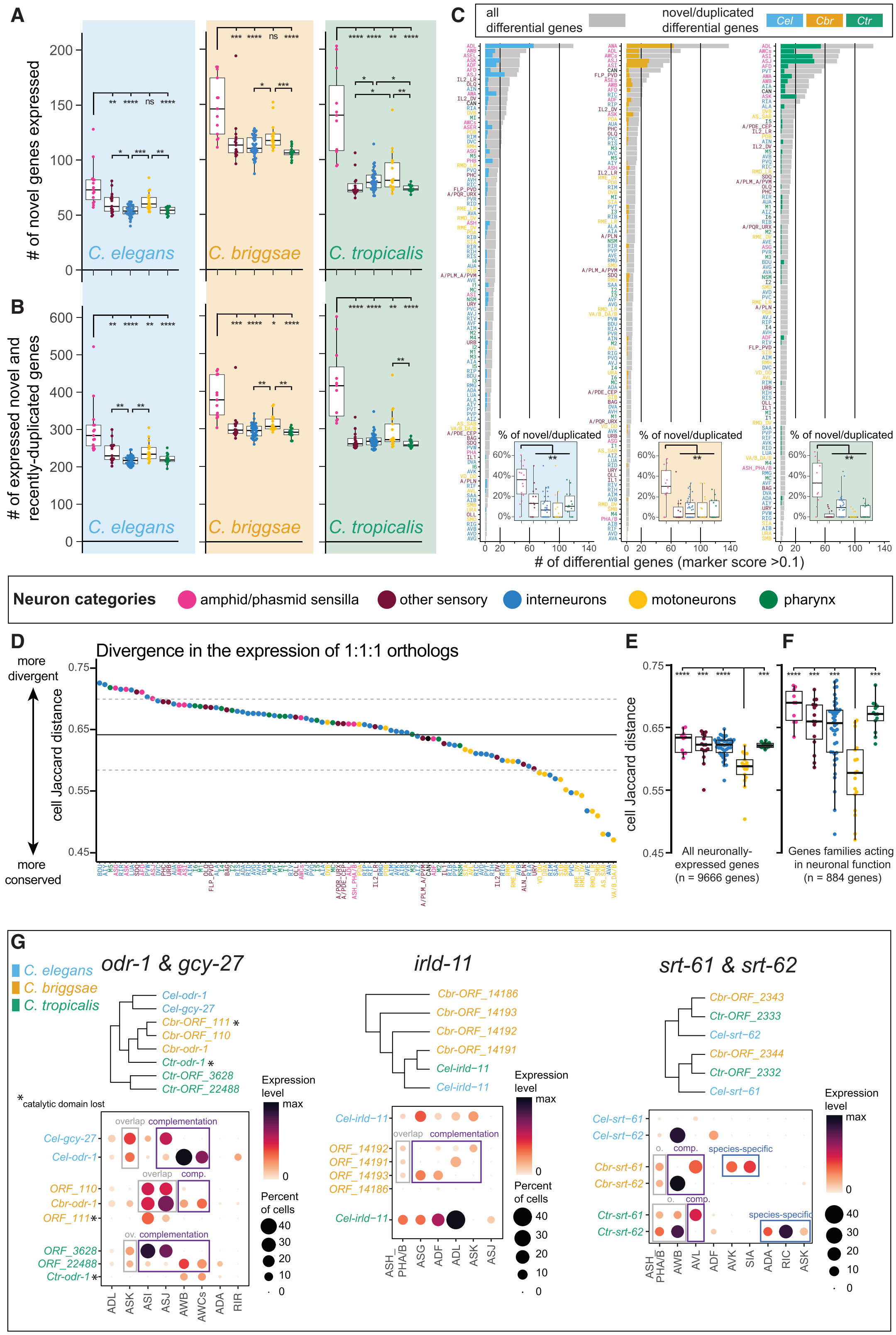

(legend on next page)

One family standing out among the sets of species-specific genes is the family of G-protein-coupled receptor (GPCR)-encoding genes. GPCR genes are abundant and diverge rapidly in the genomes of *Caenorhabditis* nematodes and of many other animal clades, including mammals.[56–59] GPCRs clearly contributed to the enrichment of novel genes in olfactory and chemosensory neurons because the vast majority of GPCRs are expressed in these neurons and because GPCRs constitute up to 19% of all novel genes expressed in the nervous system.[58] Yet, the enrichment of novel genes in sensory neurons was still evident even when GPCRs were completely excluded from the expression analyses (Figure S4), indicating that GPCRs are not the sole contributors to the observed pattern. Alongside GPCRs, other species-specific genes enriched in amphid sensilla neurons included nhr, insulin/epidermal growth factor (EGF) receptor-L domain genes (irld), and genes with no identifiable protein domains, the latter being by far the most abundant gene category among neuronally expressed species-specific genes (Figure S4).

Finally, species-specific genes were frequently among the top differentially expressed genes in amphid and phasmid sensilla neurons (Figure 3C), even when excluding GPCRs (Figure S4). Hence, they are not merely expressed at detectable levels but also tend to be abundantly and specifically expressed in those neuron classes. These findings suggest that the usage of species-specific genes is a frequent form of evolutionary novelty and a major feature of the molecular signature of chemosensory and olfactory neurons, although being less prominent in other categories of neurons.

Besides divergence in the usage of novel genes, homologous neuron types in evolving species can acquire species-specific molecular signatures through divergence in the expression of conserved, orthologous genes. To characterize this type of divergence, we calculated a Jaccard distance for each neuron class, reflecting how divergent or conserved the expression pattern is of neuronally expressed orthologous genes therein (Figure 3D). The pattern emerging from the analysis of orthologous genes differed sharply from the pattern of novel species-specific genes described above. We found that the expression of 1:1:1 orthologs was significantly more conserved in motor neurons compared with other neuron functional categories (Figures 3D–3F). Importantly, sensory neurons were not particularly divergent through this metric, displaying divergence values similar to the values of interneurons or enteric neurons. This was true when we included in the analysis either all neuronally expressed 1:1:1 orthologs (9,666 genes, Figure 3E) or a restricted pool of 884 1:1:1 orthologs belonging to gene families known to impact the functional properties of neurons (Figure 3F).[55]

In summary, our findings suggest that neuron groups are differentially impacted by two "arms" of gene expression divergences exhibiting separate evolutionary dynamics: ciliated olfactory/chemosensory neurons of the amphid and phasmid sensilla make extensive use of novel and rapidly duplicating genes, resulting in a more species-specific molecular signature compared with other categories of neurons. In parallel—and in stark contrast—neuron-class-specific expression of 1:1:1 orthologs is more evolutionarily conserved in motor neurons, whereas the levels of divergence are comparable in all remaining neuron categories, including ciliated sensory neurons. Rates of evolutionary novelty in the neuronal usage of conserved genes show no obvious "hotspot" and are slower only in motor neurons.

Subfunctionalization, neofunctionalization, overlap, and degeneration of duplicated genes at single-neuron resolution

Gene duplication is thought to play a major role in cellular innovation.[57,60,61] After duplication, redundant genes often undergo negative selection that results in degeneration and pseudogenization. In other cases, gene duplicates can diverge to adopt new functions (neofunctionalization) or to "divide labor" (subfunctionalization) by subsetting the ancestral gene function between two genetic loci now evolving independently. From a cell-type perspective, neofunctionalization and subfunctionalization can arise in a species-specific manner when gene duplicates are differentially employed by homologous cell types. Exploration of our datasets enabled the investigation of the expression patterns of paralogous expansions in the nervous system across species. Our examination unveiled several cases that involved gene families acting in sensory signaling.

Figure 3. Distribution of patterns of genomic and transcriptional novelty

(A) Number of species-specific novel genes (1-to-none orthologs) expressed in different neuron classes and species. Neuron classes were grouped into functional categories (*x* axis and color coded), each dot represents a single neuron class. Boxplots are Tukey style. Kruskal-Wallis test, Dunn's post hoc, Benjamini-Hochberg correction for multiple comparisons.

(B) Number of species-specific novel genes and recently duplicated genes (1-to-none, 1-to-many, and many-to-many orthologs) expressed in different neuron classes and species.

(C) Number of differentially expressed genes (top_markers score > 0.1, *x* axis) expressed in each neuron class (*y* axis) and species. Genes with high marker scores tend to be abundantly and specifically expressed in one or few neuron classes. Total numbers appear in gray bars, the subsets of species-specific novel genes and recently duplicated genes are colored according to the corresponding species. Bottom: proportions of novel and duplicated genes out of all differentially expressed genes per neuron class, grouped by neuron functional category. Each dot represents a neuron class.

(D) Jaccard distances (*y* axis) measuring the divergence in the usage of 1:1:1 ortholog triplets in each homologous neuron class (*x* axis) across species. Neuron classes are ordered in decreasing Jaccard distance and color coded according to functional categories. Horizontal bars: mean ± 1 SD.

(E and F) Jaccard distances (*y* axis) of neuron classes (dots) grouped into functional categories (*x* axis and colors). The calculation of Jaccard distances included (E) 9,666 genes expressed in at least one species anywhere in the nervous system or (F) 884 genes belonging to gene families with established functions in the nervous system.[55] Kruskal-Wallis test, Dunn's post hoc, Benjamini-Hochberg correction for multiple comparisons. ****$p < 0.0001$, ***$p < 0.001$, **$p < 0.01$, *$p < 0.025$.

(G) Expression patterns for orthogroups composed of closely related homologous genes. Genes are color coded according to their species. Cases of paralog overlap, complementation, and neofunctionalization are labeled in rectangles. Schematic gene phylogeny trees are shown above panels.

See also Figure S4.

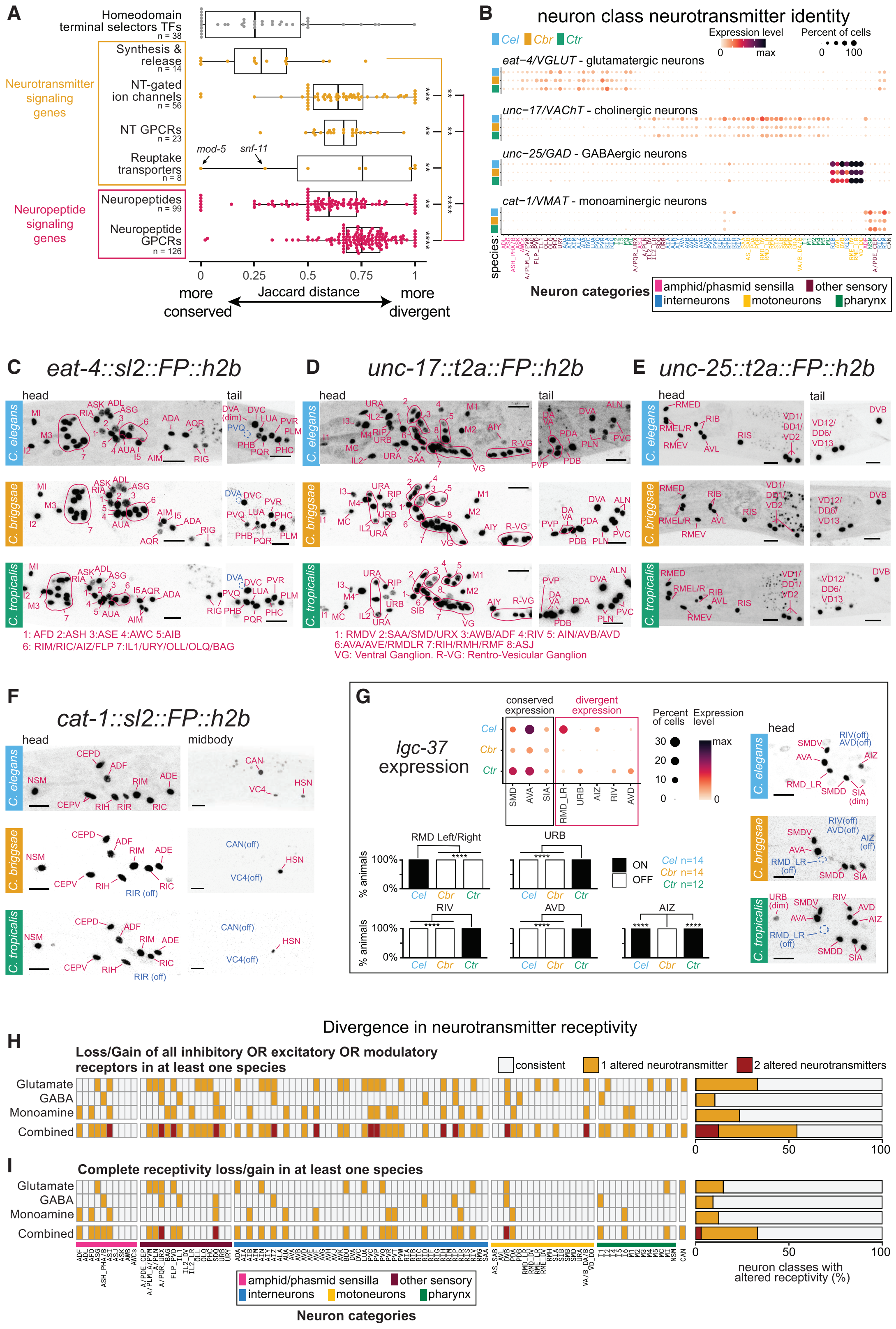

Figure 4. Divergence in cell-type-specific expression of neurotransmitter receptors amid conservation of neurotransmitter release identity
(A) Jaccard distances of genes participating in neurotransmitter signaling (yellow) and neuropeptide signaling (red). Each dot represents a gene, boxplots are Tukey style. n indicates the number of genes present in each gene group. Homeodomain transcription factor data (from Figure 2E) are shown again for

(legend continued on next page)

CellPress
OPEN ACCESS

One example is the orthogroup that includes the guanylyl cyclase genes *odr-1* and *gcy-27* in *C. elegans* and 3 paralogs in both *C. briggsae* and *C. tropicalis*. In *C. elegans*, both *odr-1* and *gcy-27* take part in olfactory and gustatory response signaling pathways.[62,63] Considering all paralogs in each species together, this orthogroup is consistently expressed in the sensory neurons ASI, ASJ, ASK, AWB, AWCs, and, to a lesser extent, ADL; however, different species display different patterns of paralog overlap and complementation (Figure 3G). In both *C. briggsae* and *C. tropicalis*, which have 3 paralogs, one of those paralogs lost its guanylate cyclase catalytic domain and is expressed at low mRNA levels in cells overlapping with another paralog, thus exhibiting a probable case of duplicate degeneration both at the level of enzymatic function and transcript expression. Moreover, the expression of all *C. briggsae* paralogs overlap in ASI and ASJ neurons, whereas *C. elegans* and *C. tropicalis* display a subfunctionalization pattern in which ASI and ASJ expression is heavily biased toward one paralog, and exrpession in AWB and AWC neurons is biased toward the second paralog. Finally, species- and class-specific novel expression of genes in this *odr-1/gcy-27* orthogroup (i.e., neofunctionalization) was detected in *C. elegans* RIR and in *C. tropicalis* ADA interneurons. Analysis of two additional orthogroups, *irld-11* (insulin/EGF receptor-L domain protein) and *srt-61/62* (GPCRs), is depicted in Figure 3G.

Evolutionary plasticity of neurotransmitter signaling pathways

Information flow in the nervous system is mediated by various chemical signals shared by all animals, such as neurotransmitters, monoamines, and neuropeptides. We used our datasets to characterize the evolutionary dynamics of the gene expression patterns of these neuronal signaling gene modules. Strikingly, we found that the expression patterns of genes involved in synthesis and vesicular loading of specific neurotransmitters (acetylcholine, γ-aminobutyric acid [GABA], and glutamate) and monoamines (dopamine, serotonin, octopamine, and tyramine) were very conserved, with conservation scores similar to cell-fate-specifying TFs (Figures 4A, 4B, and S5).

To validate this notion, we generated CRISPR-Cas9-engineered knockin fluorescent reporter alleles of *C. briggsae* and *C. tropicalis* genes that mark the different neurotransmitter systems in nematode, namely *eat-4/VGLUT* (glutamatergic neurons), *unc-17/VAchT* (cholinergic neurons), *unc-25/GAD* (GABA-synthesizing neurons), and *cat-1/VMAT* (monoaminergic neurons) and compared the expression patterns with the available complete map of neurotransmitter identity in *C. elegans* (Figures 4C–4F).[52] Overall, this reporter analysis confirms conserved neuron-class-specific expression of these four neurotransmitter identity features. We detected subtle cases of species-specific differences in dimly expressing cells, such as *eat-4* in DVA neurons (dim in *Cel*, OFF in *Cbr* and *Ctr*), *eat-4* in PVQ neurons (ON in *Cbr* and *Ctr* and OFF in *Cel*), and *cat-1* in RIR, CAN, and VC4-VC5 neurons (ON in *Cel*, OFF in *Cbr and Ctr*).

In stark contrast to genes conferring neurotransmitter identity to the *sending* neurons, we found that genes encoding for neurotransmitter *receptors* display high divergence in their neuron-class-specific expression across species (Figure 4A). This was true both for neurotransmitter-gated ion channels (n = 56 genes with 1:1:1 orthologs) and neurotransmitter metabotropic receptors (n = 23) and was independent of the neurotransmitter system (Figure S5; Data S2A and S2B). Using CRISPR-Cas9-genome-engineered reporter alleles, we validated the species-specific components of the expression patterns of the alpha-subunit-type $GABA_A$ receptor *lgc-37* in each of the three *Caenorhabditis* species (Figure 4G). In aggregate, we found that 54% of all neuron classes throughout the entire nervous system lost or gained at least one type of neurotransmitter receptivity, defined by the loss/gain of all excitatory, all inhibitory, or all modulatory receptors for a given neurotransmitter group (glutamate, GABA, or monoamines) in one species but not the others (Figures 4H and S5). Remarkably, 33% of all neuron classes

visualization purposes. The neurotransmitter reuptake transporters *mod-5* (serotonin reuptake) and *snf-11* (GABA reuptake), the sole transporters that are essential for conferring neurotransmitter identity in some neurons, were the most conserved reuptakers. Kruskal-Wallis test, Dunn's post hoc, Benjamini-Hochberg correction for multiple comparisons. ****p < 0.0001, ***p < 0.001, **p < 0.01.

(B) Cross-species expression dot plot of neurotransmitter synthesis and vesicular transporter genes determining neurotransmitter release identity of neuronal cell classes. Nematode species (y axis) and neuron class (x axis) are color coded according to legend. Neuron classes negative for all 4 genes were excluded. Dot size represents the fraction of cells expressing the gene in a given neuron class, color represents scaled average expression levels.

(C–F) Representative fluorescent microscopy images (z stack max projections, scale bars: 10 μm) of strains with reporter alleles tagging expression of *eat-4/VGLUT* (C), *unc-17/VAChT* (D), *unc-25/GAD* (E) and *cat-1/VMAT* (F). Names of expressing neuron classes are indicated in red. VG, ventral ganglion; R-VG, retrovesicular ganglion. Out-of-frame cells with stable signal across species include ALM;AVM;PVD (*eat-4*+), HSN;SDQ;PVN and cholinergic ventral nerve cord motor neurons (*unc-17*+), VD;DD neurons (*unc-25*+), PDE (*cat-1*+). Detectable differences include *eat-4* in DVA neurons (dim in *Cel*, OFF in *Cbr* and *Ctr*), *eat-4* in PVQ neurons (ON in *Cbr* and *Ctr* and OFF in *Cel*), *cat-1* in RIR, CAN, VC4-VC5 neurons (ON in *Cel*, OFF in *Cbr* and *Ctr*).

(G) Representative images and quantifications of neuron-class-specific expression of the GABA-gated ion channel gene *lgc-37* in head neurons (z stack max projections, scale bars: 10 μm). Sites of expression and divergence are labeled. Corresponding scRNA-seq expression data are shown in dot plots. Bar plots represent proportions of scored animals expressing *lgc-37* in the indicated neuron class. Fisher's test with Bonferroni correction to multiple comparisons. ****p < 0.0001. The *C. briggsae* and *C. tropicalis* strains contain a CRISPR-inserted ::t2a::mScarlet3::h2b endogenous reporter allele. For the *C. elegans* strain, a fosmid-based construct was used. Cell IDs for RIV, AVD, and AIZ were based on cell position and sequencing data, RMD_LR was confirmed using a srlf-7::gfp array.

(H) Heatmap representing neuron classes in which expression of receptors for the specified neurotransmitter ligand (y axis) were consistent in all 3 species (gray) or altered (colored). Expression was considered altered if a neuron class expresses ≥2 receptors of a certain type in at least one species and 0 receptors in another species. Inhibitory, excitatory, and modulatory receptors for a same ligand were considered separately. Cumulative data are represented in bar plots on the right. Full data in Figure S5, information about included receptors in Data S2A. Several acetylcholine receptors were broadly expressed throughout the nervous system, which meant the strict criteria for receptor divergence was not met by cholinergic receptors.

(I) As in (H) but all receptors within a neurotransmitter system were considered together. Therefore, a colored neuron class indicates a complete loss/gain of receptivity to the neurotransmitter in one of the nematodes.

See also Figure S5 and Data S2.

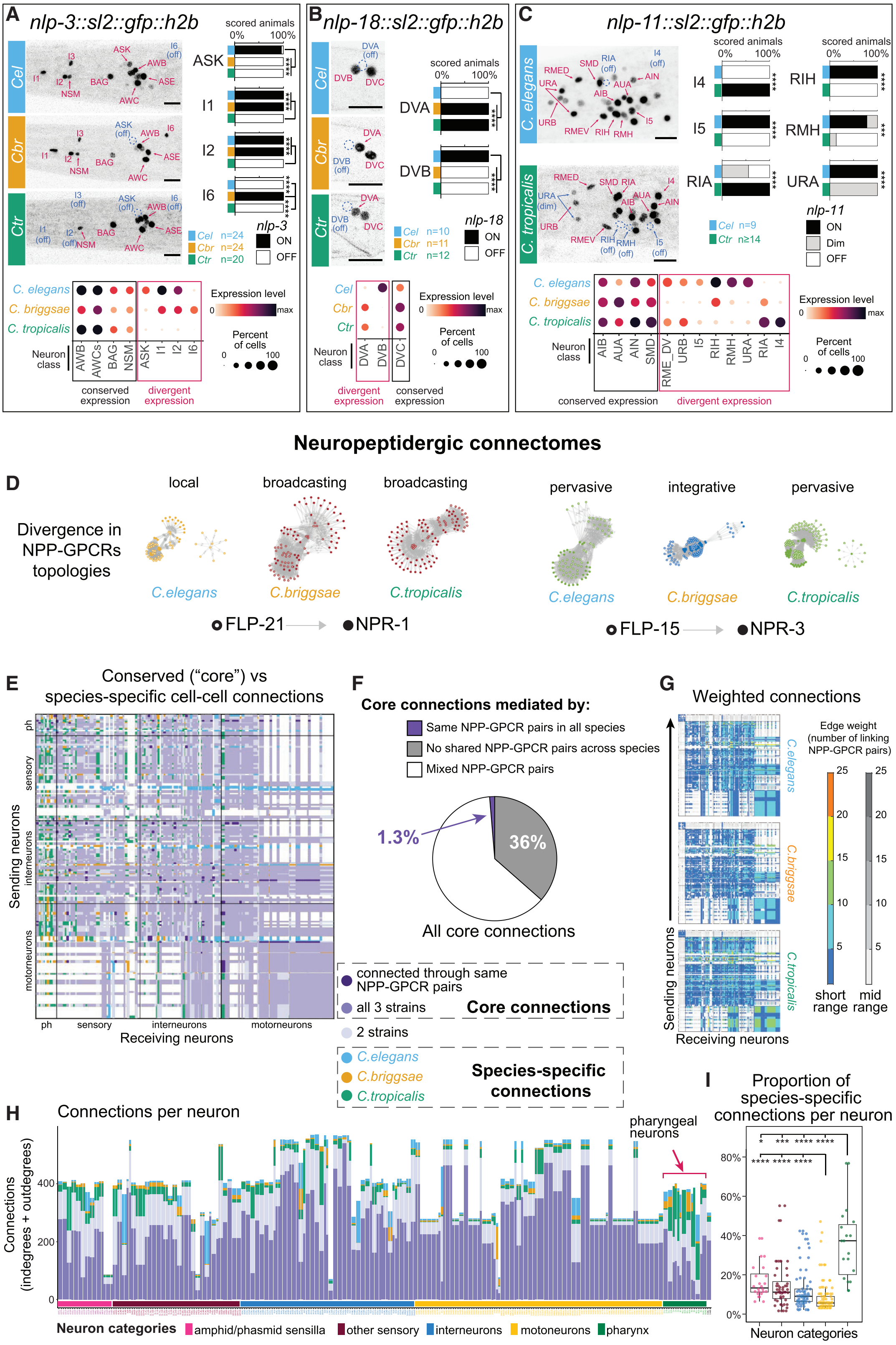

Figure 5. Evolutionary plasticity of neuropeptidergic signaling networks

(A–C) Representative images and quantifications of neuron-class-specific expression of neuropeptide precursor genes *nlp-3* (A), *nlp-18* (B) and *nlp-11* (C) tagged with *sl2::gfp::h2b* fluorescent reporter alleles in their endogenous genomic loci (z stack max projections, scale bars: 10 μm). Sites of expression and divergence

(legend continued on next page)

lost all their known receptors to a given neurotransmitter, becoming selectively "neurotransmitter-deaf" in at least one species (Figures 4I and S5). This occurred in 14 neuron types for glutamate receptivity, in 9 neuron types for GABA receptivity, and in 12 neuron types for receptivity to all monoamines. Loss of receptivity to a given neurotransmitter does not render the neuron non-excitable, as in nearly all cases of loss of receptivity to one neurotransmitter system, the neurons retain their expression of receptors for alternative neurotransmitter systems (Figure S5; Data S2C).

Taken together, our results indicate a strong selective pressure on cells to retain stable neurotransmitter identity on the releasing side, while displaying rapid divergence as receivers of neurotransmitter-mediated signals.

Evolutionary plasticity of neuropeptidergic signaling networks

Neuropeptidergic signaling is deeply conserved in all animal nervous systems and carries critical functions in neural activity and animal behavior.[64–67] Apart from using one or two classes of neurotransmitters, each *C. elegans* neuron expresses a multitude of neuropeptide-encoding genes, as well as neuropeptide receptors, generating dense "wireless" signaling networks throughout the entire nematode nervous system.[40,68–70] Our analysis revealed significant divergence in neuropeptide signaling pathways across *Caenorhabditis* species (Figure 4A, $p < 0.01$). In contrast to the trend observed in neurotransmitter signaling genes, this divergence was evident on both the signaling and receiving ends. Neuropeptide-receptor genes were more divergent in their neuron-type-specific expression than neuropeptide genes (Figure 4A, $p < 0.0001$). We confirmed cases of neuron-type-specific divergence across species with genome-inserted fluorescent reporter alleles expressed in the endogenous regulatory context of the neuropeptide precursor genes. For example, we found that the neuropeptide *nlp-3* (Figure 5A), which is consistently expressed in neurons AWB, AWC, BAG, and NSM, displays divergent expression in ASK (ON only in *C. elegans*), I6 (ON only in *C. briggsae*), and I1, I2, and I3 (OFF only in *C. tropicalis*). Likewise, our endogenous *nlp-18* reporter alleles were expressed in *C. elegans* DVB but not DVA, and vice versa in *C. briggsae* and *C. tropicalis* (Figure 5B), and *nlp-11* exhibited divergence in expression in a multitude of neuron classes (Figure 5C).

Next, we assembled "wireless connectomes" to dissect the evolutionary plasticity of neuropeptidergic signal flow in the nervous system (Data S2D–S2O).[68,70] We included biochemically validated neuropeptide/receptor pairs, with 1:1:1 orthologs in all three species (42 neuropeptide precursor genes [NPP] and 47 peptide GPCR genes, forming 84 unique ligand-receptor pairs). Analysis of their expression patterns revealed a notable evolutionary divergence of network topologies. Network topologies can be classified into local, integrative, broadcasting, or pervasive signaling based on the number of neuron types in which the NPP and the GPCR of a designated couple are expressed (Figures 5D and S6). In 19 (23%) NPP-GPCR couples included in the analysis, cell-type-specific changes in gene expression resulted in a topology difference between species (Figure S6). For example, expression patterns of the neuropeptide precursor *flp-21* and its biochemically confirmed receptor *npr-1* form a "local" network in *C. elegans* because both genes are expressed in a limited number of cells, whereas in *C. briggsae* and *C. tropicalis*, this pair forms a broadcasting network due to extended expression breadth of the receptor *npr-1* (Figure 5D). In another example, the networks formed by *flp-15* and *npr-3* display an integrative topology (many senders and few receivers) in *C. briggsae* but pervasive topologies (many senders and receivers) in *C. elegans* and *C. tropicalis* (Figure 5D).

On a global level, the neuropeptidergic networks of all three species are highly connected (Figures 5E–5G). Between 70% and 84% (depending on the inquired species and the network range) of cell-cell wireless connections found in one species were conserved across all three species (Figure 5E; "core connectome"). Yet, intriguingly, the molecular entities that assemble the core wireless connectome (i.e., the particular neuropeptides and GPCR-encoding genes) display widespread drift between species. Indeed, only ~1.2% (336/28,351 short range and 476/35,791 mid range) of the connections in the core connectome are formed via the same sets of NPP-GPCR pairs, and 36% of the core connections share no common NPP-GPCR orthologous pair across the three species (Figures 5E and 5F). These findings suggest that the global structure of neuropeptidergic signaling

are labeled on the fluorescent microscopy images. Bar plots represent proportions of scored animals expressing the corresponding gene in the indicated neuron class. Numbers of scored animals (*n*) appear below the bar plots. Fisher's test with Bonferroni correction to multiple comparisons. ****$p < 0.0001$, ***$p < 0.001$. Corresponding scRNA-seq expression data are shown in dot plots (bottom).

(D) Network topologies of the indicated pairs of neuropeptide precursor genes and GPCRs (NPP/GPCR pairs). Empty circles represent neuropeptide expression, filled circles represent receptor expression. Local networks display restricted NPP and GPCR expression (≤50 neurons), pervasive display broad NPP and GPCR expression (>50 neurons), broadcaster networks display restricted NPP but broad GPCR expression, integrative networks display broad NPP but restricted GPCR expression.

(E) Thresholded neuropeptidergic connectome (mid range) showing the evolutionary pattern of connections between sending neurons (*y* axis) and receiving neurons (*x* axis). Core (conserved) connections and species-specific connections are color coded. To be included, neuropeptide-receptor couples (in their *C. elegans* version) had to pass the functionally validated threshold of EC_{50} < 500nM binding *in vitro*.[68,70]

(F) Analysis of the subset of conserved connections producing the core connectome. 1.3% of connections are formed by the same sets of NPP-GPCR pairs across the three species. 36% of connections share no common NPP-GPCR across the three species. The remaining connections share some, but not all, NPP-GPCR pairs in common.

(G) Weighted neuropeptidergic connectomes, indicating for every cell-cell connection how many pairs of neuropeptide receptors mediate the connections.

(H) Total number of degrees (*y* axis, mid-range networks) in homologous neurons across species (*x* axis) classified by functional categories. Bars are color-filled according to the subsets of core degrees and species-specific degrees of the neuron across species.

(I) Proportions of species-specific degrees (*y* axis) per neuron classified by functional categories (colors and *x* axis). Kruskal-Wallis test, Dunn's post hoc, Benjamini-Hochberg correction for multiple comparisons, ****$p < 0.0001$, ***$p < 0.001$, *$p < 0.025$.

See also Figures S6 and S7 and Data S2.

networks remain stable in spite of extensive divergence in the expression patterns of the individual genes establishing the networks.

Further support for this notion comes from our analysis of the peptidergic degrees of different neuron classes across the nervous system. Peptidergic degree is defined as the number of incoming and outgoing connections per neuron. Our results indicate very strong cross-species correlations (r = 0.87~0.9) of degrees between homologous neuron classes (Figure S7). Accordingly, densely connected "peptidergic hubs[40,68–70]" were maintained across species, as well as the general structure of the distributions of degrees in neuronal types (Figure S7; Data S2D–S2O). This means that despite the pronounced evolutionary divergence in cell-specific expression of neuropeptide and receptor genes, homologous cells tend to retain similar breadths of connectivity, both as emitting and receiving cells.

Alongside the core connectome, each of the species also displays species-specific cell-cell neuropeptidergic connections (Figure 5E), constituting 5% of all connections in *C. elegans*, 2% in *C. briggsae*, and 8% in *C. tropicalis*. Close examination of these species-specific edges revealed that they were disproportionally more frequent among cell-cell connections to and from the enteric neurons of the pharynx (Figures 5E, 5H, and 5I; $p < 10^{-9}$ hypergeometric test). The enteric nervous system of the pharynx is synaptically connected to the rest of the central nervous system through a single neuron pair (RIP) but has previously been shown to extensively communicate with the central nervous system via wireless peptidergic connections.[68,70] The species-specificity of both incoming and outgoing connections to the enteric nervous system were proportionally 2.8×~6× more abundant in pharyngeal neurons than in all other categories of neurons (Figures 5H, 5I, and S7). We ran additional analyses focusing specifically on the interorgan communication between the central and enteric nervous systems in search for conserved themes beyond the abundant differences between species (Figure S7; Data S2P–S2Q). We found that the oxygen-sensing neurons URX/AQR were the most prominent senders and receivers of interorgan signaling, and nociceptive neurons ASH and interneurons PVR and PVT were prominent senders (although all these neurons are also hubs within the central nervous system). From the perspective of the enteric nervous system, in all species, I6 and M2 were among the top 3 heaviest senders and I1 MI and NSM among the top 5 receivers. Together, our findings extend our understanding of peptidergic signaling operating between the enteric and somatic nervous systems and illustrate that this type of interorgan crosstalk is more evolvable than other pathways of communication.

High transcriptomic evolutionary plasticity of orphan GPCRs and innexin genes

We probed the possible existence and evolutionary plasticity of previously unexplored neuronal signaling pathways by considering the large family of GPCR-encoding genes (1,596 in *C. elegans*, 761 in *C. briggsae*, and 1,042 in *C. tropicalis*). We substracted from these lists GPCRs that are either (1) sequence homologs to known GPCR-type neurotransmitter receptors and neuropeptide receptors; or (2) are exclusively expressed in sensory neurons and, hence, likely chemosensory receptors for external cues; or (3) are not robustly expressed in any neuronal cell type (See STAR Methods). This left 51 *C. elegans*, 63 *C. briggsae*, and 69 *C. tropicalis* GPCR-encoding genes, all of which are candidate receptors for internal signaling molecules (Figure 6A and S8; Data S3A and S3B). Of those genes, we detected neuron-type specificity (arbitrarily defined here as expression in <20 of neuron classes) in 50/51 (98%) *C. elegans* GPCRs, 57/63 (90%) in *C. briggsae*, and 67/69 (97%) in *C. tropicalis*. This apparent specificity in gene expression indicates a possible role in neuron-type-specific modulation of information flow. Neurons that expressed the highest number of such GPCRs in all species include the neuropeptidergic hubs PVQ and PVT,[68] the octopaminergic class RIC, as well as AIN, AIM, RIP, I5, and CAN (Figure S8; Data S3B). The presence of CAN among the top-ranked neurons in this list is notable because CAN neurons lack known chemical synapses with other neurons but potentially engage in non-synaptic signaling.[52,68] A total of 38 genes are conserved 1:1:1 orthologs and their expression in non-sensory neurons in at least one species leads us to suggest that these receptors may respond to internal signals. Based on Jaccard distance, the expression patterns of these 1:1:1 orthologs are divergent, even more so than the divergence of the neurotransmitter and neuropeptide GPCRs (Figure 6B).

Frequent cell-type-specific divergence in expression was also displayed by the family of innexins, the invertebrate constituents of electrical synapses (Figure 6B). For example, the expression of *inx-20* in ADL neurons, where it is implicated in the response to noxious chemical stimuli,[71] is specific to *C. elegans* and is not detected in *C. briggsae* nor *C. tropicalis* (Figure S9). The evolutionary plasticity in the innexin expression code extends earlier findings of development- and environment-dependent plasticity of their expression.[72] Conversely, some functionally significant sites of expression that were characterized in previous *C. elegans* studies are clearly conserved across species, such as *inx-1* in AIB, *unc-7* and *inx-19* in AVB, or *unc-9* in motor neurons of the ventral nerve cord.

A novel gene family of NSSPs

While examining the sets of the most differentially expressed genes in individual neuron types, we often noticed unknown conserved genes predicted to encode for small secreted proteins. We defined "neuronal small secreted proteins" (from here on referred to as "NSSPs") in each nematode species as being (1) shorter than 200 amino acids (>95% of known neuropeptide precursor proteins in *C. elegans* are shorter than this cutoff, Figure S10), (2) showing a signal peptide, (3) lacking a transmembrane domain and predicted enzymatic domains, and (4) displaying specific gene expression features (cell-type specificity and mRNA abundance) (Figures 7A and S10; Data S3C–S3E). These criteria were fulfilled by 90 (*C. elegans*), 84 (*C. briggsae*), and 88 (*C. tropicalis*) NSSPs (Figures 7A, 7B, S10, and S11; Data S3C and S3D). Even though the filtering pipeline was implemented separately for each of the 3 species, 47 genes (52%~56%) in the final lists were 1:1:1 orthologs that fulfill all NSSP criteria in all 3 species. The proportions rise to 71%–82% of NSSP genes of one species being present in the final list of at least one additional species (Data S3C and S3D). None of these genes were previously described or characterized. We expressed 5 conserved NSSPs fused to tagRFP in motor neurons of the ventral nerve cord. We observed protein

CellPress
OPEN ACCESS

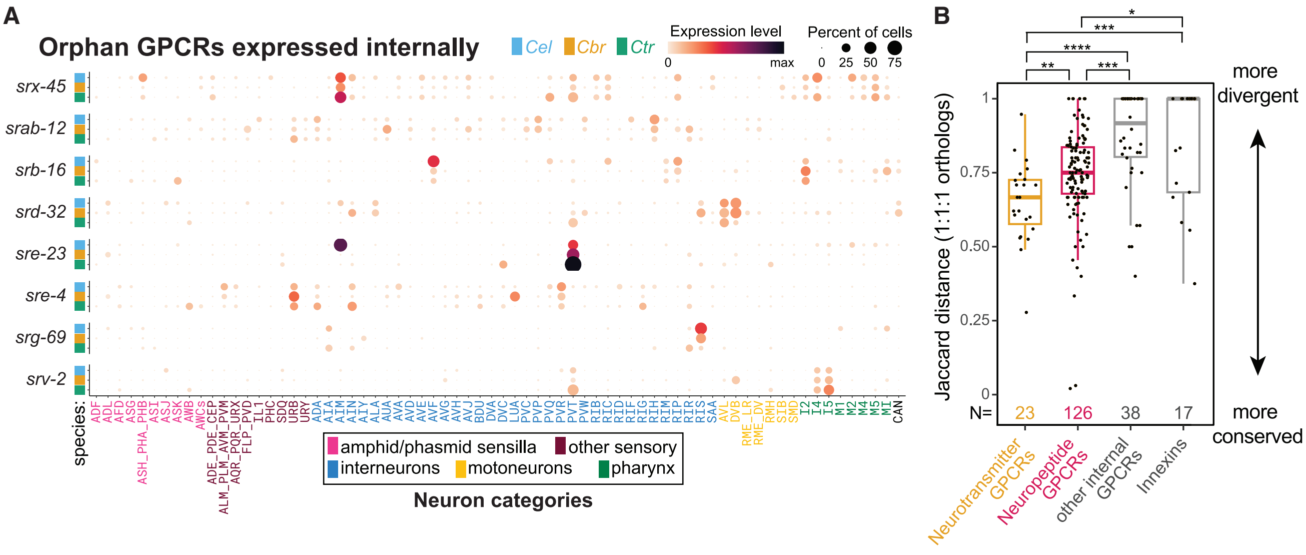

Figure 6. Evolutionary plasticity of orphan GPCRs and innexins

(A) Cross-species expression dot plot of a subset of orphan GPCRs that are expressed in non-sensory neurons and have no sequence homology with neuropeptide- and -neurotransmitter-binding GPCRs. Nematode species (*y* axis) and neuron class (*x* axis) are color coded according to legend. Dot size represents the fraction of cells expressing the gene in a given neuron class, color represents scaled average expression levels.

(B) Jaccard distances of 1:1:1 GPCR orthologs grouped according to their known ligand and innexin orthologs. Each dot represents a gene, boxplots are Tukey style. N indicates the number of genes per group, only genes expressed anywhere in the nervous system were included. Kruskal-Wallis test, Dunn's post hoc, Benjamini-Hochberg correction for multiple comparisons. ****$p < 0.0001$, ***$p < 0.001$, **$p < 0.01$, *$p < 0.025$.

See also Figures S8 and S9 and Data S3.

uptake by scavenger-like coelomocytes (5/5) and punctate localization at presumptive axonal release sites (4/5), both features of secretion that are displayed by canonical neuropeptides (Figure 7C).[73–75] Of note, our analysis also uncovered sets of uncharacterized small secreted proteins abundantly and specifically expressed in glia (Figure S11; Data S3F), which are known to signal to neuronal and non-neuronal tissue.[76–78] Each set comprised between 11 and 20 genes, including 8 genes that are 1:1:1 orthologs found specifically in sheath-type glia across all species.

Given the way in which we filtered and delineated the pools of NSSPs, it is unsurprising that these genes share with neuropeptide-encoding genes the feature of highly abundant expression levels in the nervous system. Unexpectedly, however, we found that NSSPs and neuropeptides shared additional expression and evolutionary features in common. Neuron-class-specific expression of neuropeptide and NSSP 1:1:1 ortholog genes exhibited similar rates of evolutionary divergence, as reflected in their Jaccard distances (Figure 7D). Genes from both groups also tend to be sparsely expressed in a limited number of neuronal cell classes, which, in aggregate, span the entire nervous system (Figures 7B, 7E, and S11). Like neuropeptides, NSSPs are overrepresented among the most abundantly and class-specifically expressed genes in the nervous system (Figures 7F and S11), making them a key distinctive molecular feature of a majority of neuron classes. Perhaps most remarkably, like neuropeptides, each neuron class expressed a unique combination of multiple NSSP genes (averaging 14 genes).

In conclusion, NSSPs may define previously unknown signaling axes in nematode nervous systems. This possibility is especially compelling given the ever-growing body of evidence that highlights the prominent role of non-synaptic peptidergic signaling in circuit function and complex behaviors, including in mammals.[67,79–83]

DISCUSSION

We have leveraged here the compact nature of nematode nervous systems and their well-annotated genomes to reveal patterns of evolutionary changes throughout the entire brains of nematodes that diverged >40 millions of years ago. We found no evidence for the evolution of novel neuronal cell types. Rather, each individual neuron class could be readily homologized by either considering the entire battery of genes expressed in a neuron type or, more succinctly, by the combination of homeodomain TFs. The latter is of utmost importance for homology assignment because gene regulatory networks are viewed as the ultimate constraint on differentiation programs over evolutionary timescales.[22,23] It has been previously recognized that TFs provide a key proxy for cell-type classification.[10,16,17,19] Our deep knowledge of TF function in *C. elegans*, developed over several decades of genetic loss-of-function studies,[45] reveals that not all of the many TFs that are expressed in a mature neuron class carry similar weight for proper neuronal classification. It has rather become clear—and is further validated here in the context of additional species—that of all TF families, homeobox genes represent the most potent classifiers of neuronal identity.[47] This insight may help to classify neuronal cell types in other organisms, particularly in those with limited available data on TF function.

Aside from the apparent stability of at least specific subsets of TFs, our analysis describes a wide range of molecular changes in nematode nervous systems. The nervous-system-wide nature

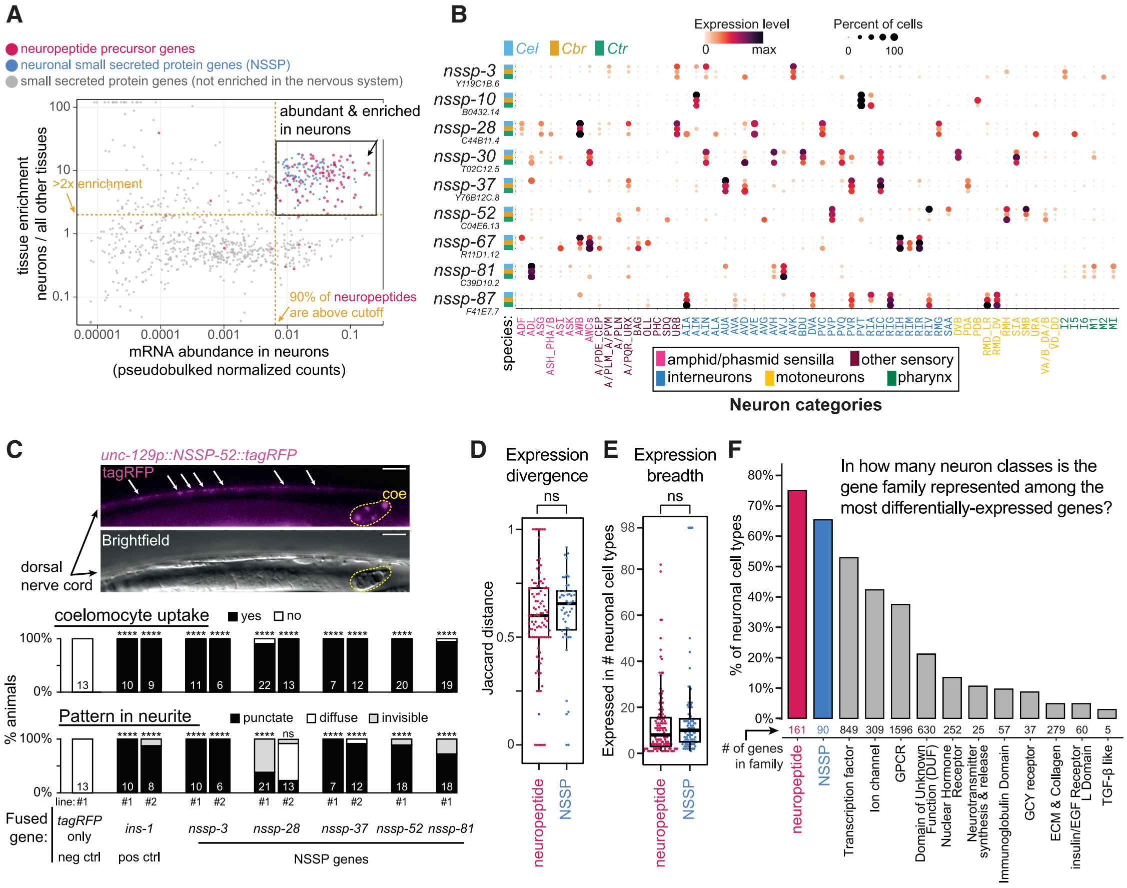

Figure 7. Characterization of neuronal small secreted proteins (NSSPs)

(A) Neuronal enrichment (*y* axis) and neuronal expression levels (*x* axis) of genes encoding small secreted proteins in *C. elegans*. Each dot is a gene, neuropeptides (red) and the novel family of NSSPs (blue) are colored. Dashed lines: cutoff criteria used to delineate NSSPs.

(B) Cross-species expression dot plot of a subset of conserved 1:1:1 NSSP orthologs. NSSPs tend to be expressed at high levels in few neuron classes. Nematode species (*y* axis) and neuron class (*x* axis) are color coded according to legend. Dot size represents the fraction of cells expressing the gene in a given neuron class, color represents scaled average expression levels.

(C) Expression of NSSP::tagRFP protein fusion transgenes in cholinergic motor neurons (unc-129 promoter). Secreted proteins are taken up by coelomocytes, resulting in detectable fluorescence therein. Localization in axonal release vesicles can be observed by punctate expression in the dorsal nerve cord neurites. Top: representative images of unc-129p::C04E6.13::tagRFP fluorescence pattern (magenta). Coelomocyte is marked in yellow. Arrows mark punctate fluorescence in the nerve cord. Scale bar: 10 μm. Bottom: quantifications of coelomocytes uptake and expression pattern in nerve cord (percentage out of all tested animals, *y* axis). Fused protein shown in *x* axis. unc-129p::tagRFP with no fused protein was used as negative control. unc-129p::INS-1::tagRFP was used as positive control. *n* of total tested animals shown inside bar plots. Fisher's exact test with pairwise comparisons to negative control and Bonferroni correction, **$p < 0.01$, ***$p < 0.001$, ****$p < 0.0001$.

(D and E) Jaccard distances (D) and *C. elegans* expression breadth (E) of 1:1:1 neuropeptides and NSSPs ortholog genes. Wilcoxon rank-sum test, $p > 0.05$.

(F) Proportions (*y* axis) of neuron classes that include genes from indicated gene family (*x* axis) among their most differentially expressed genes (top_markers score > 0.1) in *C. elegans*. Number of all genes belonging to each gene family (not only the differentially expressed genes) appears below bars.

See also Figures S10 and S11 and Data S3.

of our analysis allowed us to ask whether specific parts of the nervous system evolve more rapidly than others. Accumulating evidence from diverse phyla suggested that the sensory apparatus is more evolutionarily labile than other neuronal features, a reflection of successful adaptations to varying ecological and behavioral niches with different sensory requirements.[21,58,84–88] We found that sensory neurons indeed diverge more rapidly in the expression of non-conserved genes. Interestingly, these changes are not solely the result of gain or loss of sensory receptor genes but encompass many genes with no known protein domains. However, compared to interneurons, sensory neurons do not stand out in the context of evolving new expression of conserved genes. In contrast, we found that motor neurons differ from all other types of neurons in their stability of expression of orthologous genes. This could be a reflection of motor neurons innervating cells that are much more homogeneous in their cell typology and molecular signature (muscle cells) than the more diverse targets of sensory and interneurons (other types of neurons).

Our analysis of neuronal signaling pathways in the nervous system has revealed striking patterns of evolutionary divergence. Although the deployment of a given classic neurotransmitter system is remarkably stable over evolutionary time and

CellPress
OPEN ACCESS

neurotransmitter type (ACh, GABA, Glu, and monoamines), the patterns of reception of such signals display dramatic changes, as inferred by highly plastic expression patterns of all types of neurotransmitter receptors. These changes are best illustrated in the broadcasting network structure of monoaminergic signals,[89] where a very small number of either serotonergic, dopaminergic, tyraminergic, or octopaminergic neurons, respectively, retain their signaling capacity, but a distinct set of downstream neurons listens to these inputs in different nematode species. Taken together, in spite of an overall conserved neuronal architecture, signal flow through classic neurotransmitter systems is highly evolvable.

In addition to monoamines and fast-acting neurotransmitters, neurons release broad collections of neuropeptides that participate in elaborate signaling networks. Peptide secretion is common to neurons of all bilaterians and had already taken place in the secretory cells that preceded the emergence of the first neurons.[53,65] Nematode neurons express a combination of around 20 neuropeptide-encoding genes per neuron class.[40] We found that the expression of individual neuropeptide precursors and cognate receptor pairs is also rapidly evolving. However, thanks to our whole-nervous-system and single-neuron perspective, we discovered that despite widespread divergences at the level of individual genes, the vast majority of putative neuropeptidergic connections between pairs of homologous neurons were conserved across species. This observation may indicate stabilizing selection that maintains, in diverging species, adaptive context-dependent neuromodulatory states through compensatory routes of cell-cell interconnections. "Re-coding" of pathways of neuropeptidergic communication through compensatory gains and losses of neuropeptide and receptor expression is enabled by the deep repertoire of neuropeptides and neuropeptide receptors expressed by each individual neuron.[40]

Although compensatory changes make the neuropeptidergic connectome robust to changes in neuropeptidergic information flow in the central nervous system, the enteric nervous system of nematodes, located in the foregut (pharynx), stood out as a hotspot for rapid evolutionary divergence in its peptidergic connectivity. This was reflected in the disproportionately high concentration of species-specific edges connecting pharyngeal neurons with each other or connecting pharyngeal with somatic neurons, in both directions. Throughout animal phylogeny, enteric nervous systems often form self-contained and largely (but not completely) autonomous circuits.[90,91] Animal enteric nervous systems not only control proper digestive activity but are also being recognized as key sensory hubs that perceive a wide range of signals from food, ranging from nutritiousness to pathogenicity.[92,93] Species-specific differences in cellular interconnections might reflect functional adaptations to distinct ecological demands acting on the coordination between the enteric and somatic nervous systems to tune organismal responses to nutrients or pathogens.

Our comparative mining of the three nematode brain atlases also provides tantalizing hints for the existence of neuronal signaling modules that go beyond canonical neurotransmitter and neuropeptide signaling pathways. We described a multitude of GPCR-encoding genes, as well as small, secreted peptides, which show very selective patterns of expression with the brains of the three nematodes. The GPCRs may be receptors for internally secreted metabolites, for example, ascarosides,[94] or may find their ligand among those NSSPs that we described. Our observation of each individual neuron class expressing a unique combination of, on average, 14 NSSPs genes almost doubles the already very impressive repertoire of peptidergic signals emanating from neurons. NSSPs and GPCRs that are conserved across the three nematode species show patterns of divergence in their neuronal expression that are similar to those seen for neuropeptides and peptide receptors, if not even more pronounced. Whether these putative signaling systems may be drivers of behavioral changes remains to be investigated, but, in any case, their mere existence points to the presently perhaps underappreciated depth of signaling pathways within the nervous system.

RESOURCE AVAILABILITY

Lead contact

Further information and requests for resources and reagents should be directed to, and will be fulfilled by, the lead contact, Oliver Hobert (or38@columbia.edu).

Materials availability

Nematode strains generated for this study are available at the Caenorhabditis Genetics Center (CGC).

Data and code availability

- The raw sequencing data have been deposited at NCBI SRA as BioProject PRJNA851520 and are publicly available as of the date of publication.
- Annotated cell datasets (monocle3 objects) have been deposited at Zenodo as https://doi.org/10.5281/zenodo.14194525 and are publicly available as of the date of publication.
- Processed expression data can be interactively accessed online at https://caenogen.shinyapps.io/caenogen/.
- All original code has been deposited at Zenodo and is publicly available at https://doi.org/10.5281/zenodo.14205685 as of the date of publication.
- Any additional information required to reanalyze the data reported in this paper is available from the lead contact upon request.

ACKNOWLEDGMENTS

We thank Maria Antonietta Tosches, Itamar Lev, Stephanie Eder, Liesbet Temmerman, Giulio Valperga, Seth Taylor, and the colleagues in our labs for discussions about the project and feedback on the manuscript. We thank Qi Chen for her expertise in generating nematode strains in the lab. Other strains were provided by Christian Braendle, Erik Andersen, and the CGC (funded by the NIH, Office of Research Infrastructure Programs P40 OD010440). All research from the Department of Psychiatry at the University of Cambridge is made possible by the NIHR Cambridge Biomedical Research Centre and the NIHR East of England Applied Research Centre. The views expressed are those of the author(s) and not necessarily those of the NHS, the NIHR, or the Department of Health. This work was funded by the Howard Hughes Medical Institute and the NIH grants RO1 NS039996 and NIH RO1 NS100547 (to O.H.), the NIH grant K99 HG010369 and Israel Science Foundation grant 2023/20 (to E.B.-D.), the Medical Research Council grant MC-A023-5PB91 (to W.R.S.), the MQ Transforming Mental Health grant MGF17_24 (to P.E.V.), and a postdoctoral fellowship from the Evelyn Gruss Lipper Charitable Foundation (to I.A.T.).

AUTHOR CONTRIBUTIONS

I.A.T., E.B.-D., and O.H. designed research. E.B.-D. generated scRNA-seq libraries. I.A.T., E.B.-D., and L.T.G. analyzed data. I.A.T., L.T.G., and K.S.S. generated strains and performed experiments. L.R.-S. generated the neuropeptidergic connectomes. I.A.T. and A.S. developed the interactive website. I.B., P.E.V., W.R.S., E.B.-D., and O.H. supervised research. I.A.T. and O.H. wrote the manuscript, with input from all authors.

DECLARATION OF INTERESTS

E.B.-D. is an employee of Illumina Inc.

STAR★METHODS

Detailed methods are provided in the online version of this paper and include the following:

- KEY RESOURCES TABLE
- EXPERIMENTAL MODEL AND STUDY PARTICIPANT DETAILS
  - Cultivation of nematodes for scRNA-seq
- METHOD DETAILS
  - Cell dissociation for scRNA-seq
  - Genome engineering and transgenics
  - Microscope imaging and analysis
  - Genome builds and *in silico* demultiplexing
  - Identification of gene ortholog sets
  - Downstream processing and cell type annotation
  - Integrations of datasets across species
  - Classification of genes into gene families
  - Divergence of neurotransmitter receptors
  - Neuropeptidergic connectomes
  - Orphan GPCRs expressed in non-sensory neurons
  - Identification of NSSPs
- QUANTIFICATION AND STATISTICAL ANALYSIS
  - Transcriptomic correlation heatmaps
  - Thresholding & Jaccard distance calculations
  - Statistics analyses and graphing
- ADDITIONAL RESOURCES

SUPPLEMENTAL INFORMATION

Supplemental information can be found online at https://doi.org/10.1016/j.cub.2025.05.036.

Received: May 9, 2025
Revised: May 13, 2025
Accepted: May 14, 2025
Published: June 23, 2025

REFERENCES

1. Tasic, B., Menon, V., Nguyen, T.N., Kim, T.K., Jarsky, T., Yao, Z., Levi, B., Gray, L.T., Sorensen, S.A., Dolbeare, T., et al. (2016). Adult mouse cortical cell taxonomy revealed by single cell transcriptomics. Nat. Neurosci. *19*, 335–346. https://doi.org/10.1038/nn.4216.
2. Romanov, R.A., Zeisel, A., Bakker, J., Girach, F., Hellysaz, A., Tomer, R., Alpár, A., Mulder, J., Clotman, F., Keimpema, E., et al. (2017). Molecular interrogation of hypothalamic organization reveals distinct dopamine neuronal subtypes. Nat. Neurosci. *20*, 176–188. https://doi.org/10.1038/nn.4462.
3. Sebé-Pedrós, A., Saudemont, B., Chomsky, E., Plessier, F., Mailhé, M. P., Renno, J., Loe-Mie, Y., Lifshitz, A., Mukamel, Z., Schmutz, S., et al. (2018). Cnidarian Cell Type Diversity and Regulation Revealed by Whole-Organism Single-Cell RNA-Seq. Cell *173*, 1520–1534.e20. https://doi.org/10.1016/j.cell.2018.05.019.
4. Zeisel, A., Hochgerner, H., Lönnerberg, P., Johnsson, A., Memic, F., van der Zwan, J., Häring, M., Braun, E., Borm, L.E., La Manno, G., et al. (2018). Molecular Architecture of the Mouse Nervous System. Cell *174*, 999–1014.e22. https://doi.org/10.1016/j.cell.2018.06.021.
5. Allen, A.M., Neville, M.C., Birtles, S., Croset, V., Treiber, C.D., Waddell, S., and Goodwin, S.F. (2020). A single-cell transcriptomic atlas of the adult Drosophila ventral nerve cord. eLife *9*, e54074. https://doi.org/10.7554/eLife.54074.
6. Deryckere, A., Woych, J., Jaeger, E.C.B., and Tosches, M.A. (2023). Molecular Diversity of Neuron Types in the Salamander Amygdala and Implications for Amygdalar Evolution. Brain Behav. Evol. *98*, 61–75. https://doi.org/10.1159/000527899.
7. Hahn, J., Monavarfeshani, A., Qiao, M., Kao, A.H., Kölsch, Y., Kumar, A., Kunze, V.P., Rasys, A.M., Richardson, R., Wekselblatt, J.B., et al. (2023). Evolution of neuronal cell classes and types in the vertebrate retina. Nature *624*, 415–424. https://doi.org/10.1038/s41586-023-06638-9.
8. Chen, A., Sun, Y., Lei, Y., Li, C., Liao, S., Meng, J., Bai, Y., Liu, Z., Liang, Z., Zhu, Z., et al. (2023). Single-cell spatial transcriptome reveals cell-type organization in the macaque cortex. Cell *186*, 3726–3743.e24. https://doi.org/10.1016/j.cell.2023.06.009.
9. Krienen, F.M., Goldman, M., Zhang, Q., C H Del Rosario, R., Florio, M., Machold, R., Saunders, A., Levandowski, K., Zaniewski, H., Schuman, B., et al. (2020). Innovations present in the primate interneuron repertoire. Nature *586*, 262–269. https://doi.org/10.1038/s41586-020-2781-z.
10. Lamanna, F., Hervas-Sotomayor, F., Oel, A.P., Jandzik, D., Sobrido-Cameán, D., Santos-Durán, G.N., Martik, M.L., Stundl, J., Green, S.A., Brüning, T., et al. (2023). A lamprey neural cell type atlas illuminates the origins of the vertebrate brain. Nat. Ecol. Evol. *7*, 1714–1728. https://doi.org/10.1038/s41559-023-02170-1.
11. Schmitz, M.T., Sandoval, K., Chen, C.P., Mostajo-Radji, M.A., Seeley, W. W., Nowakowski, T.J., Ye, C.J., Paredes, M.F., and Pollen, A.A. (2022). The development and evolution of inhibitory neurons in primate cerebrum. Nature *603*, 871–877. https://doi.org/10.1038/s41586-022-04510-w.
12. Shafer, M.E.R., Sawh, A.N., and Schier, A.F. (2022). Gene family evolution underlies cell-type diversification in the hypothalamus of teleosts. Nat. Ecol. Evol. *6*, 63–76. https://doi.org/10.1038/s41559-021-01580-3.
13. Wei, J.R., Hao, Z.Z., Xu, C., Huang, M., Tang, L., Xu, N., Liu, R., Shen, Y., Teichmann, S.A., Miao, Z., et al. (2022). Identification of visual cortex cell types and species differences using single-cell RNA sequencing. Nat. Commun. *13*, 6902. https://doi.org/10.1038/s41467-022-34590-1.
14. Yamagata, M., Yan, W., and Sanes, J.R. (2021). A cell atlas of the chick retina based on single-cell transcriptomics. eLife *10*, e63907. https://doi.org/10.7554/eLife.63907.
15. Ye, D., Walsh, J.T., Junker, I.P., and Ding, Y. (2024). Changes in the cellular makeup of motor patterning circuits drive courtship song evolution in Drosophila. Curr. Biol. *34*, 2319–2329.e6. https://doi.org/10.1016/j.cub.2024.04.020.
16. Colquitt, B.M., Merullo, D.P., Konopka, G., Roberts, T.F., and Brainard, M.S. (2021). Cellular transcriptomics reveals evolutionary identities of songbird vocal circuits. Science *371*, eabd9704. https://doi.org/10.1126/science.abd9704.
17. Hain, D., Gallego-Flores, T., Klinkmann, M., Macias, A., Ciirdaeva, E., Arends, A., Thum, C., Tushev, G., Kretschmer, F., Tosches, M.A., et al. (2022). Molecular diversity and evolution of neuron types in the amniote brain. Science *377*, eabp8202. https://doi.org/10.1126/science.abp8202.
18. Horie, R., Hazbun, A., Chen, K., Cao, C., Levine, M., and Horie, T. (2018). Shared evolutionary origin of vertebrate neural crest and cranial placodes. Nature *560*, 228–232. https://doi.org/10.1038/s41586-018-0385-7.
19. Tosches, M.A., Yamawaki, T.M., Naumann, R.K., Jacobi, A.A., Tushev, G., and Laurent, G. (2018). Evolution of pallium, hippocampus, and cortical cell types revealed by single-cell transcriptomics in reptiles. Science *360*, 881–888. https://doi.org/10.1126/science.aar4237.
20. Woych, J., Ortega Gurrola, A., Deryckere, A., Jaeger, E.C.B., Gumnit, E., Merello, G., Gu, J., Joven Araus, A., Leigh, N.D., Yun, M., et al. (2022). Cell-type profiling in salamanders identifies innovations in vertebrate forebrain evolution. Science *377*, eabp9186. https://doi.org/10.1126/science.abp9186.
21. Roberts, R.J.V., Pop, S., and Prieto-Godino, L.L. (2022). Evolution of central neural circuits: state of the art and perspectives. Nat. Rev. Neurosci. *23*, 725–743. https://doi.org/10.1038/s41583-022-00644-y.
22. Arendt, D., Musser, J.M., Baker, C.V.H., Bergman, A., Cepko, C., Erwin, D.H., Pavlicev, M., Schlosser, G., Widder, S., Laubichler, M.D., et al.

(2016). The origin and evolution of cell types. Nat. Rev. Genet. *17*, 744–757. https://doi.org/10.1038/nrg.2016.127.

23. Arendt, D., Bertucci, P.Y., Achim, K., and Musser, J.M. (2019). Evolution of neuronal types and families. Curr. Opin. Neurobiol. *56*, 144–152. https://doi.org/10.1016/j.conb.2019.01.022.
24. Schafer, W. (2016). Nematode nervous systems. Curr. Biol. *26*, R955–R959. https://doi.org/10.1016/j.cub.2016.07.044.
25. Zhao, Z., Boyle, T.J., Bao, Z., Murray, J.I., Mericle, B., and Waterston, R. H. (2008). Comparative analysis of embryonic cell lineage between Caenorhabditis briggsae and Caenorhabditis elegans. Dev. Biol. *314*, 93–99. https://doi.org/10.1016/j.ydbio.2007.11.015.
26. Memar, N., Schiemann, S., Hennig, C., Findeis, D., Conradt, B., and Schnabel, R. (2019). Twenty million years of evolution: The embryogenesis of four Caenorhabditis species are indistinguishable despite extensive genome divergence. Dev. Biol. *447*, 182–199. https://doi.org/10.1016/j.ydbio.2018.12.022.
27. Stevens, L., Rooke, S., Falzon, L.C., Machuka, E.M., Momanyi, K., Murungi, M.K., Njoroge, S.M., Odinga, C.O., Ogendo, A., Ogola, J., et al. (2020). The Genome of Caenorhabditis bovis. Curr. Biol. *30*, 1023–1031.e4. https://doi.org/10.1016/j.cub.2020.01.074.
28. Qing, X., Zhang, Y.M., Sun, S., Ahmed, M., Lo, W.S., Bert, W., Holovachov, O., and Li, H. (2024). Phylogenomic Insights into the Evolution and Origin of Nematoda.. Published online December 31, 2024. Syst. Biol. syae073. https://doi.org/10.1093/sysbio/syae073.
29. Stegeman, G.W., de Mesquita, M.B., Ryu, W.S., and Cutter, A.D. (2013). Temperature-dependent behaviours are genetically variable in the nematode Caenorhabditis briggsae. J. Exp. Biol. *216*, 850–858. https://doi.org/10.1242/jeb.075408.
30. Félix, M.A., Jovelin, R., Ferrari, C., Han, S., Cho, Y.R., Andersen, E.C., Cutter, A.D., and Braendle, C. (2013). Species richness, distribution and genetic diversity of Caenorhabditis nematodes in a remote tropical rainforest. BMC Evol. Biol. *13*, 10. https://doi.org/10.1186/1471-2148-13-10.
31. Ebert, M.S., and Bargmann, C.I. (2024). Evolution remodels olfactory and mating-receptive behaviors in the transition from female to hermaphrodite reproduction. Curr. Biol. *34*, 969–979.e4. https://doi.org/10.1016/j.cub.2024.01.050.
32. Crombie, T.A., McKeown, R., Moya, N.D., Evans, K.S., Widmayer, S.J., LaGrassa, V., Roman, N., Tursunova, O., Zhang, G., Gibson, S.B., et al. (2024). CaeNDR, the Caenorhabditis Natural Diversity Resource. Nucleic Acids Res. *52*, D850–D858. https://doi.org/10.1093/nar/gkad887.
33. Chasnov, J.R., So, W.K., Chan, C.M., and Chow, K.L. (2007). The species, sex, and stage specificity of a Caenorhabditis sex pheromone. Proc. Natl. Acad. Sci. USA *104*, 6730–6735. https://doi.org/10.1073/pnas.0608050104.
34. Burton, N.O., Willis, A., Fisher, K., Braukmann, F., Price, J., Stevens, L., Baugh, L.R., Reinke, A., and Miska, E.A. (2021). Intergenerational adaptations to stress are evolutionarily conserved, stress-specific, and have deleterious trade-offs. eLife *10*, e73425. https://doi.org/10.7554/eLife.73425.
35. Poullet, N., Vielle, A., Gimond, C., Ferrari, C., and Braendle, C. (2015). Evolutionarily divergent thermal sensitivity of germline development and fertility in hermaphroditic Caenorhabditis nematodes. Evol. Dev. *17*, 380–397. https://doi.org/10.1111/ede.12170.
36. Crombie, T.A., Battlay, P., Tanny, R.E., Evans, K.S., Buchanan, C.M., Cook, D.E., Dilks, C.M., Stinson, L.A., Zdraljevic, S., Zhang, G., et al. (2022). Local adaptation and spatiotemporal patterns of genetic diversity revealed by repeated sampling of Caenorhabditis elegans across the Hawaiian Islands. Mol. Ecol. *31*, 2327–2347. https://doi.org/10.1111/mec.16400.
37. Garcia, L.R., LeBoeuf, B., and Koo, P. (2007). Diversity in mating behavior of hermaphroditic and male-female Caenorhabditis nematodes. Genetics *175*, 1761–1771. https://doi.org/10.1534/genetics.106.068304.
38. Witvliet, D., Mulcahy, B., Mitchell, J.K., Meirovitch, Y., Berger, D.R., Wu, Y., Liu, Y., Koh, W.X., Parvathala, R., Holmyard, D., et al. (2021). Connectomes across development reveal principles of brain maturation. Nature *596*, 257–261. https://doi.org/10.1038/s41586-021-03778-8.
39. Sulston, J.E. (1983). Neuronal cell lineages in the nematode Caenorhabditis elegans. Cold Spring Harb. Symp. Quant. Biol. *48*, 443–452. https://doi.org/10.1101/sqb.1983.048.01.049.
40. Taylor, S.R., Santpere, G., Weinreb, A., Barrett, A., Reilly, M.B., Xu, C., Varol, E., Oikonomou, P., Glenwinkel, L., McWhirter, R., et al. (2021). Molecular topography of an entire nervous system. Cell *184*, 4329–4347.e23. https://doi.org/10.1016/j.cell.2021.06.023.
41. Packer, J.S., Zhu, Q., Huynh, C., Sivaramakrishnan, P., Preston, E., Dueck, H., Stefanik, D., Tan, K., Trapnell, C., Kim, J., et al. (2019). A lineage-resolved molecular atlas of C. elegans embryogenesis at single-cell resolution. Science *365*, eaax1971. https://doi.org/10.1126/science.aax1971.
42. Cao, J., Packer, J.S., Ramani, V., Cusanovich, D.A., Huynh, C., Daza, R., Qiu, X., Lee, C., Furlan, S.N., Steemers, F.J., et al. (2017). Comprehensive single-cell transcriptional profiling of a multicellular organism. Science *357*, 661–667. https://doi.org/10.1126/science.aam8940.
43. Sulston, J.E., and Horvitz, H.R. (1977). Post-embryonic cell lineages of the nematode, Caenorhabditis elegans. Dev. Biol. *56*, 110–156. https://doi.org/10.1016/0012-1606(77)90158-0.
44. Pereira, L., Aeschimann, F., Wang, C., Lawson, H., Serrano-Saiz, E., Portman, D.S., Großhans, H., and Hobert, O. (2019). Timing mechanism of sexually dimorphic nervous system differentiation. eLife *8*, e42078. https://doi.org/10.7554/eLife.42078.
45. Hobert, O. (2016). A map of terminal regulators of neuronal identity in Caenorhabditis elegans. Wiley Interdiscip. Rev. Dev. Biol. *5*, 474–498. https://doi.org/10.1002/wdev.233.
46. Reilly, M.B., Tekieli, T., Cros, C., Aguilar, G.R., Lao, J., Toker, I.A., Vidal, B., Leyva-Díaz, E., Bhattacharya, A., Cook, S.J., et al. (2022). Widespread employment of conserved C. elegans homeobox genes in neuronal identity specification. PLoS Genet. *18*, e1010372. https://doi.org/10.1371/journal.pgen.1010372.
47. Reilly, M.B., Cros, C., Varol, E., Yemini, E., and Hobert, O. (2020). Unique homeobox codes delineate all the neuron classes of C. elegans. Nature *584*, 595–601. https://doi.org/10.1038/s41586-020-2618-9.
48. Hobert, O., Glenwinkel, L., and White, J. (2016). Revisiting Neuronal Cell Type Classification in Caenorhabditis elegans. Curr. Biol. *26*, R1197–R1203. https://doi.org/10.1016/j.cub.2016.10.027.
49. White, J.G., Southgate, E., Thomson, J.N., and Brenner, S. (1986). The structure of the nervous system of the nematode *Caenorhabditis elegans*. Philos. Trans. R. Soc. Lond. B Biol. Sci. *314*, 1–340. https://doi.org/10.1098/rstb.1986.0056.
50. Cros, C., and Hobert, O. (2022). Caenorhabditis elegans sine oculis/SIX-type homeobox genes act as homeotic switches to define neuronal subtype identities. Proc. Natl. Acad. Sci. USA *119*, e2206817119. https://doi.org/10.1073/pnas.2206817119.
51. Hobert, O., Tessmar, K., and Ruvkun, G. (1999). The Caenorhabditis elegans lim-6 LIM homeobox gene regulates neurite outgrowth and function of particular GABAergic neurons. Development *126*, 1547–1562. https://doi.org/10.1242/dev.126.7.1547.
52. Wang, C., Vidal, B., Sural, S., Loer, C., Aguilar, G.R., Merritt, D.M., Toker, I.A., Vogt, M.C., Cros, C.C., and Hobert, O. (2024). A neurotransmitter atlas of C. elegans males and hermaphrodites. eLife *13*, RP95402. https://doi.org/10.7554/eLife.95402.
53. Najle, S.R., Grau-Bové, X., Elek, A., Navarrete, C., Cianferoni, D., Chiva, C., Cañas-Armenteros, D., Mallabiabarrena, A., Kamm, K., Sabidó, E., et al. (2023). Stepwise emergence of the neuronal gene expression program in early animal evolution. Cell *186*, 4676–4693.e29. https://doi.org/10.1016/j.cell.2023.08.027.

54. Fung, J.H., Carriere, M., and Blumberg, A.J. (2024). Statistical estimation of sparsity and efficiency for molecular codes. bioRxiv. https://doi.org/10.1101/2024.08.13.607773.

55. Hobert, O. (2013). The neuronal genome of Caenorhabditis elegans. WormBook *2013*, 1–106. https://doi.org/10.1895/wormbook.1.161.1.

56. Thomas, J.H., and Robertson, H.M. (2008). The Caenorhabditis chemoreceptor gene families. BMC Biol. *6*, 42. https://doi.org/10.1186/1741-7007-6-42.

57. Nei, M., Niimura, Y., and Nozawa, M. (2008). The evolution of animal chemosensory receptor gene repertoires: roles of chance and necessity. Nat. Rev. Genet. *9*, 951–963. https://doi.org/10.1038/nrg2480.

58. Ma, F., Lau, C.Y., and Zheng, C. (2024). Young duplicate genes show developmental stage- and cell type-specific expression and function in Caenorhabditis elegans. Cell Genom. *4*, 100467. https://doi.org/10.1016/j.xgen.2023.100467.

59. Ma, F., Lau, C.Y., and Zheng, C. (2021). Large genetic diversity and strong positive selection in F-box and GPCR genes among the wild isolates of Caenorhabditis elegans. Genome Biol. Evol. *13*, evab048. https://doi.org/10.1093/gbe/evab048.

60. Ohno, S. (1970). Evolution by Gene Duplication (Springer). https://doi.org/10.1007/978-3-642-86659-3.

61. Force, A., Lynch, M., Pickett, F.B., Amores, A., Yan, Y.L., and Postlethwait, J. (1999). Preservation of duplicate genes by complementary, degenerative mutations. Genetics *151*, 1531–1545. https://doi.org/10.1093/genetics/151.4.1531.

62. Bargmann, C.I., Hartwieg, E., and Horvitz, H.R. (1993). Odorant-selective genes and neurons mediate olfaction in C. elegans. Cell *74*, 515–527. https://doi.org/10.1016/0092-8674(93)80053-h.

63. Krzyzanowski, M.C., Brueggemann, C., Ezak, M.J., Wood, J.F., Michaels, K.L., Jackson, C.A., Juang, B.T., Collins, K.D., Yu, M.C., L'Etoile, N.D., et al. (2013). The C. elegans cGMP-dependent protein kinase EGL-4 regulates nociceptive behavioral sensitivity. PLoS Genet. *9*, e1003619. https://doi.org/10.1371/journal.pgen.1003619.

64. Marder, E. (2012). Neuromodulation of neuronal circuits: back to the future. Neuron *76*, 1–11. https://doi.org/10.1016/j.neuron.2012.09.010.

65. Jékely, G. (2021). The chemical brain hypothesis for the origin of nervous systems. Philos. Trans. R. Soc. Lond. B Biol. Sci. *376*, 20190761. https://doi.org/10.1098/rstb.2019.0761.

66. Hökfelt, T., Broberger, C., Xu, Z.Q., Sergeyev, V., Ubink, R., and Diez, M. (2000). Neuropeptides–an overview. Neuropharmacology *39*, 1337–1356. https://doi.org/10.1016/s0028-3908(00)00010-1.

67. Hevesi, Z., Hökfelt, T., and Harkany, T. (2025). Neuropeptides: The Evergreen Jack-of-All-Trades in Neuronal Circuit Development and Regulation. BioEssays *47*, e202400238. https://doi.org/10.1002/bies.202400238.

68. Ripoll-Sánchez, L., Watteyne, J., Sun, H., Fernandez, R., Taylor, S.R., Weinreb, A., Bentley, B.L., Hammarlund, M., Miller, D.M., 3rd, Hobert, O., et al. (2023). The neuropeptidergic connectome of C. elegans. Neuron *111*, 3570–3589.e5. https://doi.org/10.1016/j.neuron.2023.09.043.

69. Watteyne, J., Chudinova, A., Ripoll-Sánchez, L., Schafer, W.R., and Beets, I. (2024). Neuropeptide signaling network of Caenorhabditis elegans: from structure to behavior. Genetics *228*, iyae141. https://doi.org/10.1093/genetics/iyae141.

70. Beets, I., Zels, S., Vandewyer, E., Demeulemeester, J., Caers, J., Baytemur, E., Courtney, A., Golinelli, L., Hasakioğulları, İ., Schafer, W. R., et al. (2023). System-wide mapping of peptide-GPCR interactions in C. elegans. Cell Rep. *42*, 113058. https://doi.org/10.1016/j.celrep.2023.113058.

71. Chaubey, A.H., Sojka, S.E., Onukwufor, J.O., Ezak, M.J., Vandermeulen, M.D., Bowitch, A., Vodičková, A., Wojtovich, A.P., and Ferkey, D.M. (2023). The Caenorhabditis elegans innexin INX-20 regulates nociceptive behavioral sensitivity. Genetics *223*, iyad017. https://doi.org/10.1093/genetics/iyad017.

72. Bhattacharya, A., Aghayeva, U., Berghoff, E.G., and Hobert, O. (2019). Plasticity of the Electrical Connectome of C. elegans. Cell *176*, 1174–1189.e16. https://doi.org/10.1016/j.cell.2018.12.024.

73. Sieburth, D., Ch'ng, Q., Dybbs, M., Tavazoie, M., Kennedy, S., Wang, D., Dupuy, D., Rual, J.F., Hill, D.E., Vidal, M., et al. (2005). Systematic analysis of genes required for synapse structure and function. Nature *436*, 510–517.

74. Laurent, P., Ch'ng, Q., Jospin, M., Chen, C., Lorenzo, R., and de Bono, M. (2018). Genetic dissection of neuropeptide cell biology at high and low activity in a defined sensory neuron. Proc Natl Acad Sci USA *115*, E6890–E6899. https://doi.org/10.1073/pnas.1714610115.

75. Sieburth, D., Madison, J.M., and Kaplan, J.M. (2007). PKC-1 regulates secretion of neuropeptides. Nat. Neurosci. *10*, 49–57. https://doi.org/10.1038/nn1810.

76. Ubink, R., Calza, L., and Hökfelt, T. (2003). 'Neuro'-peptides in glia: focus on NPY and galanin. Trends Neurosci. *26*, 604–609. https://doi.org/10.1016/j.tins.2003.09.003.

77. Yin, J.A., Gao, G., Liu, X.J., Hao, Z.Q., Li, K., Kang, X.L., Li, H., Shan, Y.H., Hu, W.L., Li, H.P., et al. (2017). Genetic variation in glia-neuron signalling modulates ageing rate. Nature *551*, 198–203. https://doi.org/10.1038/nature24463.

78. Frakes, A.E., Metcalf, M.G., Tronnes, S.U., Bar-Ziv, R., Durieux, J., Gildea, H.K., Kandahari, N., Monshietehadi, S., and Dillin, A. (2020). Four glial cells regulate ER stress resistance and longevity via neuropeptide signaling in C. elegans. Science *367*, 436–440. https://doi.org/10.1126/science.aaz6896.

79. Young, L.J., and Wang, Z. (2004). The neurobiology of pair bonding. Nat. Neurosci. *7*, 1048–1054. https://doi.org/10.1038/nn1327.

80. Bendesky, A., Kwon, Y.M., Lassance, J.M., Lewarch, C.L., Yao, S., Peterson, B.K., He, M.X., Dulac, C., and Hoekstra, H.E. (2017). The genetic basis of parental care evolution in monogamous mice. Nature *544*, 434–439. https://doi.org/10.1038/nature22074.

81. Melzer, S., Newmark, E.R., Mizuno, G.O., Hyun, M., Philson, A.C., Quiroli, E., Righetti, B., Gregory, M.R., Huang, K.W., Levasseur, J., et al. (2021). Bombesin-like peptide recruits disinhibitory cortical circuits and enhances fear memories. Cell *184*, 5622–5634.e25. https://doi.org/10.1016/j.cell.2021.09.013.

82. Kim, D.I., Park, S., Park, S., Ye, M., Chen, J.Y., Kang, S.J., Jhang, J., Hunker, A.C., Zweifel, L.S., Caron, K.M., et al. (2024). Presynaptic sensor and silencer of peptidergic transmission reveal neuropeptides as primary transmitters in pontine fear circuit. Cell *187*, 5102–5117.e16. https://doi.org/10.1016/j.cell.2024.06.035.

83. Mountoufaris, G., Nair, A., Yang, B., Kim, D.W., Vinograd, A., Kim, S., Linderman, S.W., and Anderson, D.J. (2024). A line attractor encoding a persistent internal state requires neuropeptide signaling. Cell *187*, 5998–6015.e18. https://doi.org/10.1016/j.cell.2024.08.015.

84. Auer, T.O., Khallaf, M.A., Silbering, A.F., Zappia, G., Ellis, K., Álvarez-Ocaña, R., Arguello, J.R., Hansson, B.S., Jefferis, G.S.X.E., Caron, S.J. C., et al. (2020). Olfactory receptor and circuit evolution promote host specialization. Nature *579*, 402–408. https://doi.org/10.1038/s41586-020-2073-7.

85. McGrath, P.T., Xu, Y., Ailion, M., Garrison, J.L., Butcher, R.A., and Bargmann, C.I. (2011). Parallel evolution of domesticated Caenorhabditis species targets pheromone receptor genes. Nature *477*, 321–325. https://doi.org/10.1038/nature10378.

86. Bendesky, A., and Bargmann, C.I. (2011). Genetic contributions to behavioural diversity at the gene-environment interface. Nat. Rev. Genet. *12*, 809–820. https://doi.org/10.1038/nrg3065.

87. Cande, J., Prud'homme, B., and Gompel, N. (2013). Smells like evolution: the role of chemoreceptor evolution in behavioral change. Curr. Opin. Neurobiol. *23*, 152–158. https://doi.org/10.1016/j.conb.2012.07.008.

88. Ma, F., and Zheng, C. (2023). Transcriptome age of individual cell types in Caenorhabditis elegans. Proc. Natl. Acad. Sci. USA *120*, e2216351120. https://doi.org/10.1073/pnas.2216351120.

CellPress
OPEN ACCESS

89. Bentley, B., Branicky, R., Barnes, C.L., Chew, Y.L., Yemini, E., Bullmore, E.T., Vértes, P.E., and Schafer, W.R. (2016). The Multilayer Connectome of Caenorhabditis elegans. PLoS Comput. Biol. *12*, e1005283. https://doi.org/10.1371/journal.pcbi.1005283.

90. Sasselli, V., Pachnis, V., and Burns, A.J. (2012). The enteric nervous system. Dev. Biol. *366*, 64–73. https://doi.org/10.1016/j.ydbio.2012.01.012.

91. Copenhaver, P.F. (2007). How to innervate a simple gut: familiar themes and unique aspects in the formation of the insect enteric nervous system. Dev. Dyn. *236*, 1841–1864. https://doi.org/10.1002/dvdy.21138.

92. Furness, J.B., Rivera, L.R., Cho, H.J., Bravo, D.M., and Callaghan, B. (2013). The gut as a sensory organ. Nat. Rev. Gastroenterol. Hepatol. *10*, 729–740. https://doi.org/10.1038/nrgastro.2013.180.

93. Yoo, B.B., and Mazmanian, S.K. (2017). The Enteric Network: Interactions between the Immune and Nervous Systems of the Gut. Immunity *46*, 910–926. https://doi.org/10.1016/j.immuni.2017.05.011.

94. Ludewig, A.H., and Schroeder, F.C. (2013). Ascaroside signaling in C. elegans. WormBook *2013*, 1–22. https://doi.org/10.1895/wormbook.1.155.1.

95. Ben-David, E., Boocock, J., Guo, L., Zdraljevic, S., Bloom, J.S., and Kruglyak, L. (2021). Whole-organism eQTL mapping at cellular resolution with single-cell sequencing. eLife *10*, e65857. https://doi.org/10.7554/eLife.65857.

96. Ghanta, K.S., and Mello, C.C. (2020). Melting dsDNA Donor Molecules Greatly Improves Precision Genome Editing in Caenorhabditis elegans. Genetics *216*, 643–650. https://doi.org/10.1534/genetics.120.303564.

97. Eroglu, M., Yu, B., and Derry, W.B. (2023). Efficient CRISPR/Cas9 mediated large insertions using long single-stranded oligonucleotide donors in C. elegans. FEBS Journal *290*, 4429–4439. https://doi.org/10.1111/febs.16876.

98. Schindelin, J., Arganda-Carreras, I., Frise, E., Kaynig, V., Longair, M., Pietzsch, T., Preibisch, S., Rueden, C., Saalfeld, S., Schmid, B., et al. (2012). Fiji: an open-source platform for biological-image analysis. Nat. Methods *9*, 676–682. https://doi.org/10.1038/nmeth.2019.

99. Sternberg, P.W., Van Auken, K., Wang, Q., Wright, A., Yook, K., Zarowiecki, M., Arnaboldi, V., Becerra, A., Brown, S., Cain, S., et al. (2024). WormBase 2024: status and transitioning to Alliance infrastructure. Genetics *227*, iyae050. https://doi.org/10.1093/genetics/iyae050.

100. Ben-David, E., Pliota, P., Widen, S.A., Koreshova, A., Lemus-Vergara, T., Verpukhovskiy, P., Mandali, S., Braendle, C., Burga, A., and Kruglyak, L. (2021). Ubiquitous Selfish Toxin-Antidote Elements in Caenorhabditis Species. Curr. Biol. *31*, 990–1001.e5. https://doi.org/10.1016/j.cub.2020.12.013.

101. Stevens, L., Moya, N.D., Tanny, R.E., Gibson, S.B., Tracey, A., Na, H., Chitrakar, R., Dekker, J., Walhout, A.J.M., Baugh, L.R., et al. (2022). Chromosome-Level Reference Genomes for Two Strains of Caenorhabditis briggsae: An Improved Platform for Comparative Genomics. Genome Biol. Evol. *14*, evac042. https://doi.org/10.1093/gbe/evac042.

102. Stein, L.D., Bao, Z., Blasiar, D., Blumenthal, T., Brent, M.R., Chen, N., Chinwalla, A., Clarke, L., Clee, C., Coghlan, A., et al. (2003). The Genome Sequence of Caenorhabditis briggsae: A Platform for Comparative Genomics. PLoS Biol. *1*, E45. https://doi.org/10.1371/journal.pbio.0000045.

103. Emms, D.M., and Kelly, S. (2019). OrthoFinder: phylogenetic orthology inference for comparative genomics. Genome Biol. *20*, 238. https://doi.org/10.1186/s13059-019-1832-y.

104. Conover, J.L., Sharbrough, J., and Wendel, J.F. (2021). pSONIC: Ploidy-aware Syntenic Orthologous Networks Identified via Collinearity. G3 (Bethesda) *11*, jkab170. https://doi.org/10.1093/g3journal/jkab170.

105. Qiu, X., Hill, A., Packer, J., Lin, D., Ma, Y.A., and Trapnell, C. (2017). Single-cell mRNA quantification and differential analysis with Census. Nat. Methods *14*, 309–315. https://doi.org/10.1038/nmeth.4150.

106. Trapnell, C., Cacchiarelli, D., Grimsby, J., Pokharel, P., Li, S., Morse, M., Lennon, N.J., Livak, K.J., Mikkelsen, T.S., and Rinn, J.L. (2014). The dynamics and regulators of cell fate decisions are revealed by pseudotemporal ordering of single cells. Nat. Biotechnol. *32*, 381–386. https://doi.org/10.1038/nbt.2859.

107. Cao, J., Spielmann, M., Qiu, X., Huang, X., Ibrahim, D.M., Hill, A.J., Zhang, F., Mundlos, S., Christiansen, L., Steemers, F.J., et al. (2019). The single-cell transcriptional landscape of mammalian organogenesis. Nature *566*, 496–502. https://doi.org/10.1038/s41586-019-0969-x.

108. Wolock, S.L., Lopez, R., and Klein, A.M. (2019). Scrublet: Computational Identification of Cell Doublets in Single-Cell Transcriptomic Data. Cell Syst. *8*, 281–291.e9. https://doi.org/10.1016/j.cels.2018.11.005.

109. Young, M.D., and Behjati, S. (2020). SoupX removes ambient RNA contamination from droplet-based single-cell RNA sequencing data. GigaScience *9*, giaa151. https://doi.org/10.1093/gigascience/giaa151.

110. Kolde, R. (2015). Package 'pheatmap'. R package 1.7, 790. https://cran.r-project.org/web/packages/pheatmap/pheatmap.pdf.

111. Haghverdi, L., Lun, A.T.L., Morgan, M.D., and Marioni, J.C. (2018). Batch effects in single-cell RNA-sequencing data are corrected by matching mutual nearest neighbors. Nat. Biotechnol. *36*, 421–427. https://doi.org/10.1038/nbt.4091.

112. Tarashansky, A.J., Musser, J.M., Khariton, M., Li, P., Arendt, D., Quake, S.R., and Wang, B. (2021). Mapping single-cell atlases throughout Metazoa unravels cell type evolution. eLife *10*, e66747. https://doi.org/10.7554/eLife.66747.

113. Di Tommaso, P., Moretti, S., Xenarios, I., Orobitg, M., Montanyola, A., Chang, J.M., Taly, J.F., and Notredame, C. (2011). T-Coffee: a web server for the multiple sequence alignment of protein and RNA sequences using structural information and homology extension. Nucleic Acids Res. *39*, W13–W17. https://doi.org/10.1093/nar/gkr245.

114. Dereeper, A., Guignon, V., Blanc, G., Audic, S., Buffet, S., Chevenet, F., Dufayard, J.F., Guindon, S., Lefort, V., Lescot, M., et al. (2008). Phylogeny.fr: robust phylogenetic analysis for the non-specialist. Nucleic Acids Res. *36*, W465–W469. https://doi.org/10.1093/nar/gkn180.

115. Smith, C.J., Watson, J.D., Spencer, W.C., O'Brien, T., Cha, B., Albeg, A., Treinin, M., and Miller, D.M., 3rd. (2010). Time-lapse imaging and cell-specific expression profiling reveal dynamic branching and molecular determinants of a multi-dendritic nociceptor in C. elegans. Dev. Biol. *345*, 18–33. https://doi.org/10.1016/j.ydbio.2010.05.502.

116. Mulcahy, B., Witvliet, D.K., Mitchell, J., Schalek, R., Berger, D.R., Wu, Y., Holmyard, D., Lu, Y., Ahamed, T., Samuel, A.D.T., et al. (2022). Post-embryonic remodeling of the C. elegans motor circuit. Curr. Biol. *32*, 4645–4659.e3. https://doi.org/10.1016/j.cub.2022.09.065.

117. Rashid, A., Tevlin, M., Lu, Y., and Shaham, S. (2022). A developmental pathway for epithelial-to-motoneuron transformation in C. elegans. Cell Rep. *40*, 111414. https://doi.org/10.1016/j.celrep.2022.111414.

118. Rubinov, M., and Sporns, O. (2010). Complex network measures of brain connectivity: uses and interpretations. Neuroimage *52*, 1059–1069. https://doi.org/10.1016/j.neuroimage.2009.10.003.

119. Teufel, F., Almagro Armenteros, J.J., Johansen, A.R., Gíslason, M.H., Pihl, S.I., Tsirigos, K.D., Winther, O., Brunak, S., von Heijne, G., and Nielsen, H. (2022). SignalP 6.0 predicts all five types of signal peptides using protein language models. Nat. Biotechnol. *40*, 1023–1025. https://doi.org/10.1038/s41587-021-01156-3.

120. Hallgren, J., Tsirigos, K.D., Pedersen, M.D., Almagro Armenteros, J.J., Marcatili, P., Nielsen, H., Krogh, A., and Winther, O. (2022). DeepTMHMM predicts alpha and beta transmembrane proteins using deep neural networks. Preprint at bioRxiv. https://doi.org/10.1101/2022.04.08.487609.

121. Jones, P., Binns, D., Chang, H.Y., Fraser, M., Li, W., McAnulla, C., McWilliam, H., Maslen, J., Mitchell, A., Nuka, G., et al. (2014). InterProScan 5: genome-scale protein function classification. Bioinformatics *30*, 1236–1240. https://doi.org/10.1093/bioinformatics/btu031.

STAR★METHODS

KEY RESOURCES TABLE

REAGENT or RESOURCE	SOURCE	IDENTIFIER
**Bacterial and virus strains**		
E. coli	Caenorhabditis Genetics Center (CGC)	WormBase: OP50; WormBase: WBStrain00041969
**Chemicals, peptides, and recombinant proteins**		
Sodium Azide	Sigma-Aldrich	Cat# 71289
Pronase E	Sigma-Aldrich	Cat# P8811
Alt-R S.p. Cas9 Nuclease V3	IDT	Cat# 1081059
Alt-R CRISPR-Cas9 tracrRNA	IDT	Cat# 1072532
Alt-R™ L.b. Cas12a crRNA	IDT	Cat# 10007922
Q5® High-Fidelity DNA Polymerase	New England Biolabs	Cat# M0491
Lambda Exonuclease	New England Biolabs	Cat# M0262
Vybrant™ DiD Cell-Labeling Solution	Thermo Fisher Scientific	Cat# V22887
**Critical commercial assays**		
PureLink™ PCR Purification Kit	Thermo Fisher Scientific	Cat# K310001
Monarch® Genomic DNA Purification Kit	New England Biolabs	Cat# T3010
Chromium™ Next GEM Single Cell 3’ Kit v3.1	10x Genomics	Cat# 1000269
**Deposited data**		
Raw sequencing data	This paper	https://www.ncbi.nlm.nih.gov/bioproject/PRJNA851520.
Annotated cell datasets	This paper	https://doi.org/10.5281/zenodo.14194525
Scripts used in this study	This paper	https://doi.org/10.5281/zenodo.14205685
Website for exploration of expression data	This paper	https://caenogen.shinyapps.io/caenogen/
**Experimental models: Organisms/strains**		
Nematode strains used in this study	Data S4A	N/A
**Oligonucleotides**		
crRNAs and ssODN sequences used in study	Data S4B	N/A
**Recombinant DNA**		
Plasmids used in study	Data S4C	N/A
**Software and algorithms**		
FIJI V2.9	Schindelin et al.[98]	https://imagej.net/software/fiji/
Funannotate (v.1.8.7)	PalmerJM & Stajich J	https://github.com/nextgenusfs/funannotate/
Cellranger (v.7.0.1)	10x Genomics	https://www.10xgenomics.com
Orthofinder	Emms and Kelly[103]	https://github.com/davidemms/OrthoFinder
pSonic	Conover et al.[104]	https://github.com/conJUSTover/pSONIC
Monocle3 (v.1.3.4)	Cao et al.[107]	https://cole-trapnell-lab.github.io/monocle3/
pheatmap (v.1.0.12)	Kolde[110]	https://cran.r-project.org/web/packages/pheatmap/index.html
SAMap(v.1.0.15)	Tarashansky et al.[112]	https://github.com/atarashansky/SAMap
InterProScan	Jones et al.[121]	https://www.ebi.ac.uk/interpro/
T-Coffee (v.11.00)	Di Tommaso et al.[113]	https://tcoffee.crg.eu/
phylogeny.fr	Dereeper et al.[114]	https://www.phylogeny.fr/
SignalP (v.6.0)	Teufel et al.[119]	https://services.healthtech.dtu.dk/services/SignalP-6.0/

(*Continued on next page*)

Continued

REAGENT or RESOURCE	SOURCE	IDENTIFIER
DeepTMHMM (v.1.0)	Hallgren et al.[120]	https://services.healthtech.dtu.dk/services/DeepTMHMM-1.0/
MATLAB v24.1.0.2578822 (R2024a)	The MathWorks Inc	https://www.mathworks.com/
Dunn.test	Alexis Dinno	https://cran.r-project.org/package=dunn.test
scico (v.1.5.0)	Crameri F	https://github.com/thomasp85/scico
Other		
Sequencing system	Illumina	Novaseq 6000
Dissection microscope	Leica	M165FC
Upright compound microscope	Zeiss	Axio Imager Z2
Confocal laser scanning microscope	Zeiss	LSM 880 & LSM 980

EXPERIMENTAL MODEL AND STUDY PARTICIPANT DETAILS

Cultivation of nematodes for scRNA-seq

C. elegans (strain N2), *C. briggsae* (AF16) and *C. tropicalis* (NIC203) were cultured at 20°C with *E. coli* strain OP50 using standard conditions with the exception that the agar in the nematode growth media (NGM) was replaced with a 4:6 mixture of agarose and agar (NGM+agarose) to prevent burrowing. To generate a large synchronized population of L2 worms for single-cell RNA-seq (scRNA-seq) experiments, adult hermaphrodites from the three strains were treated with hypochlorite solution and the resulting embryos were kept overnight (~16h) in M9 buffer to hatch and arrest in L1 stage. Then, L1s were transferred to 10cm plates pre-seeded with OP50 (4 plates, 50,000 worms/plate, total of 200,000 worms per strain). After 23 hours, the L2 stage was verified under a stereoscope.

METHOD DETAILS

Cell dissociation for scRNA-seq

Cell dissociation was carried out as previously described[95] with minor modifications for the three worm species. L2 worms were recovered off the plates and washed three times in M9 in a 15ml conical tube, followed by two times in a 1.5ml tube. Lysis was then performed using a freshly thawed aliquot of 200μl SDS-DTT solution (200 mM DTT, 0.25% SDS, 20 mM HEPES, pH 8.0, 3% sucrose) for 6 minutes in a hula mixer set on low speed. Worms were then washed quickly three times in 1ml of M9, and two additional times in 1ml of egg buffer (118 mM NaCl, 48 mM KCl, 2 mM CaCl2, 2 mM MgCl2, 25 mM HEPES, pH 7.3, osmolarity adjusted to 340 mOsm with sucrose). Worms were then resuspended in 500μl of 20 mg/ml Pronase E that was freshly prepared in L15-FBS (L15 medium supplanted with 2% fetal bovine serum and adjusted to 340 mOsm with sucrose). Worm dissociation was done by continuous pipetting on the side of the tube. The dissociation process was monitored every 2-3 min on a microscope equipped with a x40 phase contrast objective lens. Dissociation was stopped when a high density of cells was visible. Dissociation durations were different for each species: 8:36 for *C. elegans*, 13:16 for *C. tropicalis*, and 17:16 for *C. briggsae*. Dissociations were stopped by adding 500μl ice-cold L15-FBS and transferring the tubes on ice. Following dissociation, lysates were spun for 6 min at 500g at 4°C. Cell pellets were resuspended in cold PBS (adjusted to 340 mOsm with sucrose). Cell suspensions were spun for 1 min in 50g to pellet remaining undigested worms. Cell preparations (supernatants) were transferred to new 1.5ml tubes (pre-cooled on ice) and cells were counted on a hemocytometer loaded to an inverted microscope equipped with differential interference contrast (DIC). Cells from each species were then diluted to 10^6 cells/ml concentration in osmolarity-adjusted PBS, and then combined together. The combined pool was loaded onto eight lanes of 3′ Chromium scRNA-cel flow cells (10x Genomics), targeting 30,000 cells on each lane. Each lane is considered a technical replicate. Library prep was carried out according to the manufacturer's protocol. Libraries were sequenced together on three S4 lanes of Novaseq 6000. Paired-end 2 × 150 runs were done to maximize the recovery of transcript variants between the three species.

Genome engineering and transgenics

Knock-in reporter alleles were generated using CRISPR/Cas9 or Cas12a and single-stranded oligodeoxynucleotides (ssODNs) for precise insertions. Injection mixtures were prepared using enzymes and RNAs ordered from IDT (Cas9 #1081059, L.b.Cas12a #10007922, tracrRNA #1072532) and according to the injection procedure and concentrations described in Ghanta and Mello[96]. Cas9 (0.5μl of 10μg/μl stock), tracrRNA (5μl of 0.4 μg/μl stock) and crRNA (2.8μl of 0.4 μg/μl stock) were gently mixed together and left to incubate at 37°C for 15 minutes to form the RNP complex. The ssODN (2.2μg) was then added and mixture complemented with nuclease-free water to a final volume of 20μl, used for microinjection. F1 progeny of injected hermaphrodites were screened for engineered heterozygotes through examination under a fluorescent dissection microscope (Leica M165FC) and PCR genotyping,

before isolation of homozygotes in subsequent generations. All insertions in the resulting strains were validated by Sanger sequencing.

The ssODNs used as CRISPR repair donor templates were relatively long (typically 0.9kb~1.6kb). They all included the desired insertion sequence (including mutations in the PAM or crRNA complementary region when needed) flanked by 35bp-homology arms. Long ssODNs were prepared using a previously-described procedure based on the enzymatic digestion of PCR products into single-stranded DNA.[97] We designed PCRs amplifying exactly the desired repair template sequence with one (but not both) primer bearing a phosphorylated 5' nucleotide. PCRs were run in 50μl reactions (4 side-by-side replicates) using Q5 enzyme and buffers (NEB; M0491). Amplification was confirmed through agarose gel electrophoresis, then the replicates were pooled together and column-purified (Invitrogen™ PureLink™ #K310001). Eluted purified PCR products (45μl in nuclease-free water) were digested with a lambda exonuclease (NEB M0262L) and accompanying reaction buffer for 20 minutes at 37°C (total reaction volume 50μl). Lambda exonuclease is a strand-specific exonuclease that preferentially degrades DNA strands phosphorylated at their 5' end. Enzymatic digestion was followed by column purification using the Monarch® system (NEB T3010S) and elution with 6μl of nuclease-free water.

Transgenic strains expressing NSSP genes fused to tagRFP in the motor neurons of the ventral nerve cord were obtained by co-injecting the NSSP expression plasmid (50ng/μl) and pha-1(+) rescue construct (50ng/μl) into *pha-1(e2123) C. elegans* worms and growing them at 25°C (lethal temperature for non-rescued animals). The structure of all NSSP transgenes was: *unc-129p::NSSP::tagRFP::sl2::gfp::h2b*. An *ins-1* fusion construct was used as positive control (established secreted neuropeptide) and a non-fused *tagRFP* cassette as negative control. Information about strains, crRNAs, ssODNs and plasmids used for this study are found in Data S4A–S4C.

Microscope imaging and analysis

Animals were anesthetized in a drop of M9 with 50mM sodium azide and mounted on glass slides padded with a patch of 5% agarose. Images of strains bearing fluorescent endogenous reporter alleles were acquired using Zeiss confocal microscopes LSM880 or LSM980 with a 40x water objective. FIJI software[98] was used to analyze neuron-specific expression and to generate orthogonal Z max projections of representative images. To facilitate neuron identification, animals were stained with Vybrant™ DiD cell-labeling solution (Invitrogen # V22887) and/or crossed with strains expressing fluorescently-tagged genes in conserved sets of cells. Strains resulting from these crosses are described in Data S4A. Images of NSSP::tagRFP transgenic worms and controls were acquired using a Zeiss Axio Imager Z2 microscope at x40 objective. Images were analyzed to characterize uptake of TagRFP in coelomocytes and for punctate/dispersed expression in the dorsal nerve cord. *nlp-18::gfp* animals were imaged as L2s, NSSP::tagRFP transgenic nematodes were imaged as L4s or young adults, all other images were taken using L4 nematodes.

Genome builds and *in silico* demultiplexing

The raw sequencing data included reads originating from all three species. To generate species-specific fastq files, we first aligned the reads separately to the *C. elegans* genome (WBcel235),[99] the *C. tropicalis* NIC203 genome,[100] and the recently published *C. briggsae* QX1410 genome[101] that was annotated *de-novo* using Funannotate V1.8.7 (https://github.com/nextgenusfs/funannotate/). The longest mRNA isoform of each gene was used for alignment. The gene transfer files (GTF) annotation of 3'UTR regions in *C. briggsae and C. tropicalis* were then dynamically extended to improve the mapping of 3' scRNA-seq reads, following the same iterative approach outlined in a previous study for *C. elegans*.[41] The alignment to the different species was compared based on the CIGAR string in the alignment bam file, and reads that supported one of the species better than the two others were used to generate three species-specific sets of fastq files. Alignment and counting were then performed using Cellranger v.7.0.1 (10x Genomics).

Identification of gene ortholog sets

To identify gene orthologs, we took advantage of the broad synteny previously reported between *C. elegans* and each of the other two species.[100,102] We carried out a tiered analysis that considered multiple possible scenarios for orthologous genes and ranked them by the strength of the evidence. Our analysis included both synteny-agnostic orthology discovery with Orthofinder[103] and synteny-aware orthology discovery using pSONIC.[104] We carried out ortholog gene discovery with each of the tools using either the transcriptome (cDNA) sequences or the proteome (amino acid) sequences. Evidence for orthology was ranked into 5 "levels" based on the source using the following encoding:

1. Unanimous orthologs, identified unequivocally with a 1:1 mapping across all analyses.
2. Syntenic orthologs discovered based on cDNA sequence.
3. Syntenic orthologs discovered based on protein sequence.
4. Non-syntenic orthologs discovered based on cDNA sequence.
5. Non-syntenic orthologs discovered based on protein sequence.

These "orthology levels" are hierarchical in the sense that the supporting evidence for the assignment of a 1:1 orthology relationship for a pair of genes is stronger in "level 4" orthologs than for "level 5 orthologs", and further stronger in "level 3 orthologs", etc. We then used this annotation list to call a consensus set of 1:1 orthologs using the following algorithm: For every gene (A) in every

species, (A) was considered a consensus 1:1 ortholog of a gene (A') in another species if (A) was found homologous only to (A') when considering the "orthology levels" in hierarchical order, and if no other gene (B) was called a consensus 1:1 ortholog to gene (A') in the same nor in a more stringent "orthology level". Orthology data for all genes are available in Data S4D–S4F. The final set of 1:1:1 ortholog gene triplets (across all three species) included 11,277 triplets.

Genes that had no 1:1 ortholog genes whatsoever in the two other species in all "orthology levels" tested were considered "1-to-none" novel genes. The "permissive sets" of genes (Figures 3B and 3C) included all genes that had no consensual 1:1 orthologs in neither of the two other species. Hence, the permissive sets included 1-to-none novel genes as well as genes in orthogroups that include recently-duplicated paralogs or multiple closely-related paralogs in both sequence and synteny. Genes that had a consensual 1:1 ortholog in a second but not a third species were left out of the permissive sets. Orthology information for all genes is available in Data S4D–S4F.

Downstream processing and cell type annotation

C. elegans cells differ widely in the number of Unique Molecular Identifiers (UMIs) that are recovered in scRNA-seq. As a result, a simple UMI cutoff may be biased for cell types with more UMIs.[41] Therefore, we implemented an iterative pipeline to recover clusters of high-quality cells and remove cell doublets and degraded cells. For each species, we took 25,000 cells with most UMIs in each lane (2.5x the targeted number of cells, 200,000 overall) and processed them in *Monocle3*(v.1.3.4) available at https://cole-trapnell-lab.github.io/monocle3/.[105–107] We used default parameters for processing (preprocessing, dimensionality reduction and clustering) except that 100 dimensions were included for PCA. We used the *top_markers* function in *Monocle3* to identify enriched genes in the obtained clusters (Leiden clustering), and removed clusters whose top enriched genes were mostly ribosomal or mitochondrial genes and no known cell type markers. We repeated this cell filtering step a second time with the remaining cells. We then used scrublet(v.0.2.3)[108] to remove suspected cell doublets (doublet score > 0.1) and SoupX(v1.6.2)[109] to correct for background contamination by ambient RNAs, reprocessing the datasets after each step. Resulting datasets were used for manual cell type annotations. During downstream analyses, we noticed several cell types missing in our data due to low UMI counts of *Caenorhabditis* neurons, as previously reported.[41] To recover those cells, we used markers co-expressed very specifically in individual neurons to identify them in the raw expression matrix and then reintegrated those cells into the filtered datasets ("reincluded" column in the annotated datasets). The full datasets, including the reintegrated cells, were reprocessed, clustered, corrected for doublets and ambient RNAs before final cell annotations and analyses.

To annotate cell types, we compared the enriched differential genes in each cluster to markers confirmed in previous *C. elegans* studies,[40,41] or their 1:1 orthologs for *C. briggsae* and *C. tropicalis*. After assigning clusters into broad tissue types, all cells of a given tissue (for example all neurons) were further subclustered in isolation to separate refined clusters and cell types. Most neuronal clusters could be readily assigned to an individual neuron class or subclass. In other cases, we applied more than one cycle of separated subclustering. When clusters appeared to contain cells from several closely-related neuron classes that couldn't be confidently separated (such as the oxygen sensing neurons AQR/PQR/URX, or the dopaminergic neurons ADE/PDE/CEP) we annotated them accordingly as a group. In some cases, the granular separation of neuron classes was of higher resolution in one species compared to others. For example, the chemorepulsive neurons ASH, PHA and PHB clustered separately in the *C. elegans* dataset but could not be confidently separated in the other species. Therefore, in the count matrices metadata of each species, we generate distinct columns for the highest-resolution annotations in each species ("within_species" column, e.g. separate ASH, PHA & PHB) and annotations that regrouped cells into common annotations across species ("cross_species" column, e.g. ASH_PHA_PHB). Species-specific analyses (for example in Figures 3A–3C) relied on the former, while cross-species comparisons (the majority of analyses presented) relied on the latter. Cross-species comparisons also excluded neuron classes that included no or only very few sequenced cells in the dataset of one of the species (*C. tropicalis* ASE & I3, *C. briggsae* AWA). Lists of top differential genes (*top_markers* outputs) are available in Data S1B–S1G.

Integrations of datasets across species

We used two alternative approaches to integrate the datasets.

Monocle3/*Batchelor*.[111] The datasets were first subsetted to include only genes with 1:1:1 orthologs and only cells that were annotated as belonging to neuron types that we detected in all datasets ("cross_species" comparisons). We then preprocessed the integrated datasets (using 100 dimensions as above) and used align_cds() in Monocle3, which relies on the mutual nearest neighbor algorithm Batchelor, with default settings and with species as "alignment_group". We then proceeded with dimensionality reduction and clustering as above to generate UMAP projections (per species and per neuron type). We also used the integrated clustering results to quantify, for every neuron type in a given species, what proportion of cells from this type was assigned to the cluster that contains the majority of the cells of a given neuron type across all species.

SAMap(v.1.0.15).[112] We generated databases of pairwise gene alignments using *map_genes.sh*. Then we ran SAMap on the raw datasets that included only cells annotated as belonging to the "cross_species" set (*run* with pairwise=True). Mapping scores and coordinates for UMAP projections were extracted from the resulting SAMap object.

Classification of genes into gene families

C. elegans genes and their orthologs in *C. briggsae* and *C. tropicalis* were assigned to neuronal gene families of interest based on previous research in *C. elegans*.[55] In addition, we used the InterProScan pipeline (https://www.ebi.ac.uk/interpro/) to annotate

protein domains based on the predicted translated sequences of all three species using PFAM and the PFAM-A database. We then applied a custom-made pipeline to assign genes to gene families based on family-defining protein domains. Tables for all genes and their respective orthologs, protein domains and annotated families are available in Data S4D–S4F. To generate schematic phylogenetic trees of gene orthogroups, we first run multiple-sequence alignment (T-Coffee v.11.00)[113] using protein sequences. The MSA output was used to generate trees at phylogeny.fr[114] with default parameters.

Divergence of neurotransmitter receptors

Lists and details about neurotransmitter receptors in *C. elegans* are found in Data S2A and S2B. Genes with 1:1:1 orthologs were selected and classified according to their category (excitatory ionotropic receptors, inhibitory ionotropic receptors, modulatory metabotropic receptors) and their neurotransmitter ligand (glutamate, GABA, biogenic monoamines). Acetylcholine receptors were left out because some of them (such as *unc-38*, *unc-63* & *acr-12*, shown in Figure S5) were found to be expressed very broadly across the nervous system, mostly at low expression levels, which makes our divergence analysis hard to conclusively interpret for these cases. Thresholded data was used to quantify how many receptors of each category are expressed in each neuron class and species. Receptivity was considered altered across species in a ligand/activity category if at least one species expresses at least 2 receptors in the category and one species expresses 0 receptors in the category.

Neuropeptidergic connectomes

Neuropeptide network spatial constraining

Neuropeptidergic signaling was locally thresholded to filter out connections between neurons that were anatomically distant from each other, based on electron microscopy data and neuronal reporter strains of the *C. elegans* nervous system in L2 stage (23h after hatching).[38,115–117] These data were used to create a matrix of locations and anatomical proximity for the processes of each neuron, identifying 27 different neuronal process bundles in the *Caenorhabditis* nervous system as previously defined.[49] This classification was then used to filter out neuropeptidergic connections based on putative signaling ranges in the three species. The stringent short-range thresholding allows connections only between neuronal processes that are in the same process bundle, and the pharynx is a separated system where connections are allowed between pharyngeal neurons only. The mid-range stringency thresholding allows connections between neurons with neuronal processes in the same anatomical area: head (including pharynx and the ventral cord neurons that are in the ventral ganglion), midbody and tail.

Neuropeptide network construction

A previous study biochemically validated interactions between *C. elegans* neuropeptide ligands and GPCR receptors.[70] Out of these validated interaction pairs, we used thresholded expression data for the 42 neuropeptide precursor genes (NPP) and 47 GPCR receptors that had 1:1:1 orthologs in our datasets. Adjacency matrices were built using a binary version of the expression data for the 285 single neurons present in the datasets of the three species. For a given point $A(i, j)^N$ and for a given NPP–GPCR pair N the connection between two neurons is defined by $A(i, j)^N = NPP(i, j)^N \times GPCR(i, j)^N$. Each NPP-GPCR interaction forms an individual binary network. To generate global neuropeptide networks, we summed each individual NPP-GPCR network resulting in weighted networks in which the weight indicates the number of NPP-GPCR pairs that connect two nodes (cells). Reciprocal connections between nodes were considered as two separate unidirectional connections. For the cross-species network analysis, we binarized all the connections (ON if weight of connection ≥ 1) of each species' network separately, then integrated the data into a single network reflecting the cross-species conservation pattern of connections between homologous neurons.

Topological network measures: Degree

Edge counts and adjacency matrices were all computed using binary directed versions of the networks. The same networks were used to compute degree using the method from the Brain Connectivity Toolbox[118] for MATLAB. Degree is the number of edges connected to a given node. In-degree is the number of incoming connections connected to a given node and out-degree is the number of outgoing connections.

Orphan GPCRs expressed in non-sensory neurons

Lists of all GPCR genes in each species were filtered to remove neurotransmitter- and neuropeptide- binding receptors and their paralogs. Then, all genes with no strong expression anywhere were removed (genes never expressed in more than 10% of sequenced cells of a specific cell type, across all cell types). Expression patterns of remaining GPCR genes were manually examined to generate lists of GPCRs expressed in non-sensory neurons.

Identification of NSSPs

In each species, we first selected all transcripts of genes for which the predicted translated coding sequence is of less than 200 amino acids. Sequences were filtered for the presence of a signal peptide using SignalP(v.6.0)[119] and the absence of a transmembrane domain using DeepTMHMM(v.1.0).[120] All established neuropeptide precursor genes (and their paralogs) and all genes with domains detected through PFAM were removed except for DUF ("domain of unknown function")-containing genes. Then, all cells in whole-animal datasets were classified into neuronal and non-neuronal cells, and normalized expression levels for all genes were pseudo-bulked according to this classification. Expression values were then examined in comparison to the set of neuropeptide precursor genes having a 1:1:1 in all three species. In each species, a gene from the filtered subset of small secreted genes was considered NSSP if its neuronal enrichment was ≥ 2 and if its normalized expression level in the nervous system was above the 10% decile value

of the 1:1:1 neuropeptide genes (i.e., expression value above which 90% of the genomically conserved neuropeptide precursor genes are found). A slightly more stringent cutoff was used in *C. briggsae* (including 85% of the conserved neuropeptide precursor genes) as this made the cutoff more consistent relative to the observed expression distributions of the two other species. Resulting lists of genes are found in Data S3C and S3D. Small secreted proteins enriched in the sheath glia were detected by manual inspection of the sets of genes obtained with same the criteria as above except that neuronal enrichment was set to <2 (neuronally-depleted or mildly-enriched).

QUANTIFICATION AND STATISTICAL ANALYSIS

Transcriptomic correlation heatmaps

In each species, we pseudobulked the normalized expression values calculated from *Monocle3* for all genes in every neuron class (cross_species neuron annotations). Then, we collected all genes with 1:1:1 orthologs that had a top_markers score > 0.1 in at least one neuron class and in at least one species (1,380 1:1:1 orthologs in total). The pheatmap(v.1.0.12) function[110] on pearson correlations of pseudocount normalized values were used for hierarchical clustering and visualizations.

Thresholding & Jaccard distance calculations

To threshold expression data into binary ON/OFF expression values, we trained a random forest classifier on ground truth data from *C. elegans*. Our ground truth expression matrix was based on the dataset previously generated by the CeNGEN project[40] which itself had been compiled based experimentally-validated fluorescent reporters. The original ground truth matrix was modified to account for differences in the grouping of neuronal classes and for recent updates in characterizations of expression patterns.[52] To train the random forest model, we included features at the cell-cluster level and at the gene level. At the cell-cluster level, our features included the following matrices:

A) A z-score standardized matrix of normalized expression values that was binarized to include 1 in cells with z>1.5
B) For each gene, the fraction of expressing cells (defined as having raw counts > 0) in each cell type.
C) For each gene, the number of expressing cells (defined as having raw counts > 0) in each cell type.
D) The pseudobulked count matrix
E) For each gene in each cell type, the fraction of cells with counts > 0. That matrix was further normalized by the maximum fraction observed for each gene ("percentile thresholding" in Taylor et al.[40]).

Gene level features:

F) the variance of the pseudobulked expression matrix
G) the variance of the pseudobulked normalized expression matrix
H) the total number of cells with expression of the gene.

For training the classifier, the genes were split to Training+Validation/Test sets (75%,25%). We trained a random forest classifier with 1000 trees using 10-fold cross validation regime on the training data. On the test set of unseen genes with ground truth knowledge in *C. elegans*, our classifier achieved 98% precision and 89% recall.

The classifier was used to infer binarized expression in all genes in all three species.

Thresholded expression data of 1:1:1 orthologs was aggregated across species and used to calculate Jaccard distances according to the formula $\frac{(union - intersect)}{union}$. More explicitly:

$$\frac{(\# \text{ of cell types in which gene is ON in at least one species}) - (\# \text{ of cell types in which gene is ON in all three species})}{(\# \text{ of cell types in which gene is ON in at least one species})}$$

Cell$-$centered Jaccard distances for each cell type were calculated as :

$$\frac{(\# \text{ of genes that are ON in the cell type in at least one species}) - (\# \text{ of genes that are ON in the cell type in all three species})}{(\# \text{ of genes that are ON in the cell type in at least one species})}$$

Genes included for the cell-centered Jaccard distances were either all 1:1:1 orthologs expressed anywhere in the nervous system (at least one neuron in any of the three species, 9666 genes) or the subset of 1:1:1 orthologs belonging to gene families with established roles in neuronal activity (884 genes, see Hobert[55]). Jaccard distances for pairwise comparisons of species (3 pairs in total) were calculated similarly but included only two species instead of three.

Statistics analyses and graphing

Neuropeptide network plots were generated using MATLAB (v24.1.0.2578822 (R2024a), The MathWorks Inc., Natick, MA). All other statistical analyses and plots were done in R v.4.2.2. Statistical details for each analysis appear in figure legends. We mostly used the non-parametric Kruskal-Wallis test with Dunn's post hoc test and the Benjamini-Hochberg method to correct for multiple

comparisons (function dunn.test with method “bh”) (https://cran.r-project.org/package=dunn.test) and differences were considered statistically significant if $p < 0.025$ ($\alpha/2$). When only two groups were compared, we used the Wilcoxon rank sum test (wilcox.test). For categorically-scored expression of fluorescent reporters out of all tested animals, we used Fisher’s test (fisher.test) with Benjamini-Hochberg correction for multiple comparisons.

ADDITIONAL RESOURCES

Raw sequencing data: https://www.ncbi.nlm.nih.gov/bioproject/PRJNA851520.
Annotated cell datasets: https://doi.org/10.5281/zenodo.14194525.
Scripts used for study: https://doi.org/10.5281/zenodo.14205685.
Shinyapp to explore expression data: https://caenogen.shinyapps.io/caenogen/.

Current Biology, Volume *35*

Supplemental Information

Divergence in neuronal signaling pathways despite conserved neuronal identity among *Caenorhabditis* species

Itai Antoine Toker, Lidia Ripoll-Sánchez, Luke T. Geiger, Antoine Sussfeld, Karan S. Saini, Isabel Beets, Petra E. Vértes, William R. Schafer, Eyal Ben-David, and Oliver Hobert

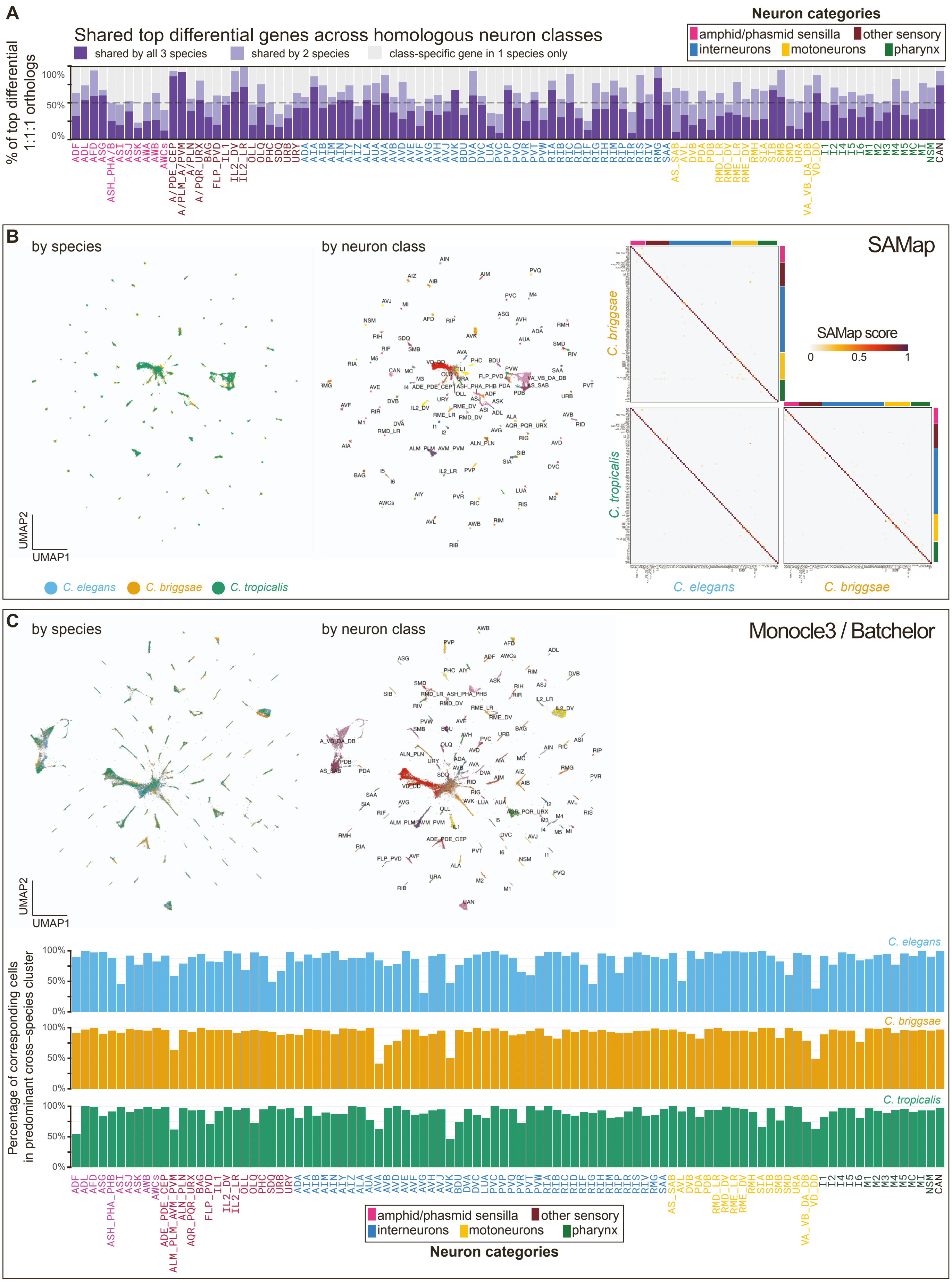
A
Shared top differential genes across homologous neuron classes
shared by all 3 species
shared by 2 species
class-specific gene in 1 species only
% of top differential 1:1:1 orthologs
100%
50%
0%
Neuron categories
amphid/phasmid sensilla
other sensory
interneurons
motoneurons
pharynx
B
by species
by neuron class
SAMap
SAMap score
0
0.5
1
C. briggsae
C. tropicalis
C. elegans
UMAP2
UMAP1
C
Monocle3 / Batchelor
Percentage of corresponding cells in predominant cross-species cluster
C. elegans
C. briggsae
C. tropicalis
Neuron categories

Figure S1. Shared differential genes and cross-species integration support neuronal homology in annotated cross-species datasets. Related to Figure 1.
(**A**) Barplots depicting all 1:1:1 orthologs (y-axis) in the top-10 of differential genes of a neuron class in any of the three species. Colors within bars depict the gene subsets found in the top-20 differential genes in all three species (dark purple), in two species (light purple) or in one species (grey).
(**B**) Integration of scRNA-seq datasets of the three species using SAMap. Left – UMAP projections by species and by neuron class. Right - Heatmap of cell type mapping scores.
(**C**) Integration using Monocle3/Batchelor. Up – UMAP projections by species and by neuron class. Bottom – barplots depicting, in each species (color) and each neuron class (x-axis), the percentage of cells assigned to the “matching” cluster after integration (i.e., the cluster that contains the highest number of cells from the corresponding cell type across all datasets).

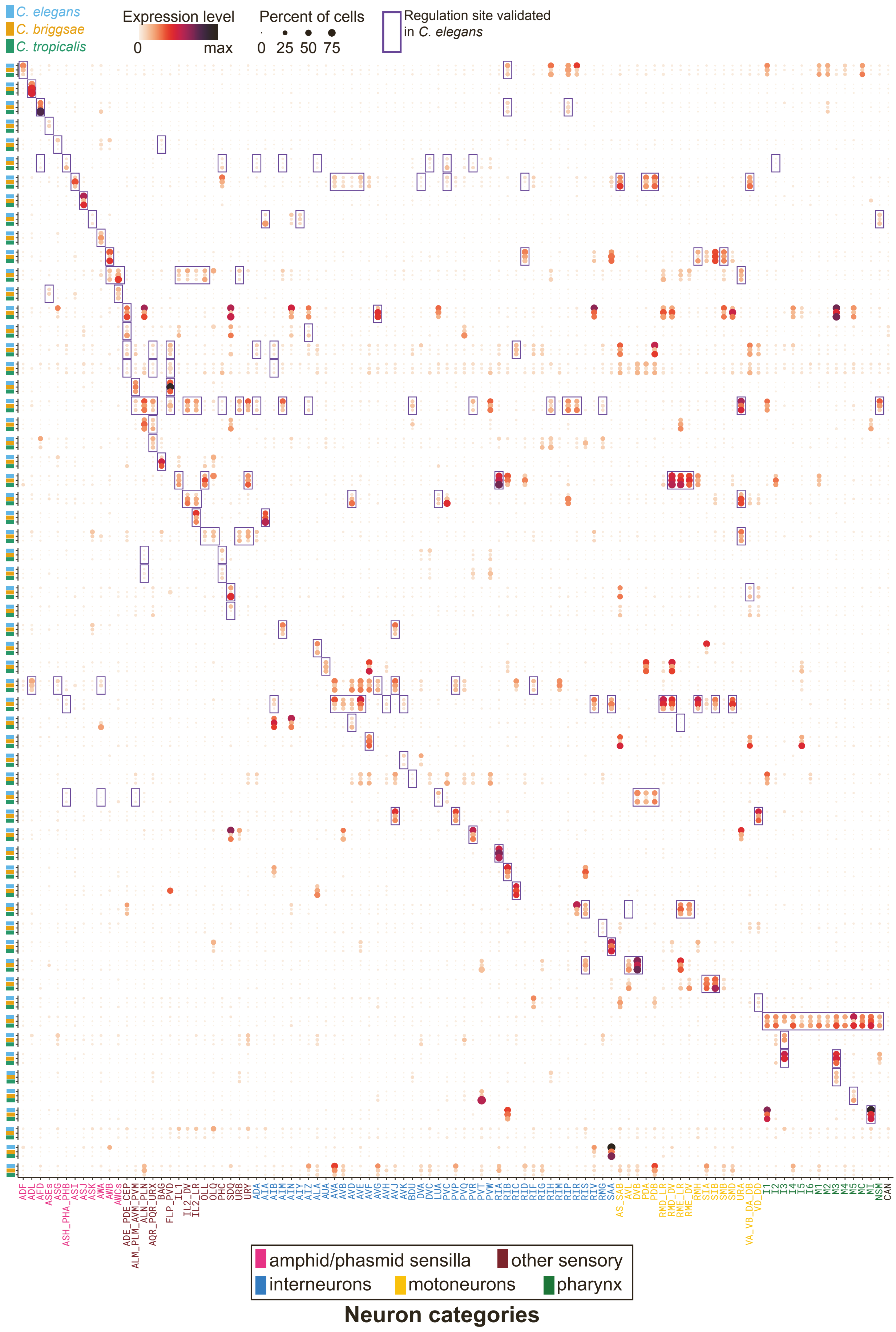
C. elegans
C. briggsae
C. tropicalis
Expression level
0
max
Percent of cells
0 25 50 75
Regulation site validated in C. elegans
lag-1
hlh-4
ttx-1
che-1
ceh-37
ceh-14
unc-3
sptf-1
ttx-3
odr-7
lim-4
sox-2
ceh-36
ast-1
ceh-43
unc-62
ceh-20
mec-3
unc-86
ahr-1
egl-13
ets-5
ceh-32
cfi-1
unc-39
vab-3
nob-1
php-3
lin-39
mab-5
mls-2
ceh-17
ceh-6
lin-11
unc-42
tab-1
unc-4
hlh-15
pag-3
egl-5
unc-30
ceh-31
ceh-8
aptf-1
hlh-13
nhr-67
ceh-13
sox-3
lim-6
ceh-24
elt-1
ceh-34
pros-1
ceh-2
mnm-2
vab-15
ceh-45
egl-18
ceh-9
sem-4
ADF ADL AFD ASEs ASG ASH_PHA_PHB ASI ASJ ASK AWA AWB AWCs ADE_PDE_CEP ALM_PLM_AVM_PVM ALN_PLN AQR_PQR_URX BAG FLP_PVD IL1 IL2_DV IL2_LR OLL OLQ PHC SDQ URB URY ADA AIA AIB AIM AIN AIY AIZ ALA AUA AVA AVB AVD AVE AVF AVG AVH AVJ AVK BDU DVA DVC LUA PVC PVP PVQ PVR PVT PVW RIA RIB RIC RID RIF RIG RIH RIM RIP RIR RIS RIV RMG SAA AS_SAB AVL DVB PDA PDB RMD_LR RMD_DV RME_LR RME_DV RMH SIA SIB SMB SMD URA VA_VB_DA_DB VD_DD I1 I2 I3 I4 I5 I6 M1 M2 M3 M4 M5 MC MI NSM CAN
amphid/phasmid sensilla
other sensory
interneurons
motoneurons
pharynx
Neuron categories

Figure S2. Expression conservation of transcription factors composing the regulatory codes of neuronal identity in *C. elegans*. Related to Figure 2.

Cross-species expression dotplot of all 1:1:1 transcription factor orthologs functionally validated as identity specifiers in *C. elegans*. Nematode species (y-axis) and neuron class (x-axis) are color-coded according to legend. Dot size represents the fraction of cells expressing the gene in a given neuron class, color represents scaled average expression levels. Experimentally-validated sites of identity regulation by a given transcription factor in *C. elegans* are marked with a rectangle. A subset of genes and cells also appear in Figure 1F. Data for ASE and I3 neurons is available for *C. elegans* and *C. briggsae* datasets only. Data for *ceh-9* (that regulate PVN neurons) and for *egl-18* & *sem-4* (that regulate HSN neurons) are shown, but their corresponding neurons are not part of the dataset. PVN are born during L2, and HSN neurons acquire many of their class-specific identity features in the L4 larval stage.

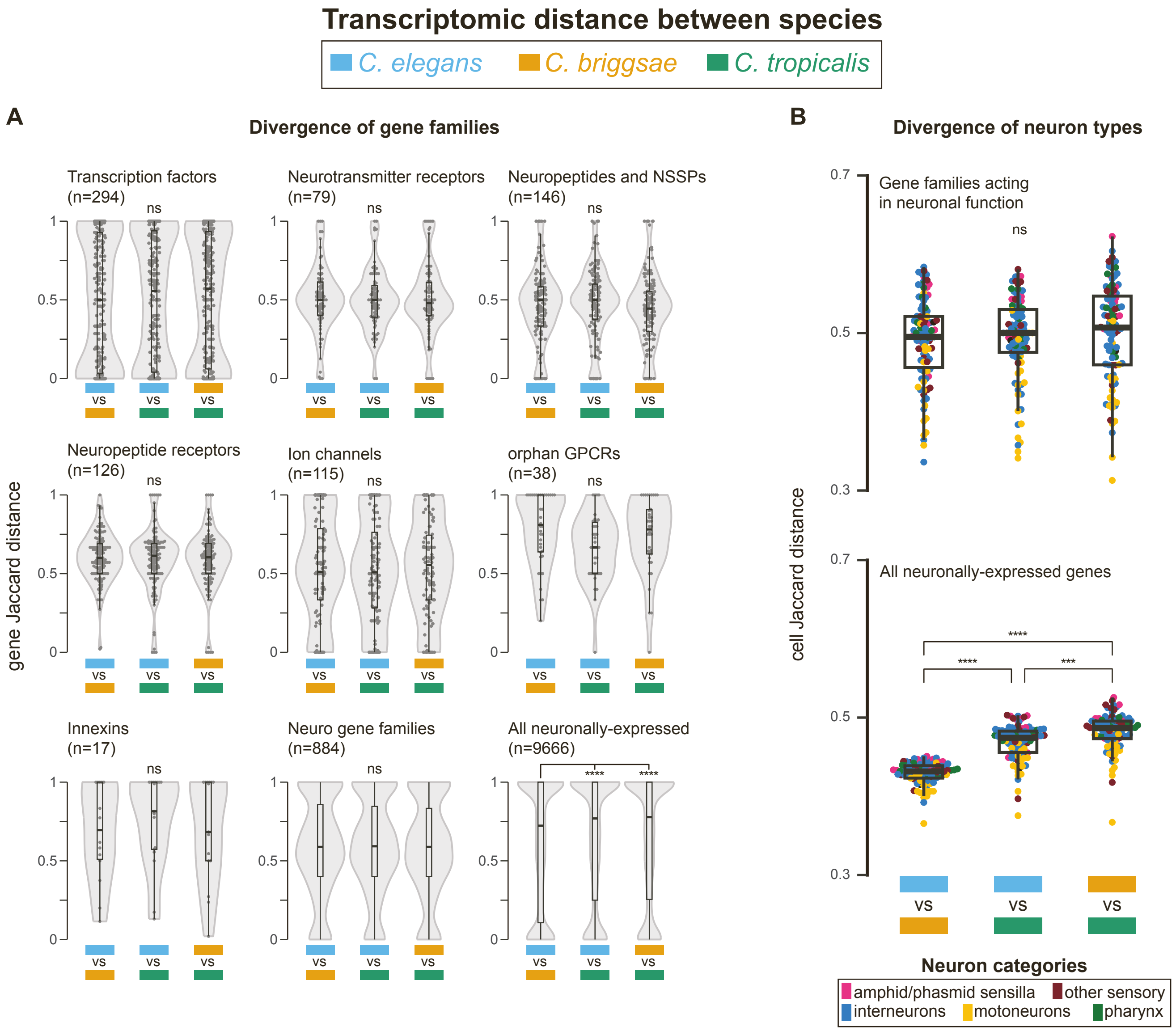

Figure S3. Transcriptomic comparisons between pairs of species. Related to Figure 2.

(A) Jaccard distance (y-axis) of genes (dots) grouped in families, reflecting divergence between pairs of species in their neuron-type-specific gene expression. Inquired gene families appear above corresponding panel. Pairs of species are color-coded below panels. n = number of genes in family, only 1:1 orthologs with expression in at least one neuron type were included. “Transcription factors” included only the families depicted in Figure 2E.

(B) Jaccard distances (y-axis) of neuron classes (dots) grouped into functional categories (x-axis and colors). The calculation of Jaccard distances included (upper panel) to gene families with established function in the nervous system or (lower panel) 9666 genes expressed in at least one species anywhere in the nervous system. Boxplots are Tukey-style. Kruskal-Wallis test, Dunn’s post hoc, Benjamini-Hochberg correction for multiple comparisons. ****P < 0.0001, ***P < 0.001, **P < 0.01, *P < 0.025.

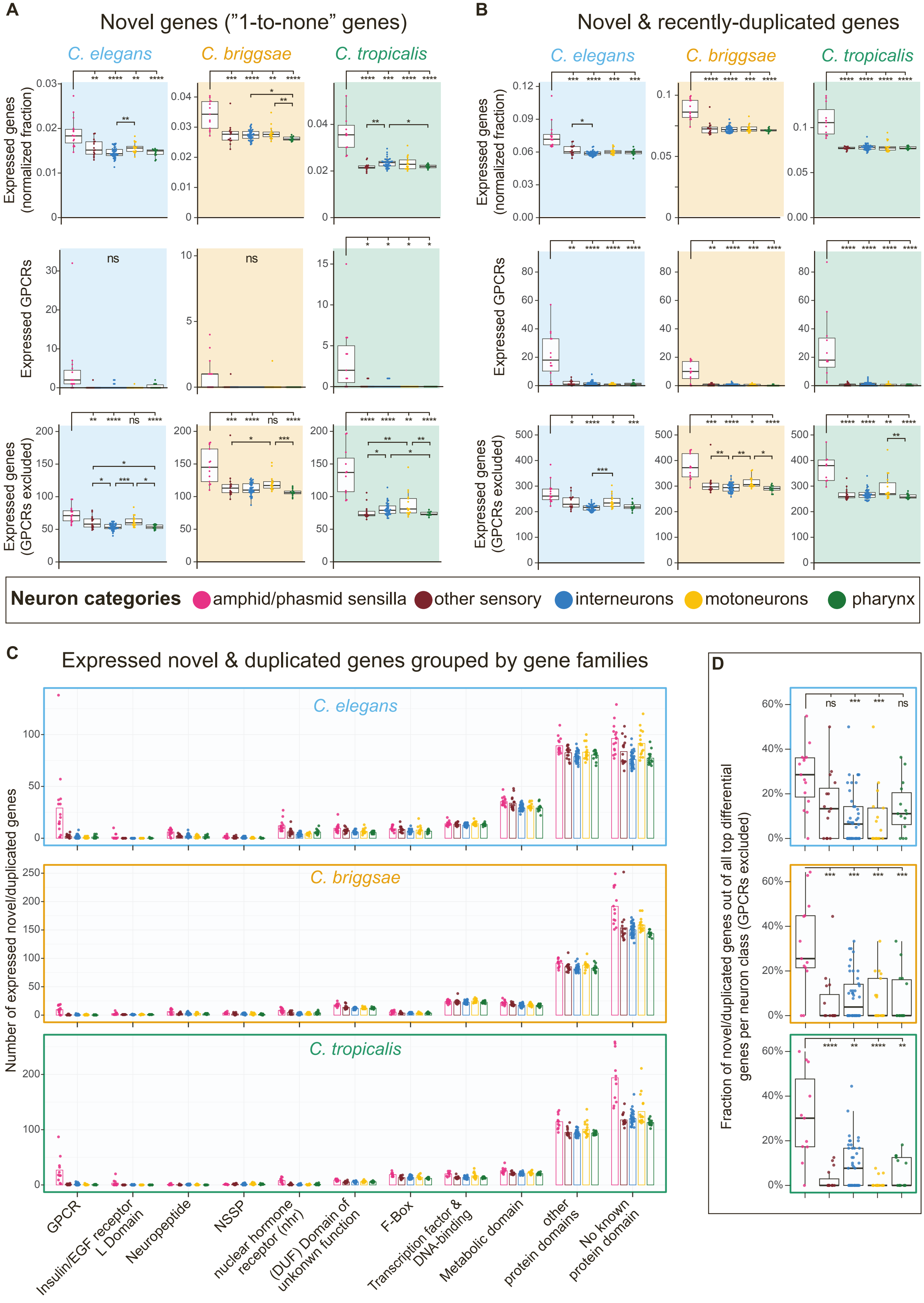

A
Novel genes ("1-to-none" genes)
C. elegans
C. briggsae
C. tropicalis
Expressed genes (normalized fraction)
Expressed GPCRs
Expressed genes (GPCRs excluded)
B
Novel & recently-duplicated genes
C. elegans
C. briggsae
C. tropicalis
Expressed genes (normalized fraction)
Expressed GPCRs
Expressed genes (GPCRs excluded)
Neuron categories
amphid/phasmid sensilla
other sensory
interneurons
motoneurons
pharynx
C
Expressed novel & duplicated genes grouped by gene families
C. elegans
C. briggsae
C. tropicalis
Number of expressed novel/duplicated genes
GPCR
Insulin/EGF receptor L Domain
Neuropeptide
NSSP
nuclear hormone receptor (nhr)
(DUF) Domain of unkonwn function
F-Box
Transcription factor & DNA-binding
Metabolic domain
other protein domains
No known protein domain
D
Fraction of novel/duplicated genes out of all top differential genes per neuron class (GPCRs excluded)

Figure S4. Enrichment of novel and duplicated genes in sensory neurons of the amphid and phasmid sensilla. Related to Figure 3.

(**A)** Expression of novel genes (“1-to-none” orthologs, y-axis) in different neuron classes and species (thresholded expression). Top panels: fraction of expressed novel genes normalized to the total number of expressed genes per cell type. Middle panel: number of novel GPCR genes expressed per cell type. Bottom panels: number of expressed genes after exclusion of all GPCRs from analysis. Neuron classes were grouped into functional categories (x-axis and color coded), each dot represents a single neuron class. Boxplots are Tukey-style.
(**B**) Expression of novel and recently-duplicated genes (“1-to-none”, “1-to-many”, “many-to-many” orthologs) in different neuron classes and species. Panels similar to (A).
(**C**) Number of expressed novel and recently-duplicated genes (y-axis) belonging to different gene families (x-axis) based on sequence homology or the presence of key protein domains. Neuron classes were grouped into functional categories, each dot represents a single neuron class, bars depict mean values for all neurons in category. Genes with no domains detected in PFAM are in the category “no known protein domain”.
(**D**) Proportions of novel and duplicated genes out of all differentially-expressed genes per neuron class, grouped by neuron functional category. All GPCRs were excluded from the analysis.
Statistical tests: Kruskal-Wallis test, Dunn’s post hoc, Benjamini-Hochberg correction for multiple comparisons. ****$P < 0.0001$, ***$P < 0.001$, **$P < 0.01$, *$P < 0.025$.

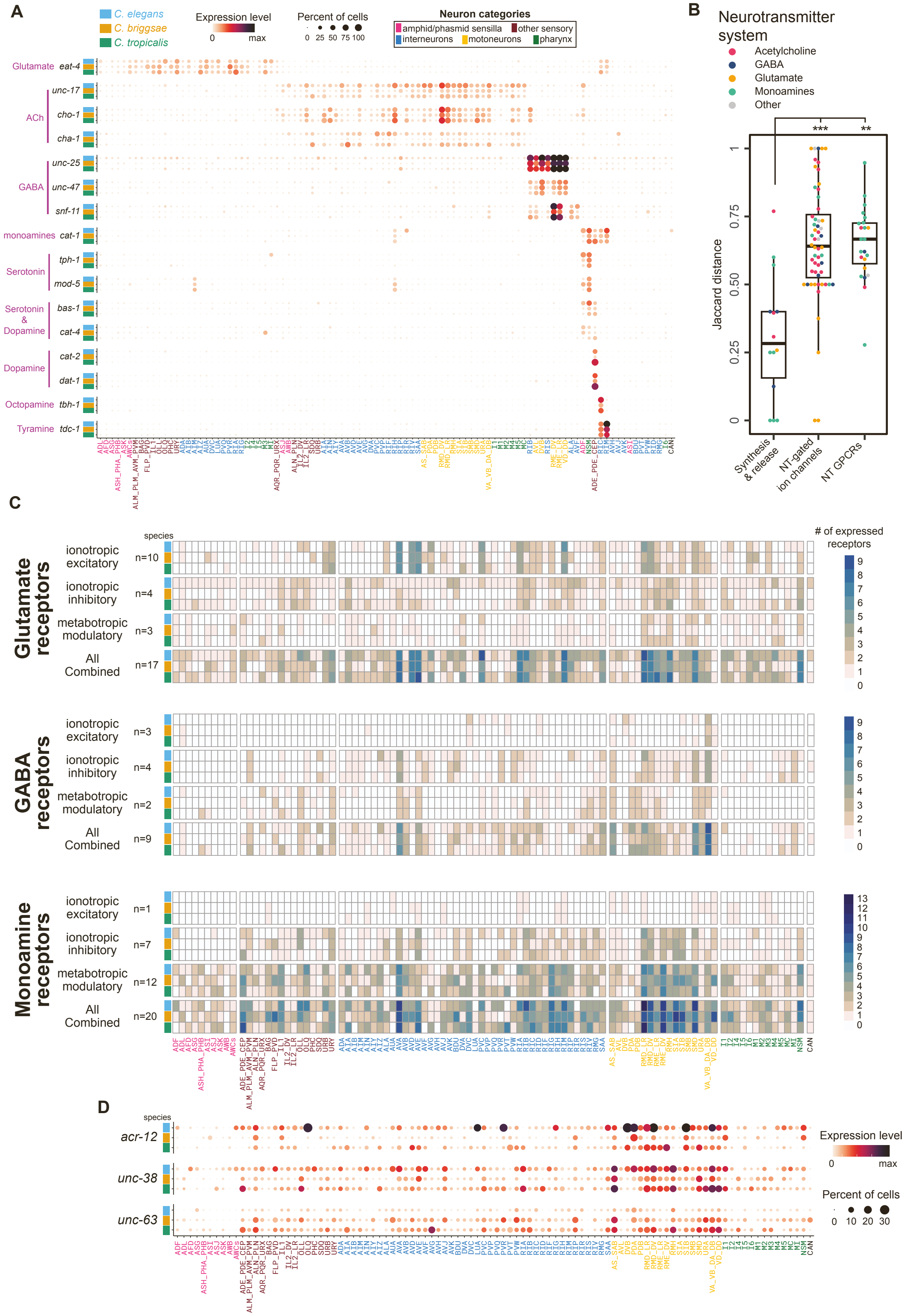
A
C. elegans
C. briggsae
C. tropicalis
Expression level
0
max
Percent of cells
0 25 50 75 100
Neuron categories
amphid/phasmid sensilla
other sensory
interneurons
motoneurons
pharynx
Glutamate
eat-4
ACh
unc-17
cho-1
cha-1
GABA
unc-25
unc-47
snf-11
monoamines
cat-1
Serotonin
tph-1
mod-5
Serotonin & Dopamine
bas-1
cat-4
Dopamine
cat-2
dat-1
Octopamine
tbh-1
Tyramine
tdc-1
B
Neurotransmitter system
Acetylcholine
GABA
Glutamate
Monoamines
Other
Jaccard distance
0
0.25
0.50
0.75
1
***
**
Synthesis & release
NT-gated ion channels
NT GPCRs
C
species
Glutamate receptors
GABA receptors
Monoamine receptors
ionotropic excitatory
ionotropic inhibitory
metabotropic modulatory
All Combined
n=10
n=4
n=3
n=17
n=3
n=4
n=2
n=9
n=1
n=7
n=12
n=20
# of expressed receptors
D
species
acr-12
unc-38
unc-63
Expression level
0
max
Percent of cells
0 10 20 30

Figure S5. Neuron-type-specific expression of gene modules determining the identity of emitted and received neurotransmitters across species. Related to Figure 4.

(**A)** Cross-species expression dotplot of neurotransmitter synthesis genes, neurotransmitter vesicular transporter genes and neurotransmitter uptake transporter genes determining neurotransmitter release identity of neuronal cell classes. Data for *eat-4*, *unc*-17, *unc-25* & *cat-1* appear in Figure 4B and are shown again here for ease of visualization. Associated neurotransmitters are labeled in purple (y-axis). Ach: Acetylcholine; GABA: Gamma-aminobutyric acid. Nematode species (y-axis) and neuron class (x-axis) are color-coded according to legend. Dot size represents the fraction of cells expressing the gene in a given neuron class, color represents scaled average expression levels.

(**B**) Jaccard distances of 1:1:1 ortholog genes determining neurotransmitter release identity and receptivity to neurotransmitters. Each dot represents a gene, boxplots are Tukey-style. Data is a subset of the data already appearing in Figure 4A, but each gene is color-coded here according to the neurotransmitter system to which it belongs. Kruskal-Wallis test, Dunn's post hoc, Benjamini-Hochberg correction for multiple comparisons. ***P < 0.001, **P < 0.01.

(**C)** Heatmaps representing the number (color-coded) of neurotransmitter receptors expressed in each neuron class (x-axis) across species (y-axis). Subtype of receptors (ionotropic excitatory, ionotropic inhibitory, metabotropic modulatory) are indicated to the left of heatmaps. N values indicate the total number of receptors (1:1:1 orthologs) included in the analysis for a given subtype. Top – Glutamate receptors; Middle – GABA receptors; Bottom – Monoamine receptors.Receptors for all known monoamines acting in *C. elegans* (serotonin, dopamine, tyramine, octopamine) were considered combined together as a single category.

(**D**) Cross-species expression dotplot of the acetylcholine receptors *acr-12, unc-38* & *unc-63*. These receptors are broadly expressed throughout the nervous system.

A

NPP-GPCR	*C. elegans*	*C. briggsae*	*C. tropicalis*
NLP-13 DMSR-2	pervasive	integrative	pervasive
FLP-1 DMSR-6	local	local	broadcaster
FLP-4 DMSR-7	broadcaster	pervasive	pervasive
FLP-7 DMSR-7	pervasive	broadcaster	pervasive
FLP-12 DMSR-8	local	broadcaster	broadcaster
FLP-4 EGL-6	local	integrative	integrative
FLP-6 EGL-6	local	integrative	integrative
FLP-8 FRPR-15	local	local	broadcaster
FLP-3 FRPR-16	broadcaster	local	local
FLP-1 FRPR-7	local	broadcaster	broadcaster
FLP-4 FRPR-8	local	integrative	integrative
FLP-21 NPR-1	local	broadcaster	broadcaster
FLP-21 NPR-2	broadcaster	local	local
FLP-15 NPR-3	pervasive	integrative	pervasive
NLP-10 NPR-35	local	integrative	integrative
NLP-13 NPR-41	integrative	pervasive	no network
NLP-38 SPRR-2	integrative	pervasive	pervasive
NLP-58 TKR-1	local	broadcaster	integrative
NLP-58 TKR-2	local	local	integrative

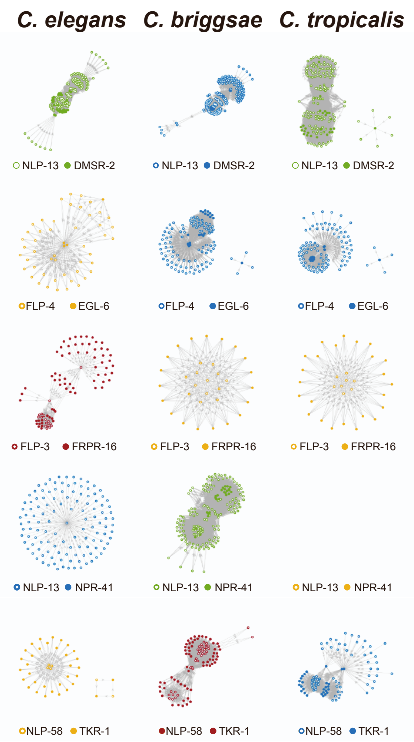

B

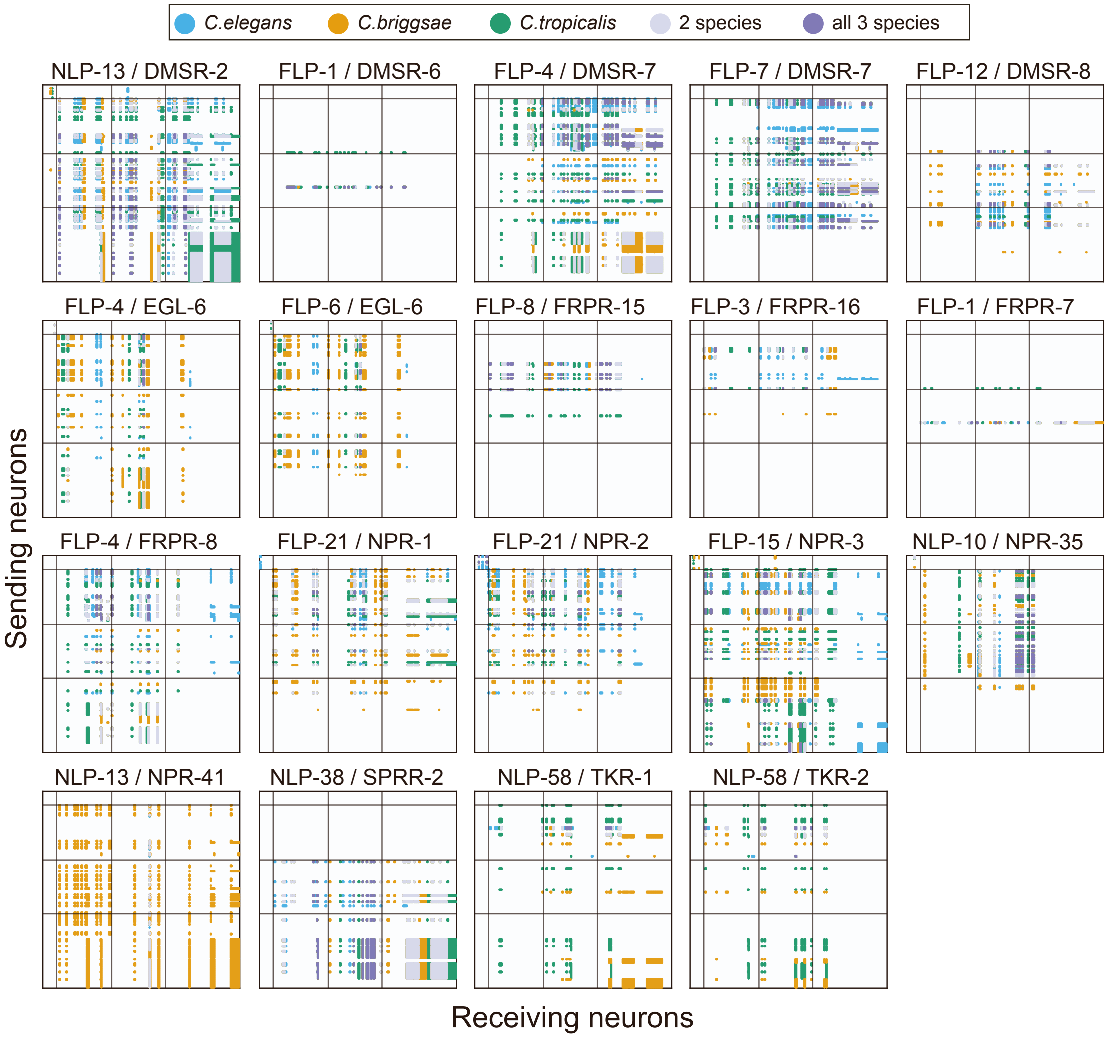

Figure S6. Neuropeptide-receptor pairs displaying divergent network topologies across species. Related to Figure 5.
(**A**) 19 NPP-GPCR pairs displayed divergent topologies across species. Local networks (yellow) express the NPP and GPCR in ≤50 neurons. Pervasive networks (green) express both in ≥50 neurons. Broadcasting networks (red) express the NPP in ≤50 and the GPCR in ≥50. Integrative networks (blue) express the NPP ≥50 neurons and the GPCR in ≤50. Right: Graph visualizations of 4 NPP-GPCR networks across species.
(**B**) Adjacency matrix representation of the 19 individual NPP-GPCR pairs described above (short-range networks). Rows, columns and separations are similar to Figure 5E.

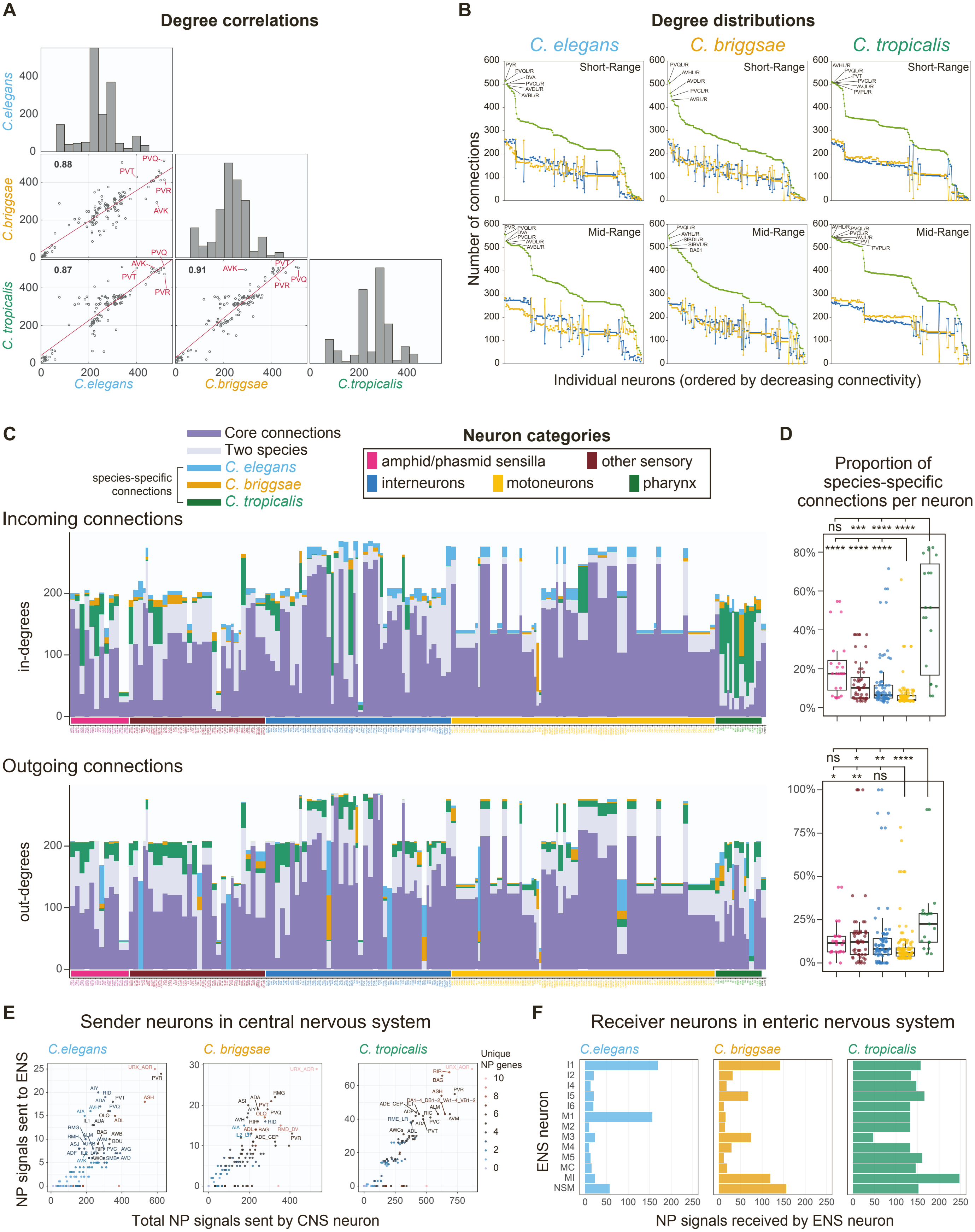
A
Degree correlations
C.elegans
C.briggsae
C. tropicalis
0.88
0.87
0.91
PVQ
PVT
PVR
AVK
B
Degree distributions
C. elegans
C. briggsae
C. tropicalis
Short-Range
Mid-Range
Number of connections
Individual neurons (ordered by decreasing connectivity)
C
Core connections
Two species
species-specific connections
Neuron categories
amphid/phasmid sensilla
other sensory
interneurons
motoneurons
pharynx
Incoming connections
in-degrees
Outgoing connections
out-degrees
D
Proportion of species-specific connections per neuron
ns
E
Sender neurons in central nervous system
NP signals sent to ENS
Total NP signals sent by CNS neuron
Unique NP genes
F
Receiver neurons in enteric nervous system
ENS neuron
NP signals received by ENS neuron
I1
I2
I4
I5
I6
M1
M2
M3
M4
M5
MC
MI
NSM

Figure S7. Degree analysis and communication between central and enteric nervous systems through neuropeptidergic networks. Related to Figure 5. Peptidergic degree is defined as the number of incoming and outgoing connections per neuron (the sum of in-degrees and out-degrees).

(**A**) Pearson correlations of degrees between homologous neuron classes in pairs of species (short-range networks). The conserved peptidergic hubs are highlighted in red (AVK, PVR, PVQ, PVT).

(**B**) Distributions of degrees in the neuropeptidergic networks of three nematode species. Top panels: short-range networks. Bottom panels: mid-range networks. Degree (incoming plus outgoing connections) is shown in green, in-degree (incoming connections) in blue and out-degree (outgoing connections) in yellow. The 10 highest-degree hubs in each network are indicated.

(**C**) Total number of degrees (y-axis) in homologous neurons across species (x-axis). Top panel: in-degrees. Bottom panel: out-degrees. Bars are color-filled according to the subsets of core degrees and species-specific degrees of the neuron across species.

(**D**) Proportions of species-specific degrees (y-axis) per neuron classified by functional categories (colors and x-axis). Kruskal-Wallis test, Dunn's post hoc, Benjamini-Hochberg correction for multiple comparisons. ****$P < 0.0001$, ***$P < 0.001$, **$P < 0.01$, *$P < 0.025$.

(**E**) Weighted numbers of neuropeptide signals sent from neurons in the central nervous system to the enteric nervous system (y-axis) vs total numbers sent overall (x-axis). Coloring of dots represent the number of unique neuropeptide genes which bind GCPRs that are expressed in the enteric nervous system.

(**F**) Number of received neuropeptide signals per neuron type in the enteric nervous system.

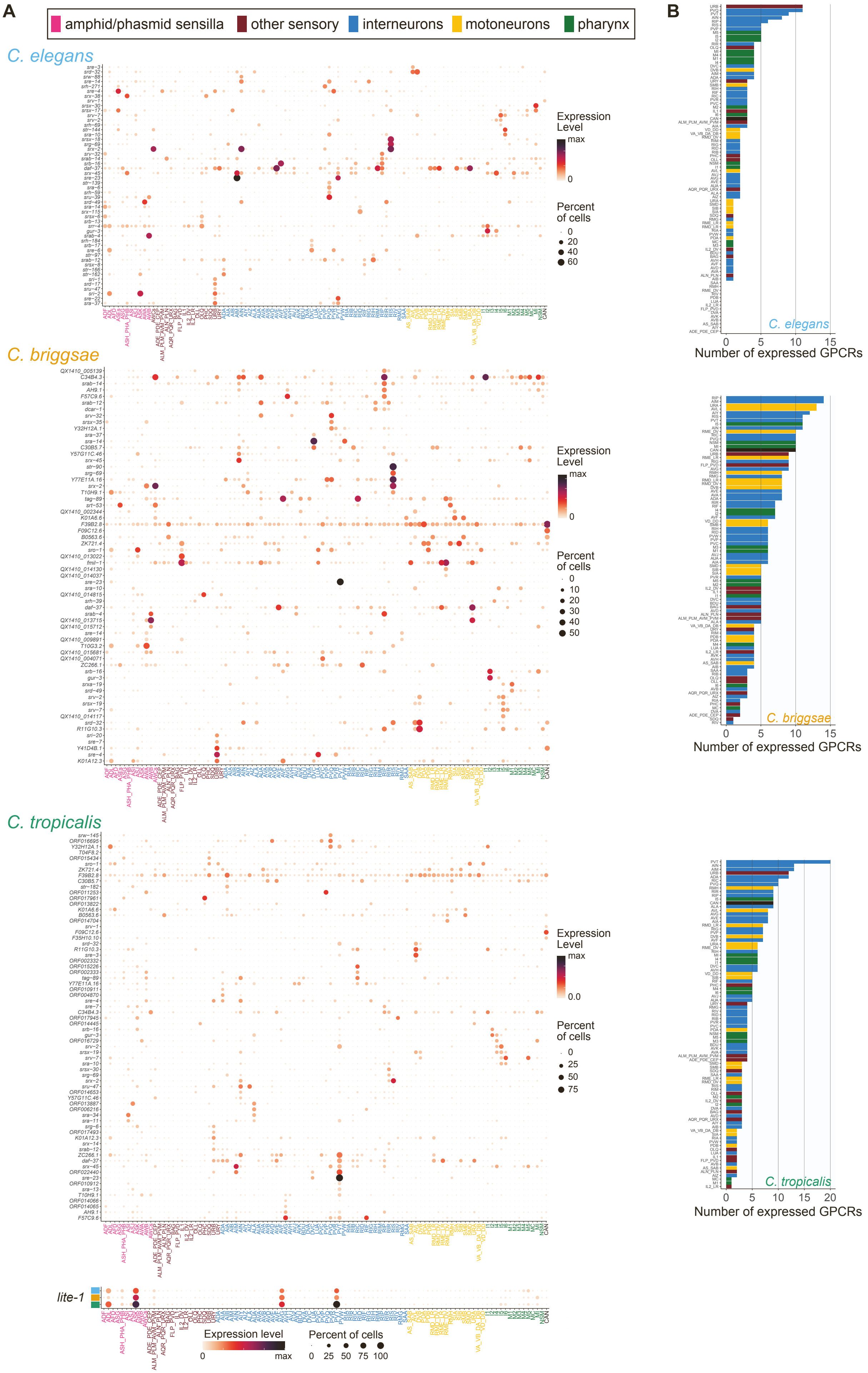
A
amphid/phasmid sensilla
other sensory
interneurons
motoneurons
pharynx
C. elegans
C. briggsae
C. tropicalis
Expression Level
max
0
Percent of cells
lite-1
B
Number of expressed GPCRs

Figure S8. GPCRs expressed in non-sensory neurons. Related to Figure 6.
(**A**) Expression dotplots of GPCR genes that are expressed outside of the amphid/phasmid sensilla and have no sequence homology with neuropeptide- and neurotransmitter-binding GPCRs. 51 GPCRs pass the criteria in *C. elegans*, 63 in *C. briggsae* and 69 in *C. tropicalis*. In the *C. briggsae* and *C. tropicalis* panels, 1:1 orthologs of *C. elegans* genes appear with the *C. elegans* gene name (y-axis). Bottom: The light-responsive *lite-1* GPCR of the gustatory-receptor family is expressed at much higher levels than all other listed GPCRs, and is displayed separately. Dot size represents the fraction of cells expressing the gene in a given neuron class, color represents scaled average expression levels.
(**B**) Barplots indicating how many of these GPCRs are expressed in each neuron class. Bars are colored according functional categories, sensory neurons of the amphid and phasmid sensilla were excluded.

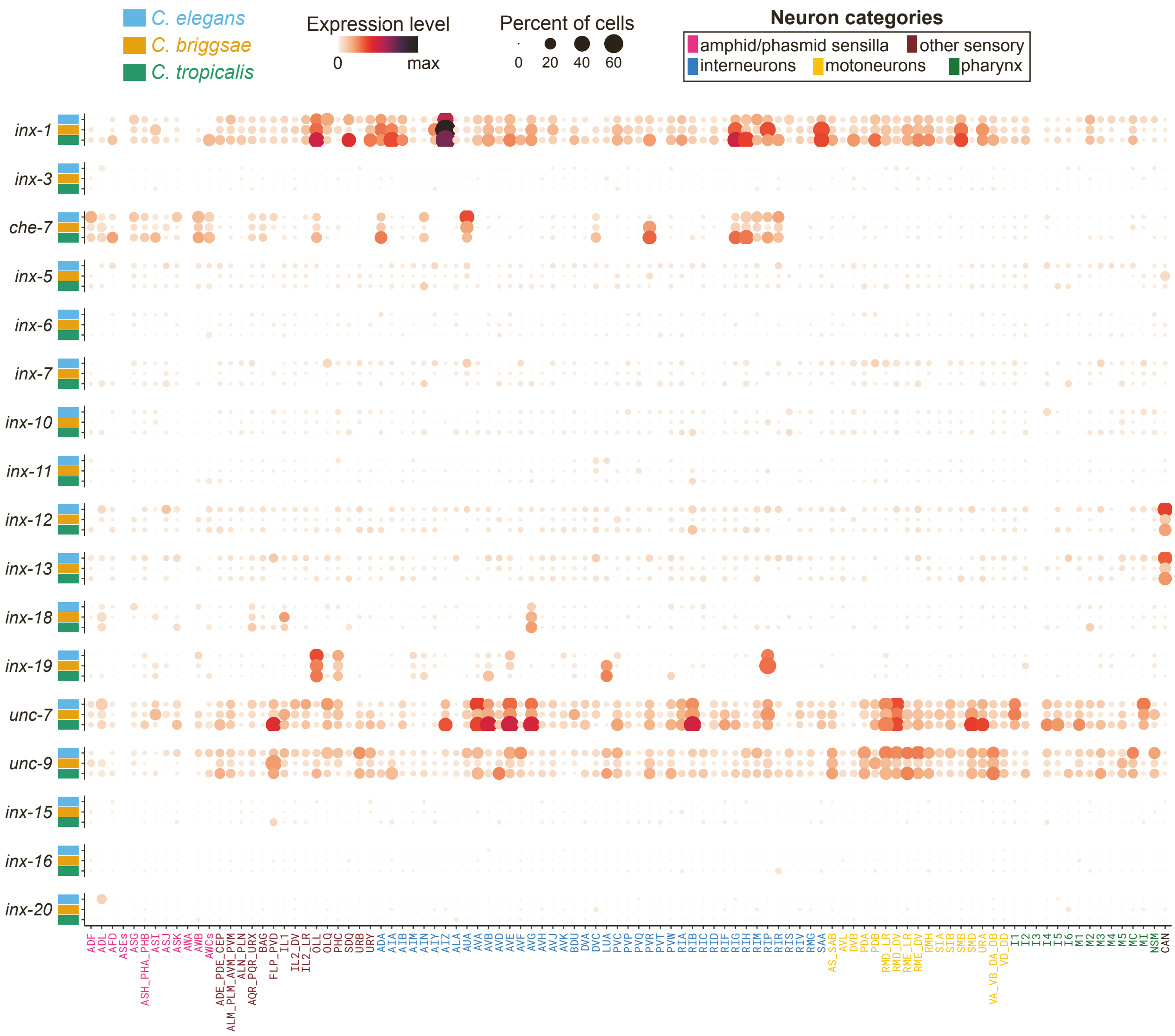

Figure S9. Expression profile of innexins in the nervous system. Related to Figure 6.
Cross-species expression dotplot of innexin 1:1:1 orthologs in the nervous system. Genes with no expression in the nervous system were excluded. Nematode species (y-axis) and neuron class (x-axis) are color-coded according to legend. Dot size represents the fraction of cells expressing the gene in a given neuron class, color represents scaled average expression levels.

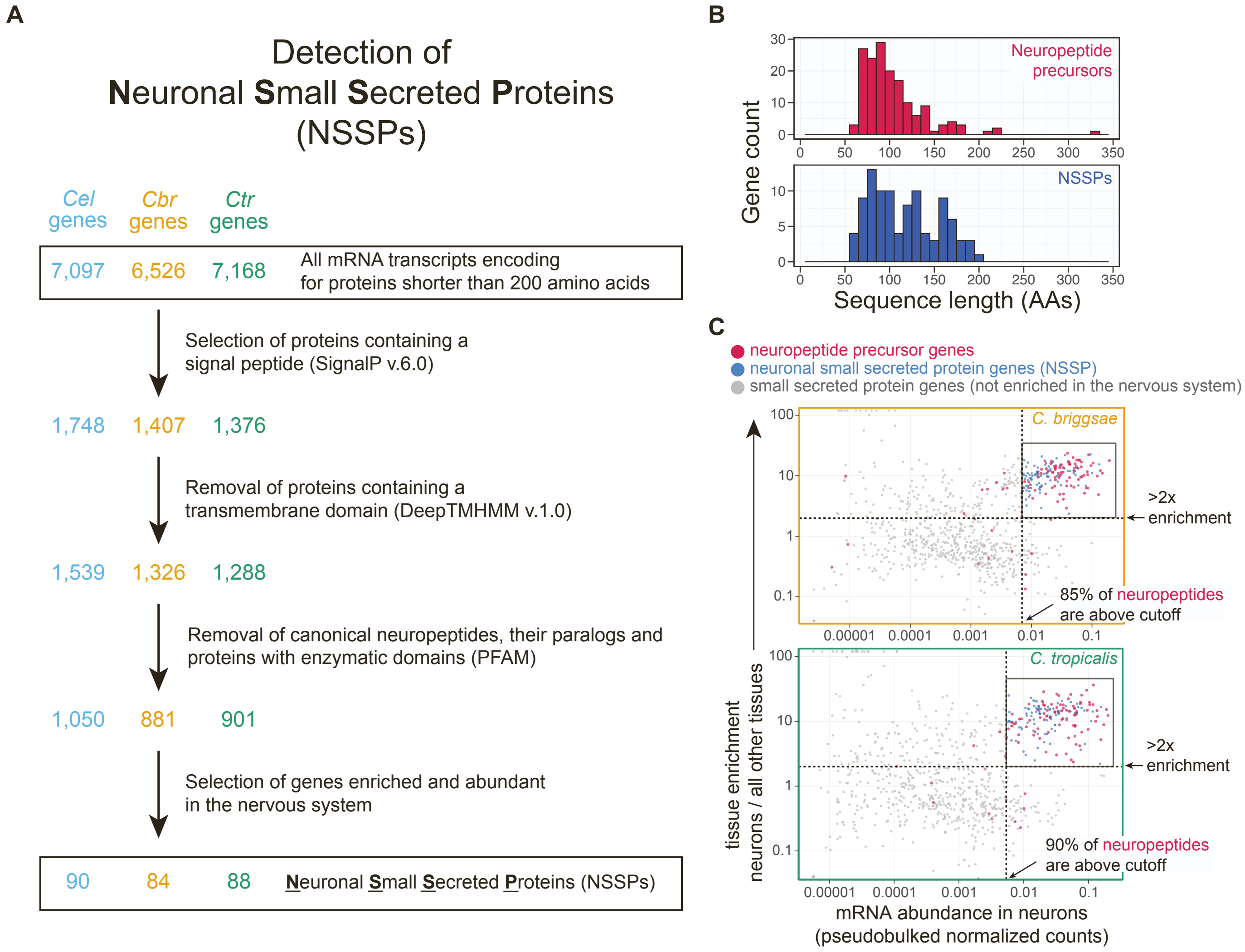

Figure S10. Detection of Neuronal Small Secreted Proteins (NSSPs). Related to Figure 7.

(**A**) Diagram depicting the filtering process defining the pools of NSSPs. Numbers of genes remaining after each step are shown.

(**B**) Distributions of amino acid sequence lengths of all known *C. elegans* neuropeptide precursor genes (red) and newly-defined *C. elegans* NSSPs (blue). Longest mRNA isoform for each gene was used. Only 4/161 (2.5%) of all neuropeptide genes encode for proteins longer than 200 amino acids.

(**C**) Neuronal enrichment (y-axis) and neuronal expression levels (x-axis) of genes encoding small secreted proteins in *C. briggsae* (top panel) and *C. tropicalis* (bottom panel). Each dot is a gene, neuropeptides (red) and NSSPs (blue) are colored. Dashed lines: cutoff criteria used to delineate NSSPs.

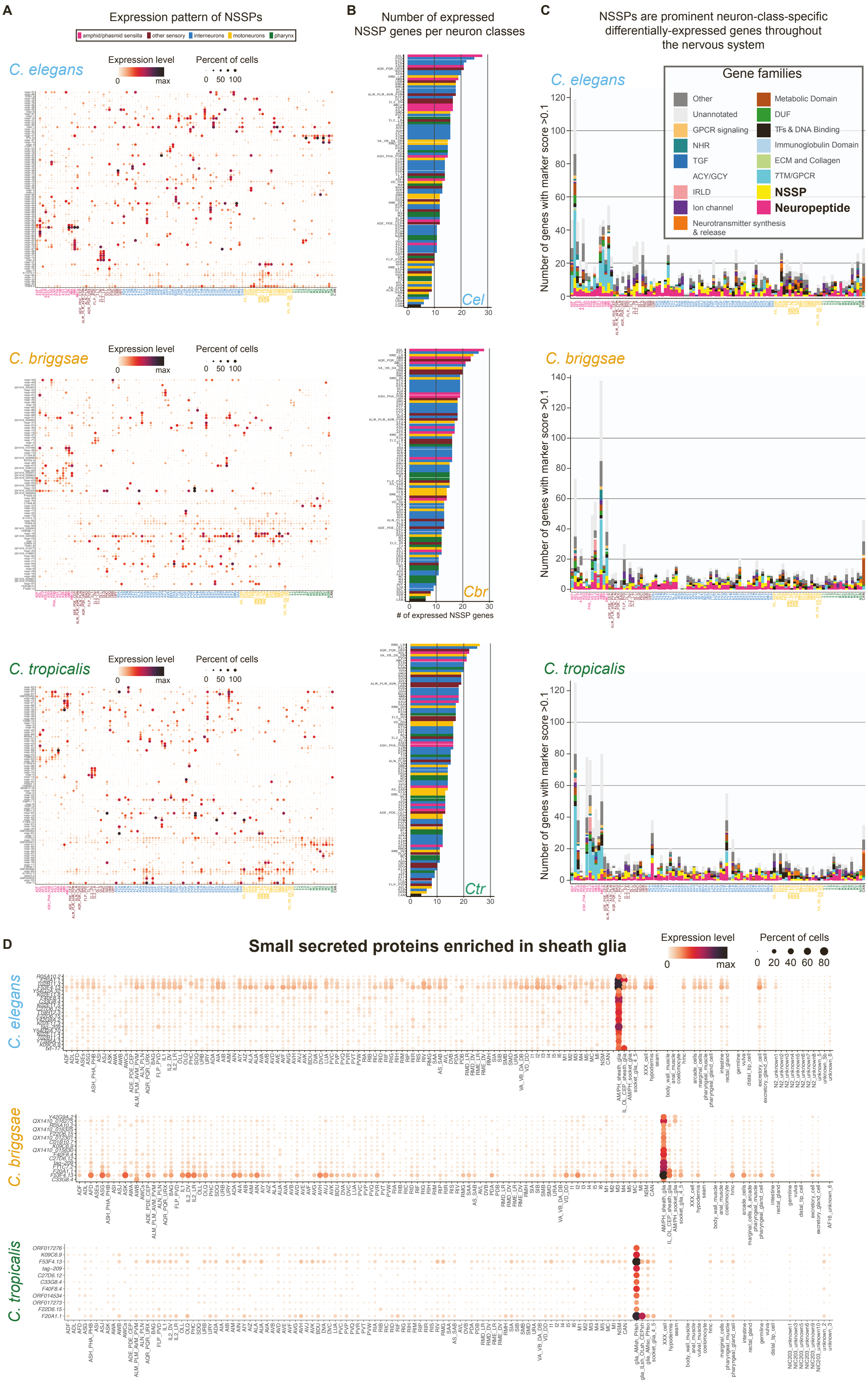
A
Expression pattern of NSSPs
amphid/phasmid sensilla
other sensory
interneurons
motoneurons
pharynx
C. elegans
Expression level
0
max
Percent of cells
0 50 100
C. briggsae
C. tropicalis
B
Number of expressed NSSP genes per neuron classes
Cel
Cbr
Ctr
# of expressed NSSP genes
C
NSSPs are prominent neuron-class-specific differentially-expressed genes throughout the nervous system
Gene families
Other
Unannotated
GPCR signaling
NHR
TGF
ACY/GCY
IRLD
Ion channel
Neurotransmitter synthesis & release
Metabolic Domain
DUF
TFs & DNA Binding
Immunoglobulin Domain
ECM and Collagen
7TM/GPCR
NSSP
Neuropeptide
Number of genes with marker score >0.1
D
Small secreted proteins enriched in sheath glia
Expression level
Percent of cells
0 20 40 60 80

Figure S11. NSSPs are a key distinctive molecular feature in most neuron classes. Related to Figure 7.
(**A**) Expression dotplots of NSSP genes in *C. elegans* (n=90 genes), *C. briggsae* (n=84) and *C. tropicalis* (n=88). In the *C. briggsae* and *C. tropicalis* panels, 1:1 orthologs of *C. elegans* genes appear with the *C. elegans* gene name (y-axis). Dot size represents the fraction of cells expressing the gene in a given neuron class, color represents scaled average expression levels.
(**B**) Barplots showing how many NSSPs are expressed in each neuron class. Bars are colored according functional categories of neurons.
(**C**) Barplots representing the numbers (y-axis) of differentially-expressed genes (*top_markers* score >0.1) expressed in each neuron class (x-axis). Genes with high marker scores tend to be abundantly and specifically expressed in one or few neuron classes. The fraction of genes belonging to specific gene families are colored according to legend. Neuropeptides (**magenta**) and NSSPs (**yellow**) appear among the most differentially-expressed genes in a majority of neuron classes throughout the nervous system. "NHR": nuclear hormone receptor; "TGF": Transforming Growth Factor family; "ACY/GCY" adenylate and guanylyl cyclases; "IRLD": insulin/EGF receptor L Domain family; "DUF": Domain of Unknown Function, "ECM": extracellular matrix, "Unannotated": genes with no domains detected by PFAM.
(**D**) Expression dotplots of small secreted proteins with no characterized protein domain and enriched in sheath glia. In the *C. briggsae* and *C. tropicalis* panels, 1:1 orthologs of *C. elegans* genes appear with the *C. elegans* gene name (y-axis). Dot size represents the percentage of cells expressing the gene in a given cell type, color represents scaled average expression levels.
